# Family focused interventions that address parental domestic violence and abuse, mental ill-health, and substance misuse in combination: A systematic review

**DOI:** 10.1371/journal.pone.0270894

**Published:** 2022-07-29

**Authors:** Kate Allen, G. J. Melendez-Torres, Tamsin Ford, Chris Bonell, Katie Finning, Mary Fredlund, Alexa Gainsbury, Vashti Berry

**Affiliations:** 1 College of Medicine and Health, University of Exeter, Exeter, United Kingdom; 2 Department of Psychiatry, University of Cambridge, Cambridge, United Kingdom; 3 London School of Hygiene and Tropical Medicine, London, United Kingdom; 4 UK Health Security Agency, Totnes, United Kingdom; South African Medical Research Council, SOUTH AFRICA

## Abstract

Parental domestic violence and abuse (DVA), mental ill-health (MH), and substance misuse (SU) are three public health issues that tend to cluster within families, risking negative impacts for both parents and children. Despite this, service provision for these issues has been historically siloed, increasing the barriers families face to accessing support. Our review aimed to identify family focused interventions that have combined impacts on parental DVA, MH, and/or SU. We searched 10 databases (MEDLINE, PsycINFO, Embase, CINAHL, Education Research Information Centre, Sociological Abstracts, Applied Social Sciences Index & Abstracts, ProQuest Dissertations and Theses Global, Web of Science Core Collection, and Cochrane Central Register of Controlled Trials) from inception to July 2021 for randomised controlled trials examining the effectiveness of family focused, psychosocial, preventive interventions targeting parents/carers at risk of, or experiencing, DVA, MH, and/or SU. Studies were included if they measured impacts on *two or more* of these issues. The Cochrane Risk of Bias Tool 2 was used to quality appraise studies, which were synthesised narratively, grouped in relation to the combination of DVA, MH, and/or SU outcomes measured. Harvest plots were used to illustrate the findings. Thirty-seven unique studies were identified for inclusion. Of these, none had a combined positive impact on all three outcomes and only one study demonstrated a combined positive impact on two outcomes. We also found studies that had combined adverse, mixed, or singular impacts. Most studies were based in the U.S., targeted mothers, and were rated as ‘some concerns’ or ‘high risk’ of bias. The results highlight the distinct lack of evidence for, and no ‘best bet’, family focused interventions targeting these often-clustered risks. This may, in part, be due to the ways interventions are currently conceptualised or designed to influence the relationships between DVA, MH, and/or SU.

**Trial registration**: PROSPERO registration: CRD42020210350.

## Introduction

Parental domestic violence and abuse (DVA; defined as violence and abuse between parents/caregivers), mental ill-health (MH; defined as common mental health disorders experienced by parents/caregivers), and substance misuse (SU; defined as alcohol and drug use experienced by parents/caregivers) are three commonly experienced adverse childhood experiences (ACEs) in the UK [1–3] and worldwide [4–8] (see S1 Appendix for full definitions). There is evidence to suggest that DVA, MH, and SU not only co-occur (i.e., happen in the same time and space; [9]) but also cluster (i.e., are associated with one another, interact, and modify/reinforce the risk of the other occurring; [9]) [10–16]. Families experiencing a combination of these issues are likely to be particularly vulnerable and in need of targeted support [17, 18]. At a conservative estimate, 3.6% of children in the UK are living in households where all three issues are present [19], which has likely been exacerbated by COVID-19 and the resulting government-related restrictions [20–22]. This is concerning given that these issues can have a negative impact on parents’ health, parenting capacity [23, 24], and risk of child maltreatment [25–27]. Additionally, children experiencing these ACEs within the family are at increased risk of developing problems themselves with internalising and externalising behaviour during childhood [28] and violence, MH, and SU later on in life [24, 29, 30].

Although the clustering of risk is likely to require a response that addresses the mechanisms for these outcomes in combination [31], service provision and commissioning of services for DVA, MH, and SU remain largely siloed [32–34]. This creates additional barriers to access for families experiencing a combination of these issues and results in provision that fails to address the complexity of families’ needs [18, 35, 36]. In light of this, recent UK reports have emphasised a need for more interdisciplinary working between services targeting these issues, particularly within the family context [32, 36, 37]. This has led to initiatives such as the ‘Troubled Families’ programme [38, 39] and changes in the way some local authorities (LAs) commission services [40, 41]. For example, several LAs have created ‘group alliances’ funding services that respond to needs in multiple domains (see http://lhalliances.org.uk/).

While policy and practice communities are making strides to support families at risk of, or experiencing, clustered parental DVA, MH, and SU, evidence-based guidance for choice of intervention is lacking. Systematic reviews have tended to examine the effectiveness of family focused or psychosocial interventions targeting DVA, MH, and SU in isolation (e.g., [42, 43–45]). Promising approaches include advocacy, counselling/therapy, and skill-building for DVA [43, 46], counselling/therapy and home-based approaches for MH [45], and brief interventions, intensive case management, and motivational approaches for SU [44]. However, findings are often mixed or limited which may partly reflect failure to address co-occuring or clustering issues in combination [47]. Furthermore, studies and reviews that have examined combined impacts have focused on risk dyads in adults, such as DVA and MH [48], DV and SU [49], or MH and SU [50, 51], rather than all three combined or focusing on parents/families specifically. While recognising the limited evidence-base, such reviews have highlighted the potential importance of integrated interventions addressing issues in combination, trauma-informed approaches, and tailoring of interventions to meet individual needs.

This review aims to fill the gap in the evidence-base by examining whether interventions are effective in impacting outcomes in combination and, if so, what are the current ‘best bet’ family focused interventions. This review is the first of its kind and reflects current UK and global priorities for focusing on prevention [52, 53]. Our review aims to examine whether preventive, psychosocial, family focused interventions have combined impacts on parental DVA, MH, and/or SU.

## Methods

This systematic review is reported in line with PRISMA guidelines [54] (S1 Appendix). The protocol for this review was registered on PROSPERO (CRD42020210350) and the full protocol is publicly available on the first author’s staff profile page (https://arc-swp.nihr.ac.uk/about-penarc/people/kate-allen/).

### Eligibility criteria

Studies were eligible for inclusion if they met the following criteria: 1) employed a randomised controlled trial (RCT) design; 2) targeted a population that included parents/carers at risk of, or experiencing, one or more of DVA (restricted to physical, sexual, emotional, coercive control, or economic violence and abuse between parents/caregivers), MH (restricted to common mental health disorders experienced by parents/caregivers), and/or SU (restricted to alcohol and/or drug misuse or dependence experienced by parents/caregivers), or targeted the children in their care; 3) examined the effectiveness of an intervention that was family focused, psychosocial and preventive, aiming to prevent or reduce parental DVA, MH and/or SU or the negative impact of these experiences on the children in their care; and 4) measured two or more of the following outcomes: DVA (victimisation/perpetration between parents/caregivers), MH (depression, anxiety, PTSD, panic disorder, OCD, general mental health of parents/caregivers), or SU (alcohol, drug use, general SU of parents/caregivers) (S1 Appendix).

### Search strategy

Our search strategy was developed in consultation with AB, an information specialist within the PenARC evidence synthesis team at the University of Exeter, and was conducted by KA. We searched ten electronic databases (MEDLINE, PsycINFO, Embase, CINAHL, Education Research Information Centre (ERIC), Sociological Abstracts, Applied Social Sciences Index & Abstracts (ASSIA), ProQuest Dissertations and Theses Global, Web of Science Core Collection, and Cochrane Central Register of Controlled Trials (CENTRAL)) from inception to March 2020. Our search terms fell into five main categories combined as follows: [DVA OR MH OR SU] AND parents/family AND RCTs. All searches involved free-text searching and database specific MeSH subject headings (where appropriate), and were limited to ‘English Language’ only (S1 Appendix). We updated this search in July 2021 to ensure recent literature was captured.

Backwards and forwards citation-chasing was conducted on the included studies to identify any other relevant literature that may not have been captured by the search. In addition, study authors were contacted in order to identify any additional papers relating to RCTs included within the review.

### Study selection

Search results were imported to EndNote V9 [55] and duplicates removed manually, matching records on; 1) author and title; 2) author and year; and 3) title and year. We then ran records through EPPI-Reviewer 4 RCT classifier [56] to categorise the search results based on their likelihood of reporting on an RCT and transferred back to EndNote V9 for screening.

Title and abstract screening was conducted by KA and a second independent reviewer (KF, AG, MF, ET, VB) where studies were classified by EPPI-Reviewer 4 as ≥20% likelihood of employing an RCT, and by KA alone where studies were classified as <20% likelihood of employing an RCT [56]. Full-text screening was conducted by KA and a random 10% were screened by a second independent reviewer (KF and MF) to ensure inclusion/exclusion criteria were applied consistently across studies. In both instances, disagreements were resolved through discussion and/or consultation with a third reviewer (VB).

### Data extraction

Data were extracted by KA using a standardised data extraction form (see S1 Appendix) which was piloted prior to use. Extracted data included study details (authors, date, study design, country, primary aim, the proposed relationship between DVA, MH, and SU as described by authors), study sample (recruitment setting, sample characteristics such as number, age, gender, and ethnicity, and study inclusion/exclusion criteria), intervention and control group details (guided by the TIDIER checklist; [57]), data collected on DVA, MH, and/or SU (data collection time-points, measures, and results), data collected on child MH outcomes (data collection time-points, measures, and results), other outcomes assessed (outcomes and measures), and authors’ conclusions and recommendations for future research. Data from a random 10% of included studies were also extracted by a second independent reviewer (KF and AG) to ensure accuracy. Disagreements were resolved through discussion and/or consultation with a third reviewer (VB).

Data were sought from the articles included in the review including associated supplementary material containing information on DVA, MH, and/or SU and weblinks provided in text to any additional information on these outcomes.

### Quality appraisal

KA quality-appraised the studies using the Risk of Bias Tool 2 (RoB2) for RCTs [58] and cluster RCTs [59] and a random 10% were quality appraised by a second independent reviewer (VB, G.J.M-T, TF, CB). Disagreements were resolved through discussion.

Our review uses terms from the RoB2 to refer to study quality. The RoB2 assesses the risk of bias arising from the randomisation process, identification and recruitment of participants to cluster RCTs (in the case of cluster RCTs only), assignment to the intervention group, missing outcome data, measurement of the outcome, and selection of reported results [58, 59].

### Data analysis

The significant heterogeneity in intervention types, outcome measures and length of follow-up precluded meta-analysis so we conducted a synthesis without meta-analysis in line with SWIM guidelines [60].

Standardised mean differences (SMD) (i.e., Cohen’s d) and associated 95% confidence intervals (CIs) were calculated for each primary outcome of interest within each study using the information available. These were calculated between intervention and control groups at post-intervention (operationalised as the closest data-collection point following intervention delivery) and follow-up (operationalised as the latest possible timepoint following data collection at post-intervention) using the Campbell Collaboration Effect Size Calculator [61] and guidance from Borenstein et al. [62] for conversion of odds ratios and calculation of SMD variance, where applicable. The direction of SMDs and CIs were multiplied by -1 where appropriate. Where studies reported no significant differences between groups and provided no further data, SMDs were imputed as 0 and SMD variance was estimated using Borenstein et al. [62] formula using imputed SMD and reported sample size [63, 64]. Four studies did not provide adequate information to allow us to calculate SMD, 95% CIs, and determine the direction of the SMD for two or more outcomes at post-intervention [65–68] and four at follow-up [66–69]. For these studies, findings are reported narratively based on the authors report. One study did not present sufficiently detailed results in text or tables and therefore, study authors were contacted to request means and SDs at post-intervention [70].

Our primary outcomes included parental DVA, MH, and SU. Where there were multiple measures assessing the same outcome, a decision tree was followed to decide which data to synthesise, giving priority to; 1) measures collecting and presenting data on the time-point of interest (i.e., post-intervention or latest follow-up); 2) continuous outcomes; 3) imputed data; 4) analyses controlling for the most covariates. Where multiple measures met these criteria, or where only dichotomous outcomes were available, all were included within the analysis.

We reported findings narratively, grouping studies based on the combination of outcomes measured, as examining combined impacts was the primary aim of the review. We summarised studies using tables which highlighted key study characteristics. Harvest plots were used to illustrate the direction of effect and certainty of effect (i.e., 95% CI are both positive, cross zero, or are both negative) for DVA, MH, and SU outcomes within each study, the number of SMDs these categorisations were based on, and the combination of outcomes each study examined. We also used harvest plots to highlight studies that had combined impacts on two or more outcomes, categorising in terms of whether the effects for DVA, MH, or SU favoured the control (all SMDs favoured control), were mixed (some SMDs favoured control and some favoured intervention), or favoured the intervention (all SMDs favoured the intervention) and highlighting where two or more of DVA, MH, and/or SU outcomes demonstrated SMDs with positive CIs, CIs that crossed zero, or negative CIs. Harvest plots were used as they provide a useful way to organise/synthesise data about differential effects of complex interventions that may not be appropriate for meta-analyses [71].

### Patient and public involvement and engagement (PPIE)

Patient and public involvement and engagement (PPIE) of those with experience of DVA, MH, and/or SU, service providers, and commissioners was essential in informing the design and conduct of our review.

Our review focuses on clustering DVA, MH, and SU following calls from commissioners for help in finding better ways to prevent and respond to these issues. The scope of the review was further refined to focus on family focused interventions following direction from those with experience, who highlighted the intergenerational nature of these issues and the importance of working with parents and child when providing support; a view echoed by service providers and commissioners. In addition, almost all those with experience talked about the impact these experiences had on their children (who lived at home, or with whom they had regular contact).

Primary prevention approaches, which seek to intervene early to prevent DVA, MH, and SU later in life, tend to be predominately school based, child focused, and involve measuring changes in attitudes and beliefs rather than social, emotional, and behavioural outcomes (e.g., [72–74]). Therefore, we focused on other levels of prevention to capture family focused interventions that might measure direct impacts on DVA, MH, and SU. Engagement work with LA commissioners highlighted the need to define these preventive interventions as both secondary (targeting individuals/populations at risk of, or experiencing early signs of, a particular issue) and tertiary (preventing negative impacts associated with a particular issue) interventions [75, 76], and to expand the categorisation to include treatment interventions, in recognition of the fact that these interventions often have preventive elements and may be used by commissioners for preventive purposes. This addition considerably expanded the scope of the review, but ensured it was more useful to those who might seek to apply its findings.

Finally, PPIE helped to inform our interpretation and presentation of the results. Collaborators helped to shape how the findings were presented in study characteristics tables (e.g., highlighting the context in which the interventions are situated) and structure the discussion, where we highlight findings believed to be particularly important from a commissioner and service provider perspective.

## Results

**Fig 1:**
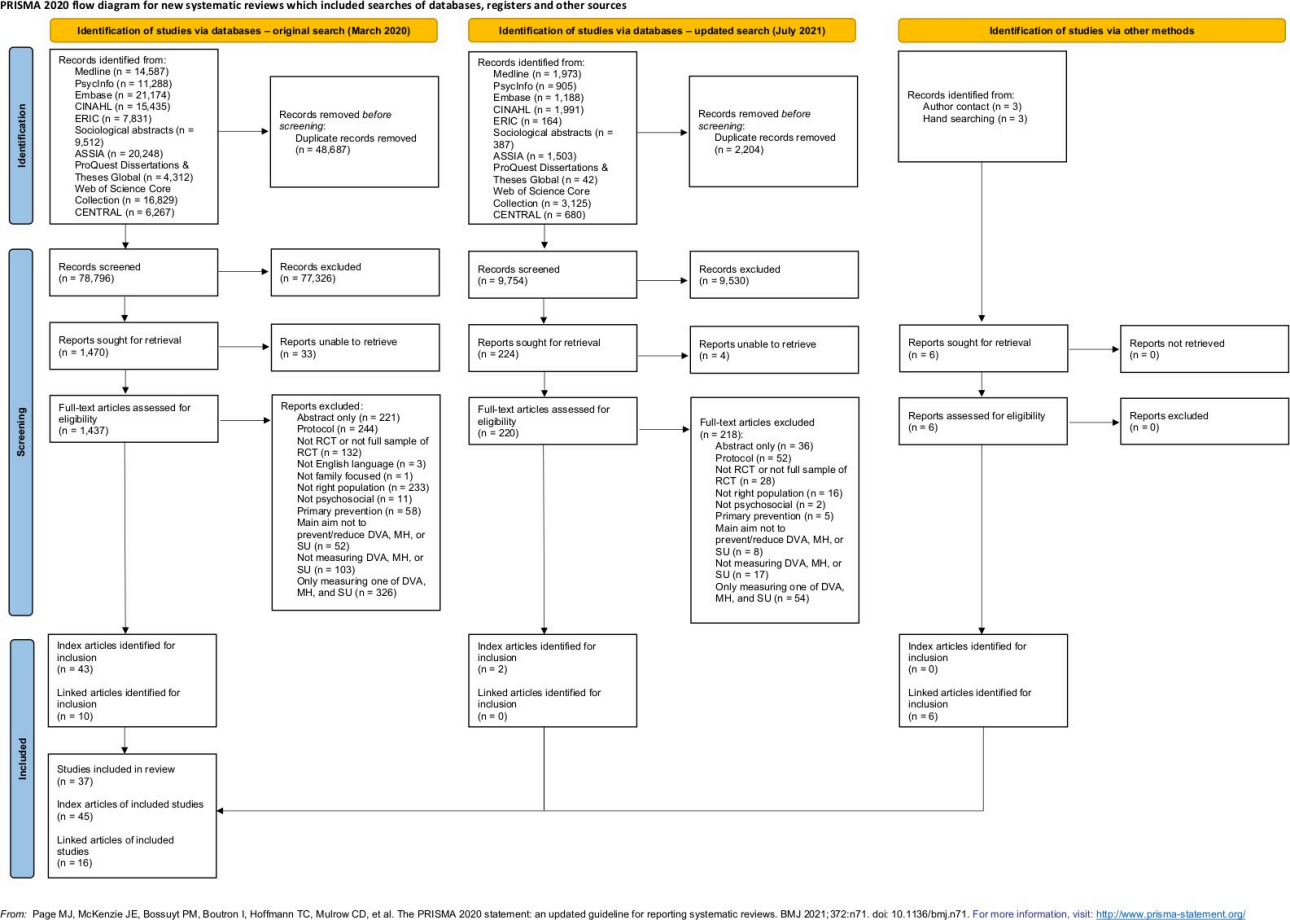
PRISMA flow diagram.

Our original search returned 127,483 results, reduced to 78,796 following de-duplication. In total, 1,470 results were screened on full text, which resulted in 43 index papers and 10 linked papers (i.e., papers linked to included index papers that contained additional information on child outcomes and/or parental DVA, MH, and SU) corresponding to 35 unique studies (Fig 1). A further two eligible studies were identified through an updated search, resulting in a total of 37 unique studies identified for inclusion (Fig 1). Six additional linked papers were identified through contacting the authors/hand searching. Where there are multiple papers associated with one study, we use the primary reference to refer to the study.

### Study characteristics

Study characteristics are summarised in Tables 1–4. All studies were published as peer-reviewed journal articles bar two PhD theses [77, 78]. Three studies employed a cluster RCT [79–81] instead of an RCT randomised at the individual level and six employed a pilot RCT [65, 82–86] as opposed to a full-sized RCT. The type of control group varied across studies with 15 employing an active control [65–68, 77, 79, 82–85, 87–91], 11 a care as usual control [64, 69, 70, 80, 81, 92–97], six a minimal care control [86, 98–102], and three employing both active and usual care controls [63, 78, 103]. Two studies provided no information on the nature of the control group [104, 105]. Most studies were conducted in the U.S. [63, 64, 66–68, 78, 79, 82–84, 86–89, 91, 92, 95, 98–103, 105], with the remaining studies conducted in Australia [81], Canada [77], South Africa [80], New Zealand [106], China [93], UK [69, 94], Iran [70], Columbia [97], or an undisclosed country most likely to be the U.S. based on author affiliations [65, 85, 90, 104]. Family focused interventions worked with the mother [63, 66, 69, 70, 78–82, 85, 86, 88, 91–94, 98–103, 105, 106], mother and father [65, 77, 83, 87], or parents [68, 97] with the view that this would indirectly impact the child. However, three studies worked directly with the mother and child [64, 89, 104], two with the mother, father, and child [84, 90], and one with a parent and child [95]. No studies worked solely with the child. Studies represented a range of different ethnic groups and, even where participants weren’t specifically targeted due to low socio-economic status (SES), demographic data indicated study populations were experiencing above average levels of low SES (see S1 Appendix).

**Table 1:** Study characteristics for studies measuring DVA and MH.

Study characteristics for studies measuring DVA and MH									
Study	Context	Target population	Recruitment setting(s)	Level of prevention	Intervention form and function	Intervention duration and setting	Control(s)	Relationship between DVA/MH/SU	DVA/MH/SU outcomes measured*
**4. El-Mohandes et al. (2008)** Linked: Kiely et al. (2010); Kiely et al. (2011) (reference number: [92])	District of Columbia, U.S. 100% African American	African American pregnant women (aged 18+, ≤28 weeks gestation) living in district of Columbia who reported one or more of the following: active smoking, environmental tobacco smoke exposure, depression, and/or IPV (physical/sexual violence or scared of current partner). **Exclusion criteria:** completed pregnancy before baseline interview.	Community based prenatal clinics.	Treatment	**Multicomponent** intervention (n = 452) aiming to reduce postpartum risk factors including depression and DVA. Mothers received tailored support from master’s level trained counsellors to suit their needs; for those smoking or experiencing environmental smoke exposure they received an intervention to promote smoking cessation/reduction and environmental smoke avoidance, for those experiencing depression they received an adapted CBT intervention, and for those experiencing IPV they received individualised counselling sessions utilising empowerment theory (adapted from Parker-McFarlane).	Delivered in clinic over a period of at least 12 weeks (4–8 sessions prenatally and 2 optional sessions postpartum, frequency not stated); hosted within a community prenatal clinic.	Usual care control (n = 461). No further information provided.	**Authors describe as uni-directional**; experiencing IPV (DVA) is associated with an increased risk of depression, PTSD (MH), alcohol, and illicit drug use (SU). **Intervention treats as co-occurring**; provide separate support for DVA and MH using separate approaches.	**Post-intervention (10.3 weeks postpartum):** **DVA** = Mothers’ physical assault and sexual coercion victimisation measured using CTS. **MH** = Mothers’ depression measured using HSCL. **No follow-up.**
**16. Nagle (2002)** (reference number: [78])	Louisiana, U.S. 54% African American	First-time pregnant women (<28 weeks gestation) with low income (below 133% federal poverty level). **Exclusion criteria:** None stated.	Various; public health units and referrals to nurses from other services (e.g., schools, doctors, community resources).	Tertiary	**Home visiting supplement** (n = 135) called ‘Nurse Home Visiting Plus’ provided in addition to NFP home visiting intervention aiming to strengthen the mother-child relationship by supporting the mother in their development and parenting and helping them understand the needs and development of their children. Mothers received home visits from a home visiting nurse who was part of a larger team which included a team supervisor, eight home visiting nurses, and a mental health professional with expertise in infant mental health. The mental health professional supported the team and acted as a mental health provider to mothers.	Delivered in home setting over a period of 2+ years (number of sessions not stated, sessions every other week); hosted within home visiting services.	Two groups^1^: **1)** Usual care control (n = 116) who received existing public health services. **2)** Active control (n = 106) who received normal home visiting (NFP).	**Author describes as bi-directional**; DVA is likely to increase the risk of depression (MH) and likewise, depression (MH) is likely to increase the risk of DVA. **Intervention treats as co-occurring**; provide separate support for MH and DVA using separate approaches.	**Mid-treatment (6–8 months)**^**2**^: **DVA** = mothers’ physical and non-physical victimisation and perpetration related to current partner and ex-partner measured using the Partner Violence Interview. **MH** = mothers’ depression measured using the BDI. **No follow-up.**
**29. Sullivan et al. (2002)** (reference number: [104])	Midsize urban city, U.S. (state not stated) Mothers 49% Non-Hispanic White; children 44% African American	Mothers who have experienced physical violence from intimate partner or ex-partner in the last four months and have at least one child aged between 7–11 years who is living with them and interested in participating in the study. **Exclusion criteria:** None stated.	Various; following exit from DVA shelter or when obtaining services from community-based service or state Social Services department.	**Tertiary**/ secondary/ treatment	**Advocacy** intervention (n = 45) aiming to improve self-competence of children exposed to DVA, improve mothers’ psychological well-being, and protect against continued DVA. Mothers and children received family tailored strengths-based advocacy intervention from paraprofessionals (female undergraduates). This involved assessing mothers’ and children’s needs and goals and helping mothers to access and utilise community-based support in terms of legal assistance, housing, employment, education, childcare, social support etc. and helping children access recreational activities, supporting them with schoolwork, and/or obtaining material goods. As part of the intervention, children also attended a support and education group which was run by five group leaders who had experience working with children. At the end of the intervention, there was a focus on transferring advocacy-based skills to the mother.	The advocacy part of the intervention was delivered in the home setting/over the telephone over 16 weeks (minimum 36 sessions, at least twice a week) and the children’s support and education group in a community-based setting over 10 weeks (do not state number/frequency of sessions); hosted within community IPV services.	No information given (n = 33).	**Authors describe as uni-directional (theoretical link)**; women that experience DVA are at risk of experiencing high levels of psychological distress (including anxiety and depression; MH) due to unpredictable and inconsistent violence they experience. **Intervention treats as bi-directional**; support from advocate is hypothesised to help improve MH and protect against DVA.	**Post-intervention:** **DVA** = mothers’ emotional and physical victimisation measured using a combination of the shortened version of the Index of Psychological Abuse, modified version of CTS, and 12-item scale to assess injury. **MH** = mothers’ depression measured using the CES-D. **Follow-up** (4 months post-intervention): Same as above.
**30. Taft et al. (2011)** (reference number: [81])	NW Melbourne, Australia Ethnicity not reported	Mothers (aged 16+) who were pregnant or had one child aged ≤ 5 and were identified as psychologically distressed (symptoms of depression, anxiety, or frequent attendance taken as an indicator of being at risk of IPV) or had disclosed IPV. **Exclusion criteria:** Serious MH and not taking medication, English not adequate to provide informed consent (unless spoke Vietnamese).**Exclusion criteria:** Serious MH and not taking medication, English not adequate to provide informed consent (unless spoke Vietnamese).	GP practices and Maternal and Child Health Clinics (MCHs).	**Secondary**/ **treatment**/ tertiary	**Advocacy** intervention (n = 113) called ‘MOtherS’ Advocates In the Community’ (MOSAIC) aiming to reduce DVA and/or depression among mothers, strengthen their health and well-being, and strengthen mother-child bond. Mothers received home visits from individually matched paraprofessionals (mentor mothers) who offered advocacy-based support, parenting support and general be-friending in addition to normal clinician care.	Delivered in home setting over a period of 12 months (48 sessions delivered weekly); hosted within primary care.	Usual care control (n = 61) which involved receiving a resource card containing details of family violence services.	**Authors describe as uni-directional**; maternal depression (MH) can be a common consequence of IPV (DVA). **Intervention treats as bi-directional;** support from advocate is designed to provide MH support at same time as advocacy for DVA.	**Post-intervention:** **DVA** = mothers’ physical and emotional victimisation and harassment measured using the CAS. **MH** = mothers’ depression and general MH measured using the EPDS and SF-36 MH subscale, respectively. **No follow-up.**
**31. Tiwari et al. (2005)** (reference number: [93])	Hong Kong, China Ethnicity not reported	Chinese pregnant women (aged 18+, <30 weeks gestation) identified as experiencing DVA by an intimate partner during their first antenatal appointment. **Exclusion criteria:** None mentioned.	Antenatal clinics.	Treatment	**Empowerment** intervention (n = 55) aiming to reduce IPV. Pregnant women received an empowerment-based intervention delivered by a research assistant (who was a trained midwife). The intervention involved giving pregnant women advice on safety, choice making, and problem solving (based on Parker et al.’s empowerment protocol) and also included a component on empathetic understanding (based on Roger’s client-centred therapy) to help increase women’s positive feelings about themselves. At the end of the intervention women received a leaflet covering the information discussed.	Delivered in clinic as a one-off 30-minute session; hosted within antenatal services.	Usual care control (n = 55) which involved receiving wallet sized card containing information on community resources for DVA.	**Authors describe as uni-directional**; IPV (DVA) may have a detrimental impact on self-esteem (MH). **Intervention treats as bi-directional**; DVA and MH addressed concurrently within the same one-off intervention.	**Post-intervention (6 weeks post-delivery):** **DVA** = mothers’ psychological, physical, and sexual victimisation measured by the CTS. **MH** = mothers’ depression and general MH measured by the EPDS and SF-36 MH subscale, respectively. **No follow-up.**
**35. Zlotnick et al. (2011)** (reference number: [86])	Rhode Island, U.S. 42.6% Hispanic	Low income, pregnant women (aged 18–40 years) attending their prenatal care visit who were deemed at risk of MH due to screening positive for experiencing IPV in the past year. **Exclusion criteria:** meet criteria for current affective disorders, PTSD, or SU on SCID-NP, attending clinic with male partner, currently receiving treatment for MH, only one instance of very minor abuse.	Primary care clinics and private OBGYN clinic.	Secondary	**Therapy** (interpersonal psychotherapy) intervention (n = 28) aiming to prevent/reduce PTSD and depression in low-income women who have experienced IPV in the past year. Pregnant women received an interpersonal therapy-based intervention delivered by two study interventionists (trained to deliver scripted intervention). Involved helping women with changing expectations around interpersonal relationships, building and improving their social network and helping them with their transition to motherhood. Over multiple sessions discussed healthy relationships, developing safety plans, developing good support network, the consequences of abuse including risks to MH and SU and what this might look like, support networks, and goal setting. It was also informed by empowerment and stabilisation-based models recommended for IPV.	Delivered in undisclosed setting over a period of less than six weeks (4 sessions prenatally, 1 session postnatally, frequency not stated); not clear who it was hosted by.	Minimal care control (n = 26) involving usual care plus educational material and list of resources for IPV.	**Authors describe as bi-directional**; PTSD/depression (MH) are possible consequences of IPV (DVA). Depression/PTSD (MH) may serve to sustain vulnerability of woman to abuse. **Intervention treats as bi-directional**; target women’s social support for interrupting relationship between DVA and MH (and DVA alone), discuss MH as consequence of DVA, target MH with view this will encourage women to seek help from other services for DVA and establish safety.	**Post-intervention:** **DVA** = women’s physical, psychological, and sexual victimisation measured using CTS2. **MH** = women’s depression measured using LIFE and EPDS. Women’s PTSD measured using LIFE and Davidson Trauma Scale. **Follow-up** (10 weeks post-intervention): Same as above.
**UPDATE** **36. Dinmohammadi et al. (2021)** (reference number: [70])	Zanjan, Iran Ethnicity not reported	Pregnant women (aged 18+, ≤27 weeks gestation) who were married, living with their partner, and were experiencing minor/medium levels of DVA. Pregnant women also had to own a cell phone and not be participating in any other classes or counselling. **Exclusion criteria:** psychological illness, SU (pregnant woman or partner), absent for more than one counselling session.	Health care setting—government delivered childbirth preparation class.	**Treatment**	**Therapy** (solution-focused) intervention (n = 45) aiming to reduce DVA and improve quality of life. Pregnant women received individual solution focused counselling sessions delivered by a researcher. The sessions involved familiarising women with the concept of the solution-focused approach and quality of life, learning how to best interpret events, thinking about opportunities when living as a couple, recognising destructive behaviour patterns, and developing new thoughts and behaviours.	Delivered in health care setting over a period of 6 weeks (6 sessions, once per week); hosted within government delivered childbirth preparation classes.	Usual care control (n = 45) who were offered the intervention after the study was complete. No other information given.	**Authors describe as uni-directional;** DVA may lead to problems with MH. **Intervention treats as bi-directional;** addressing both DVA and MH through solution-focused therapy.	**Post-intervention (6 weeks post-intervention):** **DVA =** women’s physical, psychological, sexual violence and injury-related victimisation using CTS-2. **MH =** women’s general MH using mental health subscale of SF-36. **No follow-up.**
**UPDATE** **37a. Skar et al. (2021)**^3^ (reference number: [97])	Chocó Department, Columbia Ethnicity not reported	Parents of children aged between 3–4 years who were attending one of six child centres and were receiving health services subsidised by the government (due to low-income). **Exclusion criteria:** did not take part in the programme or sent someone else to complete outcome measures.	Social services child centres run by Instituto Colombiano de Bienestar Familiar (ICBF).	**Tertiary**	**Parenting intervention** (n = 59) called ‘International Child Development Programme (ICDP)’ aiming to promote good parenting and strengthen child-parent relationship by influencing parent attitudes, increasing parent self-confidence, and promoting empathy and sensitivity to child’s needs. Parents received ICDP sessions delivered by trained ICDP facilitators. These sessions involved group discussions, role play, home practice (i.e., activities in the home setting between sessions), and reflection on home practice focusing on emotions, communication and regulation related to parent-child interactions.	Delivered in social services child centres over an undisclosed period of time (12 sessions, frequency not stated); hosted within social services.	Usual care control (n = 51) who had access to usual health, nutrition, and educational facilities at the child centre they were attending.	**Authors do not state**; do not discuss potential relationship between DVA and MH but do explore this in the analysis suggesting those experiencing DVA are more likely to experience MH. **Intervention treats as co-occurring;** attempting to address DVA and MH through common risk factor however no recognition that these issues cluster in this context.	**Post-intervention (6 months post baseline):** **DVA =** parents’ physical and psychological victimisation and perpetration using HITS. **MH =** parents’ general MH using SSQ. **No follow-up.**
**UPDATE** **37b. Skar et al. (2021)**^3^ (reference number: [97])	Chocó Department, Columbia Ethnicity not reported	Parents of children aged between 3–4 years who were attending one of six child centres and were receiving health services subsidised by the government (due to low-income). **Exclusion criteria:** did not take part in the programme or sent someone else to complete measures.	Social services child centres run by Instituto Colombiano de Bienestar Familiar (ICBF).	**Tertiary**	**Parenting supplement** (n = 66) called ‘International Child Development Programme (ICDP)’ plus ‘violence curriculum’ (VC) aiming to better prevent violence in the home. Parents receive ICDP as described above (but shortened in duration) and receive additional sessions focusing on violence delivered by trained ICDP facilitators. These additional sessions involve training on child development, violence, legislation and policy, child protection systems, and their role in protecting children. Parents also develop protective strategies and monitoring tools to enable them to do this.	Delivered in social services child centres over an undisclosed period of time (12 sessions: 6 sessions ICDP and 6 sessions VC, frequency not stated); hosted withing social services.	**Usual care control** (n = 51) who had access to usual health, nutrition, and educational facilities at the child centre they were attending.	**Authors do not state**; do not discuss potential relationship between DVA and MH but do explore this in the analysis suggesting those experiencing DVA are more likely to experience MH. **Intervention treats as uni-directional DVA focused;** primary aim is to reduce DVA to prevent negative impact on children. Improved MH likely to be an additional benefit.	**Post-intervention (6 months post baseline):** **DVA =** parents’ physical and psychological victimisation and perpetration using HITS. **MH =** parents’ general MH using SSQ.

**NB.** Green coloured cells indicate studies that have had, or report to have, combined positive impacts on two or more outcomes. 1 for outcome data we compare intervention to the usual care control. 2 Although there were multiple follow-up timepoints, DVA and MH outcomes were only measured at baseline and mid-treatment. We treat this mid-treatment point as post-intervention within the analysis. ^3^ Skar et al. (2021) conducted a three-arm RCT examining the effectiveness of a parenting intervention (intervention group 1) and a parenting intervention plus parenting intervention supplement (intervention group 2) as compared to a usual care control group. Therefore, there are two separate entries for this study, one with the parenting intervention as the intervention group and one with the parenting intervention supplement as the intervention group. BDI = Beck Depression Inventory; CAS = Composite Abuse Scale; CBT = Cognitive Behavioural Therapy; CES-D = Center for Epidemiological Studies Depression Scale; CTS = Conflict Tactics Scale; DVA = Domestic Violence; EPDS = Edinburgh Postnatal Depression Scale; HITS = Hurt, Insult, Threaten, Scream; HSCL = Hopkins Symptom Checklist; IPV = Intimate Partner Violence; LIFE = Longitudinal Interval Follow-up Examination; MH = Mental ill-health; NFP = Nurse Family Partnership; PTSD = Post Traumatic Stress Disorder; SF = Short Form; SSQ = Shona Symptom Questionnaire; SU = Substance Misuse.

**Table 2:** Study characteristics for studies measuring DVA and SU.

Study characteristics for studies measuring DVA and SU									
Study	Context	Target population	Recruitment setting(s)	Level of prevention	Intervention form and function	Intervention duration and setting	Control(s)	Relationship between DVA/MH/SU	DVA/MH/SU outcomes measured*
**10. Jacobs et al. (2016)** Hand linked: Jacobs et al. (2015) (reference number: [98, 107])	Massachusetts, U.S. 36.8% Non-Hispanic White	Young, first-time parents (aged 16–21 years) who are new to the home visiting intervention and spoke either English or Spanish. **Exclusion criteria:** None stated.	Established home visiting sites.	Tertiary	**Home visiting** intervention (n = 433) called ‘Healthy Families Massachusetts’ aiming to prevent child abuse and neglect, help children achieve optimal growth and development, encourage educational attainment and enhance job and life skills among parents, prevent repeat pregnancies during adolescence, and promote health and well-being of parents. Mothers received home visits from paraprofessionals who provided families with tailored support that involved goal setting, curriculum-based activities, routine health and development meetings, and referral to other community-based services (for DVA/MH/SU).	Delivered in home setting over a period of 2–3+ years (up until child’s third birthday; number of sessions varied depending on family need average 24, tended to be biweekly during pregnancy, weekly in the first 6 months postpartum, reducing as families progressed through the intervention); hosted within established home visiting sites.	Minimal care control (n = 271) were provided with information about child development and referred to other services.	**Authors do not state**; DVA and MH both risk factors related to young parenthood. **Intervention treats as co-occurring**; DVA and SU addressed using referral to other DVA and SU community services.	**Post-intervention (24 months post enrolment):** **DVA** = mothers’ physical, psychological, sexual coercion and injury related perpetration and victimisation measured using CTS2 short form. **SU** = mothers’ alcohol, drug, and marijuana use measured using YRBSS. **No follow-up.**
**12. Lam et al. (2009)** (reference number: [83])	U.S. (state not stated) Fathers 63.3% White; Mothers 66.6% White	Fathers (18+ years) who had diagnosed alcohol abuse / dependence and were voluntarily entering outpatient treatment for this. Fathers had to have an intimate female partner they were married to (for at least 1 year) or cohabiting with (for at least 2 years) who did not meet criteria for SU abuse / dependence. They also had to have at least one child (aged between 8–12 years) who was in their care and living at home. **Exclusion criteria:** None stated.	Outpatient treatment clinics for alcohol use disorder.	Tertiary	**Multicomponent** intervention (n = 10) referred to as ‘PSBCT’ aiming to improve parenting skills in parents where the father is alcohol dependent and therefore, at risk of DVA. Fathers and their female intimate partners received parent skills training and BCT from master’s level therapists (with expertise in BCT and coping skills therapy for SU) in addition to the cognitive-behavioural coping skills therapy for alcohol treatment the father was already receiving as part of outpatient SU clinic treatment. The parent skills training was based on Forehand’s program and the BCT involved urine screens, homework, improving on communication and problem-solving skills, and encouraging abstinence. 2) Active control IBT (n = 10) involved 12 coping skills sessions developed from modified CBT for alcoholism.	Delivered in clinic over a period of 12 weeks (24 sessions, twice per week); hosted within an outpatient treatment clinic for alcohol abuse / dependence.	Two control groups. Fathers in both groups received 12 sessions of standard individual CBT in addition to^1^: 1) Active control BCT (n = 10) involved 12 sessions and included homework, urine screens, improving communication and problem-solving skills, and encouraging abstinence.	**Authors describe as uni-directional**; SU increased the risk of partner violence (DVA) which may heighten child maltreatment risk. Those reporting partner violence (DVA) often report using substances (SU) at the time of the physical violence. **Intervention treats as uni-directional -SU focused**; target fathers’ alcohol use in the hope this will also lead to reductions in DVA.	**Post-intervention:** **DVA** = Male to female and female to male physical violence victimisation / perpetration measured using the TLFB-SV. **SU** = Fathers’ alcohol use measured using TLFB. **Follow-up (12 months post-intervention):** Same as above.
**13. LeCroy et al. (2011)** (reference number: [99])	Arizona, U.S. 59.8% Hispanic	Prenatal/new parents deemed at risk of child maltreatment (scoring >25 on Kempe Family Checklist). **Exclusion criteria:** None stated.	Home visiting sites.	Tertiary	**Home visiting** intervention (n = 98) called ‘Healthy Families Arizona’ aiming to promote positive parenting, enhance child health and development, and prevent child abuse and neglect. Mothers received home visits from female home visitors who provided parents with support in terms of helping with life circumstances, personal problems, parenting, establishing a safe and medical home for the child, and transitioning to parenthood, reviewing the child’s developmental progress, providing emotional support and mobilising services to address DVA, MH, and SU.	Delivered in home setting, no information given about the duration, number of sessions or frequency of sessions; hosted within home visiting sites.	Minimal care control (n = 97) received information on their child’s developmental progress and were referred to other services as needed.	**Authors do not state**; DVA often co-occurs with child maltreatment. **Intervention treats as co-occurring**; addressed using referral to other community services.	**Post-intervention (child 12 months of age)**^**2**^: **DVA** = mothers’ physical victimisation measured using own index based on CTS2. **SU** = mothers’ alcohol use measured using own questions. **No follow-up.**
**25. Stover (2015)** (reference number: [65])	U.S. (state not stated) 52% African American	Fathers experiencing co-occurring SU (meeting DSM-IV criteria for alcohol, cocaine or marijuana use and using within last 30 days) and DVA (reported physical violence in intimate relationship within last 90 days), who were the biological father of at least one child under the age of 10 who was living with them or they had visitation rights to. **Exclusion criteria:** Fathers with suicidal or psychotic symptoms, history of bipolar or psychotic disorder, demonstrated sig coercive control, history of severe violence, or who had female co-parent who feared them or did not want child to participate.	Referrals from criminal justice facilities.	Treatment / Tertiary	**Therapy** (individual and dyadic) intervention (n = 9) called ‘Fathers for Change’ aiming to reduce fathers’ co-occurring SU and IPV, promote co-parenting, and promote healthy father-child relationships. The intervention was delivered to fathers, mothers, and children in three separate parts all of which were delivered by trained therapists. The first part involved individual therapy sessions with the father. This was followed by co-parenting sessions for both the father and mother (provided the mother was comfortable to attend and the father had made sufficient progress in individual sessions). The final stage involved restorative parenting sessions delivered to the father and child. The intervention was grounded in attachment, family systems, and cognitive behavioural theory and focused on encouraging abstinence from SU and DVA, parents communication skills, parenting within the father-child relationship, and encouraging fathers to develop competence and value within their parenting role in order to provide motivation for change in terms of DVA and SU.	Delivered face-to-face in undisclosed setting over a period of approximately 4 months (6–8 individual father sessions, 6 dyadic co-parenting sessions, 4–6 restorative parenting sessions, frequency not specified); hosted within a parenting program.	Active control (n = 9) fathers received individual drug counselling delivered over a period of 16 weeks (number of sessions and frequency not specified).	**Author describes as uni-directional**; negative feelings (MH) are likely to lead to IPV (DVA) and SU as a means to control these feelings, IPV (DVA) is more likely to be present if SU is present and when they co-occur, they are more likely to lead to negative parenting behaviours/less positive parenting behaviours. **Intervention treats as bi-directional**; targets both concurrently recognising the association between them.	**Post-intervention:** **DVA** = fathers’ physical perpetration and victimisation measured using the CTS2. **SU** = fathers’ substance use measured using TLFB. **No follow-up.**^3^

**NB**. Green coloured cells indicate studies that have had, or report to have, combined positive impacts on two or more outcomes. ^1^ for outcome data we compare intervention to active control as the intervention has been designed to outperform this in terms of key outcomes. ^2^ Intervention duration was not specified and therefore, the last available timepoint was taken as ‘post-intervention’. ^3^ Authors conduct follow-up at 3 months post-intervention however do not measure both DVA and SU at this time point (only measure DVA) therefore, unable to examine combined impacts. BCT = Behavioural Couples Therapy; CTS2 = Conflict Tactics Scale; DVA = Domestic violence; SU = Substance misuse; IBT = Individual Behavioural Therapy; TLFB = Timeline Follow Back Interview; TLFB-SV = Timeline Follow Back Interview-Spousal Violence; YRBSS = Youth Risk Behaviour Surveillance System.

**Table 3:** Study characteristics for studies measuring MH and SU.

Study characteristics for studies measuring MH and SU									
Study	Context	Population	Recruitment setting(s)	Level of prevention	Intervention form and function	Intervention duration and setting	Control(s)	Relationship between DVA/MH/SU	DVA/MH/SU outcomes measured*
**1. Cupples et al. (2010)** (reference number: [94])	Northern Ireland, UK Ethnicity not reported	First-time mothers (aged 16–30 years, >20 weeks gestation), with no comorbidities requiring hospital care, attending their first antenatal visit and living in socio-economically deprived areas. **Exclusion criteria:** previous miscarriage, previous pregnancy, non-English speaking, moved out of area, declined to confirm details.	Hospitals during mothers’ first antenatal visit.	Secondary / tertiary	**Home visiting intervention** (n = 172) aiming to help with infant development and maternal health (including MH + SU). Delivered to mothers by peer-mentors (who had been matched to mothers in terms of age and locality) who provided them with health-related information in terms of general support, infant feeding/breast feeding, maternal diet/healthy eating, stress/relaxation, local or hospital services, lifestyle factors (SU), welfare / benefits / entitlements, self-esteem and confidence, and immunisations.	Delivered in home/remotely over a period of 17 months (until child 1 year of age; mother decides number of sessions, ideally twice monthly during pregnancy and once monthly during child’s first year of life); hosted within community/midwifery services.	Usual care control (n = 171) received usual care which involved frequent healthcare contacts during pregnancy / postpartum.	**Authors do not state**; MH and SU both elements of maternal health. **Intervention treats as co-occurring**; separate advice on each.	**Post- intervention:** **MH** = mothers’ general MH measured using SF-36 MH subscale. **SU** = mothers’ drug use and alcohol use measured using a self-report lifestyle questionnaire.^1^ **No follow-up.**
**6. Fleming et al. (2008)**; Wilton et al. (2009) (reference number: [100, 108])	Wisconsin, U.S. 81% White	Postpartum mothers (aged 18+, day 45 postpartum) who were attending a postpartum appointment with an obstetrician or advanced practice nurse and screened positive for high-risk alcohol use. **Exclusion criteria:** None stated.	Obstetrical practice clinics during scheduled appointments for postpartum care.	Secondary / treatment	**Brief intervention** (n = 122) aiming to reduce postpartum alcohol use. Mothers received a brief intervention and reinforcement session delivered by a clinic nurse or obstetrician and two follow-up phone calls after each session from an interventionist. The face-to-face sessions involved mothers working through a workbook containing psychoeducation around alcohol use, a worksheet on drinking cues, a drinking agreement, and drinking diary cards. In between these sessions, mothers did homework around drinking situations and cues and completed the drinking diary cards. The phone calls provided continued support, discussing challenges faced and reinforcing key messages. The intervention was guided by CBT and motivational interviewing principles.	Delivered in clinic/remotely over a period of 8 weeks (brief intervention and reinforcement session delivered one month apart, follow-up phone calls delivered two weeks after each session); hosted within community-based obstetrical practice clinic.	Minimal care control (n = 113) received a general health booklet and were encouraged to address any health concerns in the ‘usual manner’.	**Authors describe as uni-directional**; postpartum alcohol use (SU) can increase risk of depression (MH) and IPV (DVA), all three of which are associated with increased risk of child behaviour problems. **Intervention treats as uni-directional–SU focused**; target SU with the view this will impact MH.	**Post-intervention (6 months post baseline):** **MH** = mothers’ depression measured using EPDS. **SU** = mothers’ alcohol use measured using TLFB. **No follow-up.**
**7. Grigg (1994)** (reference number: [77])	Canada 97% White	Alcohol dependent fathers who had consumed alcohol within the last three months, lived with the mother of their child (who did not have dependency problems) in either common law or marriage for at least one year, reported martial distress with mother of their child but wanted to remain in the relationship, and had at least one child (biological or non-biological) who was living with them or they were in regular contact with. **Exclusion criteria**: Fathers’ alcohol problems not severe enough, mothers’ alcohol use too high, martial distress negligible, severe psychiatric disturbance.	Various; SU services (rehabilitation facilities or community outpatient clinics), referrals from wider network, or self-referrals.	Treatment	**Therapy intervention** (n = 77) called ‘Experiential Systemic Therapy’ (ExST) aiming to reduce father’s alcohol use and prevent relapse. Fathers and mothers (ExST-couple; n = 39) or fathers alone (ExST-individual; n = 38) received ExST delivered by trained therapists (with experience working with SU). ExST focused predominately on relationships–intrapersonal, interpersonal, and relationships within the wider context–and how identities are formed, revised, and reformed based on these recurrent relationships. Three interlocking dimensions to the therapy included; 1) the experiential (need an experience), 2) the symbolic (symbols are used within therapy and therapy itself is seen as symbolic), and 3) the systemic (couple, family, and wider). ExST was underpinned by systemic, ecological systems theory and attachment theory.	Delivered in clinic over a maximum period of 20 weeks (12.8 sessions average for ExST-individual, 15 sessions max for ExST-couple, no information on frequency); hosted within SU clinic.	Active control (n = 38) fathers received ‘Supportive Feedback Therapy’. Involved weekly or bi-weekly sessions with a therapist to help father self-monitor their behaviour using visual monitoring charts. Designed to contrast with the intervention on all key elements being structured and predictable, uniform, low intensity, learning from present and past events, and cognitive-behavioural.	**Author describes as co-occurring:** verbal/physical abuse (DVA) often occurs when alcohol abuse is present (SU). **Intervention treats as uni-directional–SU focused**; targeting relationships to reduce SU and believe this will also then impact MH.	**Post-intervention (15 weeks post baseline for control, 20 weeks post baseline for intervention):** **MH** = fathers’ and mothers’ depression, anxiety, OCD, and general MH measured using BDI and SCL-90-R subscales. **SU** = fathers’ alcohol use measured using ADD. **No follow-up.**^2^
**11. Jones et al. (2011)** (reference number: [87])	Baltimore, U.S. 50.9% White	Opioid-dependent pregnant women (aged 18+, <30 weeks gestation) who had an intimate male partner (aged 18+) they were seeing at least three times a week who was also using opioids (not incarcerated and no evidence of physical IPV). **Exclusion criteria:** Pregnant women or partners with serious medical conditions or psychiatric illnesses.	Centre for addiction and pregnancy, located at a medical centre.	Treatment	**Multicomponent intervention** (n = 45) called ‘Helping Other Partners Excel’ (HOPE) aiming to engage and retain opioid-using pregnant women’s intimate male partners in drug treatment and stop their use of substances. Pregnant women and their intimate male partners received different elements of the intervention, all of which were delivered by counsellors who were MH professionals. Part 1 = motivational enhancement therapy (MET) and case management/proactive counselling delivered to intimate male partners, encouraging them to seek and enter treatment for drug use by rapport building, building self-efficacy to change and reinforcing positive behaviour change. Part 2 = couple’s pregnancy education and counselling delivered to pregnant women and their intimate male partners, providing psychoeducation on the effects of drug use on themselves and child development and education around pregnancy stages (and the support the male partner can provide), child development, and parenting skills. Part 3 = contingency management, providing male partners with monetary incentives to abstain from opioid and cocaine use (available throughout).	Delivered in clinic over a period of 22 weeks (Part 1 = weeks 1–6, 6 sessions, weekly; Part 2 = weeks 7–18, 12 sessions, weekly; Part 3 = weeks 1–22, 44 opportunities, twice weekly); hosted within community SU services.	Active control (n = 17) received standard care which involved a men’s support group delivered by counsellors over a period of 22 weeks (22 sessions, weekly). Also provided help with contacting treatment programs, arranging an intake appointment, and couples counselling where requested.	**Authors do not state.** **Intervention treats as uni-directional–SU focused;** targeting SU with the view this will impact MH as well. **SU** = male partners’ alcohol, heroin, cocaine, and general drug use measured using ASI, urine toxicology and risk assessment battery.	**Post-intervention (28 weeks post randomisation):** **MH** = male partners’ depression measured using the BDI-II. **No follow-up.**
**14. Luthar et al. (2007)** (reference number: [88])	New Haven, CT, U.S. 43.3% African American	Heroin addicted mothers with children under 16 years of age who reported problems with parenting. **Exclusion criteria:** cognitive deficits, psychotic thought processes, suicidality, homicidally.	Methadone clinics.	Tertiary	**Therapy intervention** (n = 60) called ‘Relational Psychotherapy Mothers’ Group’ (RPMG) aiming to promote optimal parenting in heroin addicted mothers. Mothers received group-based relational psychotherapy (groups of 3–8 mothers) delivered by trained therapists with experience working with families and addiction related issues. Therapy was manualised and semi-structured; first half focused on mothers own functioning and coping with anger, depression, and using guilt as a constructive mechanism for change in parenting; second half focused on positive parenting practices. Throughout therapy was underpinned by insight-orientated therapy, had an interpersonal and relational focus, provided non-judgemental supportive environment, involved role plays and encouraging mothers to explore own parenting practices to discover optimal strategies, and tackled isolation by providing intervention in group format. Mothers also received standard methadone treatment (counselling groups and meetings with case managers).	Delivered in clinic over a period of 24 weeks (24 sessions, weekly); standard methadone treatment delivered in clinic, duration not specified (number of sessions not specified, weekly); hosted within methadone clinics.	Active control (n = 67) mothers received recovery training delivered by professional clinicians with experience working with SU. Involved focus on process of addiction and recovery and relapse prevention. Do not specify duration, number of sessions or frequency. Also received standard methadone treatment (same as intervention).	**Authors describe as co-occurring**; mothers with SU show increased depression and anxiety (MH). MH can lead to problems with parenting. Intervening with MH might help reduce SU. **Intervention treats as uni-directional–MH focused**; intervene with MH to also help reduce SU.	**Post-intervention:** **MH** = mothers’ depression measured by BDI. **SU** = Mothers’ opiate and cocaine use measured using urine toxicology screens. **Follow-up (6 months post-intervention):** Same as above.
**15a. McWhirter (2011)**^**3**^ (reference number: [89])	Southwestern metropolitan area, U.S. 47% White	Mothers (and their children) who have experienced IPV within last year, reported their child being present for at least one incident of IPV in last year, and were currently residing in a temporary family homeless shelter. **Exclusion criteria:** None stated.	Temporary family homeless shelter.	Tertiary / treatment / secondary	Goal-orientated **therapy intervention** (n = 24) aiming to reduce family violence, decrease maladaptive coping strategies, and increase psychological well-being of mothers who have experienced IPV. Involved three parts; a mother’s group, children’s group, and joint mother-child group delivered by female therapists (two masters-level counsellors and two in training). Mother’s group (4–5 mothers) was underpinned by cognitive behavioural, motivational interviewing and transtheoretical models and involved education in adaptive/non-adaptive coping strategies, mothers self-identifying adaptive/non-adaptive coping strategy they would like to increase/decrease (most often relational, personal, or functional goal), working through ways to achieve this. Mothers were encouraged to find support for common challenges from the group whilst also being supported to work on their individualised goals. Children’s group (4–5 children) followed a similar format, empowering children to identify aspects of their lives they had the ability to change, identifying a specific goal to achieve, and committing to achieving this goal. Joint mother-child group (8–10 participants) discussed themes from individual groups within the family context. In addition, mothers received age-appropriate childcare and referrals to community-based services.	Delivered face-to-face in an undisclosed community setting over a period of 5 weeks (each group five sessions, weekly); hosted within community IPV.	Active control (n = 22) mothers and children received emotion-focused therapy (see description below).	**Authors describe as uni-directional**; DVA may lead to MH or SU or child abuse. MH/SU/child abuse conceptualised as maladaptive coping strategies for women experiencing trauma. Short term benefit for dealing with stress but detrimental in long term. **Intervention treats as uni-directional–DVA focused**; target relationship between DVA and MH/SU (coping strategies related to DVA).	**Post-intervention:** **MH** = mothers’ depression measured using CES-D. **SU** = mothers’ alcohol use measured using own questions. **No follow-up.**
**15b. McWhirter (2011)**^**3**^ (reference number: [89])	Southwestern metropolitan area, U.S. 47% White	Mothers (and their children) who have experienced IPV within last year, reported their child being present for at least one incident of IPV in last year, and were currently residing in a temporary family homeless shelter. **Exclusion criteria:** None stated.	Temporary family homeless shelter.	Tertiary / treatment / secondary	Emotion-focused **therapy intervention** (n = 22) aiming to reduce family violence, decrease maladaptive coping strategies, and increase psychological well-being of mothers who have experienced IPV. Involved three parts; a mother’s group, children’s group, and joint mother-child group delivered by female therapists (two masters-level counsellors and two in training) and was underpinned by behavioural and gestalt approaches. Mothers’ group (4–5 mothers) focused on the ‘here and now’ learning, developing healthy group relationships in order to increase social support, and an educational curriculum which explored mothers’ belief systems, understanding forms of abuse, understanding and expressing feelings, recognising health relationships, and finding health ways to cope with stress. Similarly, children’s group (4–5 children) focused on helping children develop strategies and techniques for identifying negative feelings associated with family transitions, understanding, expressing, and integrating feelings, learning about abuse, and ways to stay safe in an abusive household. The focus was on developing personal awareness, dealing with emotions, family pressure, and peer pressure, developing good social support networks, and handling conflict. Joint mother-child group (8–10 participants) discussed themes from individual groups within the family context. In addition, mothers received age-appropriate childcare and referrals to community-based services.	Delivered face-to-face in an undisclosed community setting over a period of 5 weeks (each group five sessions, weekly); hosted within community IPV.	Active control (n = 24) mothers and children received goal-orientated therapy (see description above).	**Authors describe as uni-directional**; DVA may lead to MH or SU or child abuse. MH/SU/child abuse conceptualised as maladaptive coping strategies for women experiencing trauma. Short term benefit for dealing with stress but detrimental in long term. **Intervention treats as uni-directional–DVA focused**; target relationship between DVA and MH/SU (coping strategies related to DVA).	**Post-intervention:** **MH** = mothers’ depression measured using CES-D. **SU** = mothers’ alcohol use measured using own questions. **No follow-up.**
**20. Rotheram-Borus et al. (2003)** Linked: Rotheram-Borus et al. (2001)xya (reference number: [95, 109])	New York City, U.S. Parents 50% Latino; Children 49.5% Latino	Parents (aged 25–70 years) living with HIV who had at least one adolescent child (biological or adopted) aged between 11–18 who lived with them and whose case manager felt the study was in their best interests. **Exclusion criteria:** None stated.	Log at New York City Division of AIDS services from August 1993 to March 1995.	Tertiary / secondary	**Coping skills intervention** (n = 153 parents; n = 206 children) aiming to address potential long-term negative impacts of HIV on families including SU and MH for parents living with HIV and their children. Parents and children received elements of the intervention delivered by social workers and graduates in clinical psychology. Part 1 = parent only group discussed coping skills around HIV status, dealing with related MH and SU, and decisions around disclosing status to children. Part 2 = parent and child group which involved some sessions for parents and children separately and some for parents and children together. Focused on maintaining family routines, parental support to avoid high-risk behaviours, custody plans, coping skills for adolescents, skills to reduce high-risk behaviours and emotional distress.	Delivered in a community centre over a period of 2 years (24 sessions, frequency not stated); hosted within social work.	Usual care control (n = 154 parents; n = 207 children).	**Authors do not state**; both MH and SU related to HIV status. **Intervention treats as co-occurring**; MH and SU both coping strategies related to HIV. Targeted as co-occurring issues rather than clustering.	**No post-intervention.**^**4**^ **Follow-up (24 months post-intervention):** **MH** = parents’ general MH measured using BSI. **SU** = parents’ general substance misuse (current use and relapse) measured using self-report.
**21. Rotheram-Borus et al. (2012)**	Los Angeles, California, U.S. 67.6% Latino	Mothers living with HIV (aged 21–69 years) who are the primary female caregiver for at least one child aged between 6–20 years and were enrolled in HIV-related care. **Exclusion criteria:** None mentioned.	Medical and non-medical health care facilities (HIV/AIDS service organisations) and peer referral.	Secondary / tertiary	**Coping skills intervention** (n = 172 mothers, n = 139 children) aiming to address potential negative impacts of living with HIV (including MH and SU) on mothers living with HIV and their adolescent children. Mothers and children received elements of the intervention delivered by intervention facilitators. Part 1 = mothers group sessions (5–8 mothers) focused on promoting positive parenting, improving MH, reducing risky behaviour (including SU) and increase use of medicines (do not describe how). Part 2 = mother and child group which involved some sessions for mothers and children separately and some for mothers and children together. Children’s sessions focused on family relationships, MH, SU and other risky behaviours and school retention (do not describe how). Authors provide a link to intervention manual but this does not work.	Delivered in community settings (e.g., HIV/AIDS clinics, community space in children’s’ hospital, community centres, uni campus) over a period of 8 weeks (16 sessions overall, 12 for mother alone, 12 for children alone, 4 for mother and children together, sessions twice weekly); not clear who it was hosted by.	Usual care control (n = 167 mothers, n = 120 children) received intervention after the last follow-up timepoint (18 months). No other information given about the care they received.	**Authors do not state**; MH and SU both risk factors related to HIV status. **Intervention treats as co-occurring**; MH and SU both coping strategies related to HIV. Targeted as co-occurring issues rather than clustering.	**Post-intervention (6 months post baseline):** **MH** = mothers depression, anxiety and general MH measured using BSI. (reference number: [64]) **SU** = mothers’ alcohol, marijuana, and hard drug use measured using self-report. **Follow-up (12 months post-intervention):** Same as above.
**24. Wu and Slesnick (2019)** Linked: Slesnick and Zhang (2016); Wu and Slesnick (2020); Zhang (2018) (reference number: [90, 110–112])	Midwestern city, U.S. (state not stated) 53.6% Non-Hispanic White	Mothers meeting diagnostic criteria for alcohol or drug use disorder (DSM-IV), seeking treatment for this disorder, and who had a biological child aged 8–16 years who lived with them (≥50% of time in past 2 years or 100% in past 6 months). Child with most severe SU or behavioural problems participated. **Exclusion criteria:** None stated.	SU treatment centre.	Treatment	**Therapy** intervention (n = 123) called ‘ecologically based family therapy’ aiming to reduce mothers’ SU by targeting dysfunctional family interactions between mothers and children. The intervention is delivered to the whole family where possible and involves 12 sessions of family systems therapy delivered by EBFT therapists (licensed counsellors/clinical graduates). The first sessions involved engaging with and assessing the needs of families and encouraging them to consider SU and solutions to SU stemming from family relations. The remaining sessions focused on addressing dysfunctional relationships that may contribute to SU, harnessing interactions that were protective, developing and practicing problem solving skills, discussing how children can support mothers’ abstinence from SU, and CBT to change individual thoughts, coping skills etc.	Delivered in the home or office setting over a maximum period of 6 months (12 sessions, frequency not stated); hosted within community SU services.	Active control (n = 60) received 12 sessions of ‘Women’s Health Education’; a manualised intervention delivered by a therapist who provides education on pregnancy and childbirth, sexual behaviour, and women’s bodies.	**Authors describe as bi-directional**; depression (MH) and SU co-occur. Depression (MH) likely to lead to more SU (used as a coping mechanism to deal with unpleasant emotions and improve mood). SU behaviour reinforced and continued to maintain positive effects, leading to potential dependence. Likewise, SU likely to lead to depression (MH) due to changes in brain structures reducing positive affect and increasing dysphoric mood. **Intervention treats as uni-directional–SU focused;** targets SU and expects improvements in MH.	**Post-intervention:** **MH** = mothers’ depression measured using BDI-II. **SU** = mothers’ alcohol, marijuana, and hard drug use measured using Form-90. **Follow-up (6 months post-intervention):** Same as above.
**27. Suchman et al.** (**2010**; 2011) (reference number: [85, 113])	Midsized urban city, U.S. (state not stated) Ethnicity not reported	Mothers enrolled in outpatient SU treatment who were caring for a child aged 0–36 months old. **Exclusion criteria:** actively suicidal, homicidal, severely cognitively impaired, disengaged from SU treatment, not fluent in English.	Outpatient SU treatment clinic (referrals from and research staff visits to).	Tertiary	**Therapy intervention** (n = 23) called ‘The Mothers and Toddlers Program’ (MTP) aiming to improve mothers mentalisation and maternal representations of caregiving to improve parenting / reduce potential risk of child maltreatment. Mothers received individual therapy sessions delivered by trained therapists (who had experience working with similar groups). The first session focused on developing a supportive relationship between the mother and therapist and outlining what the intervention would involve (mothers were given the option to contact staff out of hours to deal with crises). The first sessions involved helping the mother work through any issues they were having meeting basic needs and then therapeutic work began. Session discussions were guided by the mother (with the therapist inviting them to consider the child when appropriate) and involved discussing stressful situations the mother had experienced (that may have made reflective functioning difficult). Therapists encouraged the mother to review these situations in depth, actively mentalising what happened, their thoughts, feelings and behaviours, and the child’s thoughts, feelings, and behaviours. Developmental guidance and/or parenting strategies to promote attachment were suggested where appropriate. Therapists used videoed interactions between mother-child to discuss child’s needs and feelings and would verbalise child’s potential thoughts when mother and child were together. Other staff provided out of hours support where requested.	Delivered in clinic over a period of 12 weeks (12 sessions, weekly); hosted within community SU.	Active control (n = 24) received individual case management from parent education counsellors weekly, leaflets based on their interests/needs on child development, and standard care as provided by SU clinic.	**Authors describe as uni-directional**; SU leads to high levels of stress and changes in neurobiological reward systems that help adapt to stress. This can lead to negative emotions which may mean mothers don’t experience sense of reward they would normally experience during parenting. **Intervention treats as co-occurring**; target parenting and relationship between mother and child rather than MH or SU concurrently.	**Post-intervention:** **MH** = mothers’ depression general MH measured by BDI and BSI, respectively. **SU** = mothers’ general drug use (opiate, cocaine, cannabis, metabolites) measured using urine toxicology. **Follow-up (6 weeks post-intervention):** Same as above.
**28. Suchman et al. (2017)** (reference number: [91])	Small, urban, Northeastern city, U.S. 77% Caucasian	Mothers enrolled in outpatient SU treatment who have at least one child aged 11–60 months old. **Exclusion criteria:** doesn’t speak English, severe MH, sig cognitive impairment, inpatient hospitalisation or detoxification, child has serious illness, child has sig developmental delay.	Outpatient SU treatment clinic.	Tertiary	**Therapy intervention** (n = 40) called ‘Mothering From the Inside Out’ (MIO) aiming to improve mothers mentalisation and maternal representations of caregiving to improve parenting / reduce potential risk of child maltreatment. Mothers received individual therapy sessions delivered by clinical psychologists. Session discussions were guided by the mother (with the therapist inviting them to consider the child when appropriate) and involved discussing stressful situations the mother had experienced (that may have made reflective functioning difficult). Therapists encouraged the mother to review these situations in depth, actively mentalising what happened, their thoughts, feelings and behaviours, and the child’s thoughts, feelings, and behaviours. Developmental guidance and/or parenting strategies to promote attachment were suggested where appropriate. By providing mothers with a supportive environment to engage in reflecting on these situations, the interventions long term goals were to help mothers develop the capacity for emotional regulation, human attachment (rather than SU), and ability to understand and engage with their child in a way that supports their development. Also had access to childcare and bus passes to clinic.	Delivered in clinic over a period of 12 weeks (12 sessions, weekly); hosted within community SU.	Active control (n = 47) mothers received a manualised parenting education intervention delivered by specialists in parenting education to whom they had been assigned. Involved meeting weekly to discuss parenting strategies/child development around a leaflet the mother had selected and specifically addressed issues pertinent to mothers experiencing SU. Also had access to childcare and bus passes to clinic.	**Authors describe as uni-directional**; SU can lead to heightened stress activation (due to impact on dopaminergic neural pathways) which can lead to vulnerability to relapse and diminished reward/heightened stress as mother enters parenthood. Coupled with limited coping skills this means mothers are vulnerable to MH (which is common in early stages of SU recovery). **Intervention treats as co-occurring**; target parenting and relationship between mother and child rather than MH or SU concurrently.	**Post-intervention:** **MH** = mothers’ depression general MH measured by BDI and BSI, respectively. **SU** = mothers’ heroin, opioid, and cocaine use measured using TLFB. **Follow-up (3 months post-intervention):** Same as above.
**33. Volpicelli et al. (2000)** (reference number: [66])	U.S. (state not stated) 96.4% African American	Mothers diagnosed with cocaine-dependency who have custody of at least one child under the age of 4 or were currently pregnant. **Exclusion criteria:** psychotic, homicidal, suicidal, unstable medical condition, opioid dependent.	Referrals from community agencies, hospitals, and public transportation advertisements.	Treatment / secondary	**Multicomponent intervention** (n = 42) called ‘psychosocially enhanced treatment program’ (PET) aiming to increase retention in SU treatment, reduce SU, and improve psychosocial functioning. Mothers were given access to a variety of services available in outpatient SU treatment in addition to standard care (group-based therapy sessions covering topics such as self-esteem, co-dependence, understanding addiction etc. twice per week, onsite childcare, access to women’s-only group). Included parenting classes, GED classes, unlimited individual crisis management counselling delivered by therapists who were drug counsellors, and access to staff psychiatrist. Referrals were also made to community services where necessary.	Delivered in SU clinic, authors do not state the duration but report that mean number of weeks mothers attended was 15.4 (SD = 12.8) (no set number of sessions or frequency; mothers allowed to access services as often or as little as they like); hosted within community SU.	Active control (n = 42) mothers received case management orientated program (CM) in addition to standard care (see description opposite). Involved access to 15-minute appointments per week with social worker who provided case management which involved referrals to community-based services.	**Authors describe as uni-directional**; women using drugs (SU) more likely to have problems with MH than men using drugs. **Intervention treats issues as bi-directional;** offering concurrent support for SU and MH.	**Post-intervention (treated as 9 months post baseline):** **MH** = mothers’ general MH measured using BSI. **SU** = mothers’ cocaine use measured using ASI (past 30 day use) and urinalysis (total number of free urines collected over course of study). **Follow-up (3 months post-intervention):** Same as above.
**34. Walkup et al. (2009)** (reference number: [67])	Najavo and White Mountain Apache reservations in New Mexico and Arizona, U.S. 100% American Indian	American Indian mothers (aged 12–22 years; ≤28 weeks gestation).^5^ **Exclusion criteria:** extreme medical, legal, or social problems (including incarceration, SU that requires residential care/extended hospitalisation).	Pre-natal and school-based clinics.	Tertiary / secondary	**Home visiting** intervention (n = 81) called ‘Family Spirit’ aiming to prevent negative behavioural and health outcomes in American Indian mothers and their children. Mothers received home visits from native paraprofessionals. The intervention followed the procedures set out by ‘Healthy Families America/Indiana’ and the curriculum included a focus on age-appropriate parenting, family planning, SU prevention, coping skills and problem solving all of which were culturally informed. Paraprofessionals were sensitive to the cultural preferences of mothers throughout. The link to additional information about the intervention does not work.	Delivered in home setting or confidential community setting of mothers’ choice over a period of 7.5 months (up until child 6 months of age; 25 sessions, frequency not stated); hosted within home visiting services.	Active control (n = 86) mothers also received home visits from native paraprofessionals. However, the content of the sessions differed from that of the intervention. The curriculum focused on education related to breast feeding and nutrition. The control group home visiting program involved 23 sessions (frequency and duration not stated).	**Authors do not state**; MH and SU risk factors related to being a young, American Indian mother. **Intervention treats issues as co-occurring**; tackling MH and SU separately.	**Post-intervention:** **MH** = mothers’ depression measured using CES-D. **SU** = mothers’ alcohol and illegal substances use measured using items developed by SAMHSA. **Follow-up (6 months post-intervention):** Same as above.

**NB**. Green coloured cells indicate studies that have had, or report to have, combined positive impacts on two or more outcomes. ^1^ SU measured at child 9 months of age rather than post-intervention, latest timepoint available. ^2^ Only follow-up intervention group rather than intervention and control. ^3^ McWhirter (2011) conducted an RCT with two intervention groups that acted as controls for one another hence there are two separate entries for this study, one with goal-orientated therapy as intervention and one with emotion-focused therapy as intervention. ^4^ Rotheram-Bours et al. (2011) only measure MH at post-intervention (not SU) and therefore, combined impacts cannot be examined at this timepoint. ^5^ Walkrup et al. (2009) split analysis by mothers aged <18 years and 18+ years. Focus on analysis of 18+ years. ADD = Alcohol Dependency Data; ASI = Addiction Severity Index; BDI = Beck Depression Inventory; CES-D = Center for Epidemiologic Studies Depression Scale; DVA = Domestic violence; EPDS = Edinburgh Postnatal Depression Scale; IPV = Intimate Partner Violence; MH = Mental ill-health; SF-36 = Short Form-36; SU = Substance misuse; TLFB = Timeline Followback Interview; OCD = obsessive compulsive disorder; SCL-90-R = Symptom Checklist 90 Revised.

**Table 4:** Study characteristics for studies measuring DVA, MH, and SU.

Study characteristics for studies measuring DVA, MH, and SU									
Study	Context	Population	Recruitment setting(s)	Level of prevention	Intervention form and function	Intervention duration and setting	Control(s)	Relationship between DVA/MH/SU	DVA/MH/SU outcomes measured*
**2. Duggan et al. (2007)** (reference number: [101])	Alaska, U.S. 55% Caucasian	Families (pregnant or at birth) deemed at risk of child maltreatment due to parental DVA, MH, or SU, or other related factors, scoring ≥25 on Kempe Family Stress Checklist, and able to speak sufficient English to take part in study. **Exclusion criteria:** Previously enrolled on home visiting intervention.	Hospitals either prenatally or at birth.	Tertiary	**Home visiting** intervention (n = 162) called ‘Healthy Start Alaska’ (HSA) aiming to help prevent child maltreatment by promoting positive parenting and child development, and reduce malleable risk factors (i.e., DVA, MH, and SU). Mothers (and fathers where possible) received home visits from trained paraprofessional home visitors. Sessions involved providing parents with information, demonstrating positive parenting practices through role play and reinforcement, helping parents set family-initiated goals within an Individual Family Support plan (IFSP), supporting parents through crises, and recognising and responding to parental DVA, MH, and SU through encouraging parents to seek help from community-based services.	Delivered in home setting over a period of 3+ years (number of sessions varies, frequency varies tends to be weekly in first 6–9 months then less frequently as family functioning improves); hosted within established home visiting sites.	Minimal care control (n = 163) received referrals to other community services (do not state who delivered these referrals).	**Authors describe as uni-directional**; coercive relationships (DVA) can make stress unmanageable, and this can lead to depression (MH) and SU (as well as child maltreatment). **Intervention treats as co-occurring**; separate referrals for DVA/MH/SU.	**Post-intervention (treated as child 2 years of age):** **DVA** = mothers’ physical, psychological, and injury related victimisation / perpetration measured using CTS2. **MH** = mothers’ depression and general MH measured using CES-D and MHI-5, respectively. **SU** = mothers’ alcohol and drug use measured using self-report and CAGE (for alcohol use). **No follow-up.**
**3. Duggan et al.** (1999; **2004**); McFarlane et al., (2013) Linked: Bair-Merritt (2010); Bair-Merritt (2010) (reference number: [105, 114–117])	Oahu, Hawaii, U.S. 33.6% Native Hawaiian or Pacific Islander	Families (pregnant or at birth) deemed at risk of child maltreatment due to parental DVA, MH, or SU, or other related factors, scoring ≥25 on Kempe Family Stress Checklist, and not currently under child protection services. **Exclusion criteria:** None stated.	Hospitals (maternity obstetrical units) either prenatally or at birth.	Tertiary	**Home visiting** intervention (n = 373) called ‘Hawaii Healthy Start Programme’ (HSP) aiming to help prevent child maltreatment and promote positive child development by improving family functioning. Mothers (and fathers where possible) received home visits from trained paraprofessional home visitors. Sessions involved providing parents with information, demonstrating positive parenting practices through role play and reinforcement, co-developing family-related goals within an Individual Family Support plan (IFSP) which is used as a guide throughout the intervention, supporting parents through crises, and recognising and responding to parental DVA, MH, and SU through providing emotional support and encouraging parents to seek help from community-based services.	Delivered in home setting over a period of 3–5 years (number of sessions varies, frequency varies); hosted within established home visiting sites.	Control (n = 270) services/care received is not described. Test control group (n = 45) included for purposes of funders due to concerns about impact of multiple testing. Only followed up for a few timepoints and are not reported on.	**Authors describe as uni-directional**; coercive relationships (DVA) can make stress unmanageable, and this can lead to depression (MH) and SU (as well as child maltreatment). **Intervention treats as co-occurring**; separate referrals for DVA/MH/SU.	**Post-intervention (child 3 years of age):** **DVA =** mothers’ physical, psychological, and injury related victimisation / perpetration measured using CTS2. **MH =** mothers’ depression and general MH measured using CES-D and MHI-5, respectively. **SU =** mothers’ alcohol and drug use measured using self-report and CAGE (for alcohol use). **Follow-up (4–6 years post-intervention):** Same as above other than mothers’ drug use which was measured using ASI.
**5. Fergusson et al.** (**2006**; 2013) Hand linked: Fergusson et al. (2005; 2012) (reference number: [106, 118, 119])	Christchurch, New Zealand ~25.8% Mäori (based on those who completed follow-up assessments)	New parents (within 3 months of childbirth) screened at risk of child maltreatment using Hawaii Healthy Start Program Screening Tool or deemed at risk by nurses responsible for recruitment. **Exclusion criteria:** None stated.	Home during visits conducted by Plunkett Nurses who visit families within three months of the birth of a new child (free service).	Tertiary	**Home visiting** intervention (n = 220) called ‘Early Start Home Visiting’ aiming to improve child health, reduce child abuse, promote positive parenting, maternal health and well-being, and family economic and material well-being. Parents received home visits from family support workers. Initial sessions involved assessing the family’s level of need and strengths, followed by sessions which involve encouraging family led problem-solving to overcome issues and providing general support and mentoring throughout child’s preschool years.	Delivered in the home setting over a period of 3 years (number of sessions and frequency varies depending on family level of need); hosted within established home visiting services.	Usual care control (n = 223) (do not describe who this was provided by or what this involved).	**Authors do not state.** **Intervention treats as co-occurring**; separate aspects of intervention targeting each.	**Post-intervention:** **DVA** = mothers’ physical victimisation measured using CTS2. **MH** = mothers’ depression measured using items from CIDI. **SU** = mothers’ alcohol and drug use measured using items from CIDI. **Follow-up (2–6 years post-intervention):** **DVA** = mothers’ physical and psychological victimisation and perpetration measured using CTS2. **MH and SU** = Same as above.
**8. Slesnick and Erdem (2013)**; Guo et al. (2016) (reference number: [82, 120])	Columbus, Ohio, U.S. 75% African / African American	Homeless mothers living in a public or private temporary living shelter/public or private place not designed for regular sleeping who had a biological child aged 2–6 years in their care and met criteria for substance abuse or dependence (DSM-IV)	Family homeless shelter. **Exclusion criteria:** None stated.	Treatment	**Multi-component** ecologically based intervention (n = 30) aiming to reduce mothers’ SU (and related disorders) and promote independent living. Involved three main components: housing support, case management and counselling. In terms of housing support, mothers were provided with their choice of accommodation and given rental/utility assistance for 3 months. In terms of case management, mothers received ongoing assistance with any ongoing needs (including referrals to food pantries, obtaining food stamps etc.). In terms of counselling, mothers received operant-based Community Reinforcement Approach counselling designed to address SU but also other related issues including DVA and MH. This was delivered by white, female masters-level therapists (graduates from The Ohio State University Couple and Family Therapy program or Clinical Social Work program).	Delivered in an undisclosed setting over a period of 6 months. This included 6 months of case management (26 sessions, frequency not stated) and counselling (20 sessions, frequency not stated), and 3 months of concurrent housing support; hosted within housing/community SU.	Active control (n = 30) received emergency shelter accommodation for up to three weeks followed by ‘rapid re-housing’ which involves partner agencies providing independent housing/shelter providing 3 months of subsided housing and encouraging mothers’ to secure employment during this time to take charge of payments. Also received normal services through community.	**Authors describe as uni-directional**; homelessness (and having a dependent child) is linked with MH and SU. More frequent SU predicts higher rates of IPV (DVA). **Intervention treats as bi-directional**; providing Community Reinforcement Approach counselling to tackle all concurrently.	**Post-intervention:** **DVA** = mothers’ emotional abuse victimisation measured using WEB **MH** = mothers’ depression and general MH measured using BDI-II and SF-36 MH subscale, respectively. **SU** = mothers’ alcohol and drug use measured using The Form90 Interview and urine toxicology. **Follow-up (3 months post-intervention):** Same as above.
**9. Jack et al. (2019)** (reference number: [79])	Multiple states, U.S. 50.7% Hispanic / Latina	First-time mothers (aged 16+) who had met criteria for entering the NFP program (<28 weeks gestation, living in poverty, first-time birth) and had not yet completed 4^th^ NFP nurse visit. **Exclusion criteria:** None stated.	Home visiting sites across the U.S.	Secondary / treatment	**Home visiting supplement** intervention (n = 229) aiming to improve mothers’ quality of life and reduce risk of IPV (and other related issues). Mothers received NFP home visits (as described in control group section) in addition to a multi-component IPV intervention delivered by nurses. Involved extensive nurse training in IPV, and clear guidance around reflective supervision and procedures to implement a multi-component, tailored intervention which involved nurses conducting universal safety assessments and identifying IPV, providing an empathetic response to disclosures, conducting risk assessments and empowerment intervention (including safety planning and discussing cycle of abuse), conducting MH, SU and readiness to address safety assessments. This information was then used to tailor a response providing motivational interviewing, safety planning, education of health effects, goal setting and providing referrals to and facilitating access to external services (e.g., DVA, MH, SU, housing, legal, financial support).	Delivered in the home setting over a period of 2 years+ (pregnancy–child’s 2^nd^ birthday); hosted within established home visiting sites.	Active control (n = 263) received NFP home visits from a nurse over a period of 2 years+ (pregnancy–child’s 2^nd^ birthday, max 64 sessions, frequency not stated). Sessions involved discussing maternal health, environmental health, role as a mother, relationships, life course development and services. IPV was screened for at three separate points in time. If mothers disclosed IPV their safety was assessed, they were provided with information, and referred to community services.	Not stated. **Intervention treats as co-occurring;** although addressing them all (and recognising DVA may lead to MH and SU) involves empowerment intervention for DVA, and identification and referral for MH and SU tackling them with separate, distinct approaches.	**Post-intervention:** **DVA** = mothers’ physical, emotional, and severe abuse and harassment measured using CAS. **MH** = mothers’ PTSD, depression, and general MH measured using SPAN, PHQ-9, and SF-12 MH subscale, respectively. **SU** = mothers’ alcohol and drug use measured using TWEAK and DAST, respectively. **No follow-up.**
**17a. Olds et al. (2004)**^**1**^ (reference number: [103])	Denver, Colorado, U.S. ~46.6% Mexican American (based on those who completed follow-up assessments)	Low income (at 133% of federal poverty level or had no private insurance) women experiencing their first live birth. **Exclusion criteria:** None stated.	Antepartum clinics.	Tertiary / secondary	**Home visiting** intervention (n = 245) called NFP aiming to improve maternal and foetal health, improve child development, enhance mothers’ development. Mothers received home visits from paraprofessionals with strong people skills and high school education. Visits involved promoting mothers’ health-related behaviours, parenting skills, and planning for the future (family planning, education, and employment), helping mothers improve social relationships, and promoting their use of external services to address needs.	Delivered in home setting over a period of 2 years+ (pregnancy–child’s 2^nd^ birthday, number of sessions and frequency varies); hosted within established home visiting sites.	Minimal care control (n = 255) mothers received free child development screenings and referrals at 6, 12, 15, and 21 months.	**Authors do not state.** **Intervention treats as co-occurring**; separate referrals for DVA/MH/SU.	**DVA/MH/SU not measured at post-intervention.** **Follow-up (24 months post-intervention):** **DVA =** mothers’ physical violence victimisation measured using CTS. **MH =** mothers’ general MH not reported how this was measured. **SU =** mothers’ alcohol and marijuana use measured using own measure.
**17b. Olds et al. (2004)**^**1**^ (reference number: [103])	Denver, Colorado, U.S. ~46.6% Mexican American (based on those who completed follow-up assessments)	Low income (at 133% of federal poverty level or had no private insurance) women experiencing their first live birth. **Exclusion criteria:** None stated.	Antepartum clinics.	Tertiary / secondary	**Home visiting** intervention (n = 235) called NFP aiming to improve maternal and foetal health, improve child development, enhance mothers’ development. Mothers received home visits from nurses. Visits involved promoting mothers’ health-related behaviours, parenting skills, and planning for the future (family planning, education, and employment), helping mothers improve social relationships, and promoting their use of external services to address needs.	Delivered in home setting over a period of 2 years+ (pregnancy–child’s 2^nd^ birthday, number of sessions and frequency varies); hosted within established home visiting sites.	Minimal care control (n = 255) mothers received free child development screenings and referrals at 6, 12, 15, and 21 months.	**Authors do not state.** **Intervention treats as co-occurring**; separate referrals for DVA/MH/SU.	**DVA/MH/SU not measured at post-intervention.** **Follow-up (24 months post-intervention):** **DVA =** mothers’ physical violence victimisation measured using CTS. **MH =** mothers’ general MH not reported how this was measured. **SU =** mothers’ alcohol and marijuana use measured using own measure.
**18. Olds et al.** (**2007**; 2010; 2019) Hand linked: Olds et al. (2004) (reference number: [102, 121–123])	Memphis, Tennessee, U.S. 92% Black	Black, low income, pregnant women (<29 weeks gestation) who had two or more of the following risk factors; unmarried, <12 years education, and/or unemployed, and who had no specific chronic illnesses that might impact foetal growth and development. **Exclusion criteria:** None stated.	Obstetric clinics.	Tertiary / secondary	**Home visiting** intervention (n = 228) called NFP aiming to improve pregnancy outcomes, improve child development, enhance mothers’ development. Mothers received home visits from nurses. Visits involved promoting mothers’ health-related behaviours, parenting skills, and planning for the future (family planning, education, and employment), helping mothers improve social relationships, and promoting their use of external services to address needs. Nurses tried to involve other family members/friends in visits where possible.	Delivered in home setting / over telephone over a period of 2 years and 3 months (pregnancy–child’s 2^nd^ birthday, number of sessions and frequency varies); hosted within established home visiting sites.	Minimal care control (n = 515) mothers received free developmental screening and referral for children at 6, 12, 15, and 21 months and free transportation to prenatal care.	**Authors do not state**; all linked to increased chance of poor child outcomes / maltreatment. **Intervention treats as co-occurring**; separate referrals for DVA/MH/SU.	**DVA/MH/SU not measured at post -intervention.** **Follow-up (16 years post-intervention):** **DVA =** mothers’ physical violence victimisation measured using CTS. **MH =** mothers’ depression and anxiety measured using BSI and BAI, respectively. **SU =** mothers’ substance use measured using drug use screening inventory.
**19. Ondersma et al. (2017)** (reference number: [63])	Indiana, U.S. 37.5% Black African American	Pregnant women (≥18 years, no more than 45 days before expected due date) who were at risk of child maltreatment as defined by high score on Kempe Family Stress Checklist (evaluating presence of DVA, MH, and SU, and related factors). **Exclusion criteria:** None stated.	Home visiting sites.	Tertiary / secondary	E-intervention **home visiting supplement** (n = 142) aiming to reduce child maltreatment risk factors including DVA, MH, and SU in order to reduce harsh parenting and increase intervention adherence. Mothers received an e-intervention in addition to normal home visiting services (see control group description). This was delivered online on a PC tablet (provided by home visitors) and included a narrator, audio (via headphones), interactive elements, and videos. The sessions (20 mins each) focused on providing motivational interviewing to engage mothers in the home visiting intervention and target DVA, MH, and SU, cognitive retraining to model ways to parent when confronted with difficult child behaviours, and SafeCare to discuss home safety, medical decisions, and accident prevention.	Delivered over the internet over a period of 6 months (or until all 8 sessions complete, frequency of sessions varies); hosted within established home visiting sites.	Two control groups^2^: 1) Active control (n = 141) receiving HFA home visiting program alone. Involved weekly home visits in the first 6 months, vary thereafter depending on family’s level of need (every other week and then monthly/ quarterly). Home visits aimed to promote family functioning, parent-child relationships and child health and development. 2) Minimal care control (n = 130) receiving community referrals.	**Authors do not state**; all considered risk factors for child maltreatment. **Intervention treats as co-occurring**; motivate parents to get help with DVA/MH/SU concurrently using motivational interviewing but support is provided through separate services for DVA, MH, and SU.	**Post-intervention:** **DVA =** mothers’ physical assault and injury victimisation and perpetration measured using CTS2. **MH =** mothers’ depression measured using EPDS. **SU =** mothers’ alcohol and drug use measured using ASSIST. **Follow-up (6 months post-intervention):** Same as above.
**22. Rotheram-Borus et al. (2015)** Linked: Rotheram-Borus et al. (2014; 2019) Hand linked: Rotheram-Fuller et al. (2018); Le Roux et al. (2013) (reference number: [80, 124–127])	Cape Town, South Africa 100% Black African	Low-income pregnant women (aged 18+) residing in urban areas near Cape Town. **Exclusion criteria:** None stated.	Community; low-income urban areas.	Secondary	**Home visiting** intervention (n = 644) called the ‘Philani+ Programme’ (PHILANI+) aiming to reduce alcohol use and improve HIV related behaviours. Mothers received home visits from paraprofessionals (community health workers; CHW) who had experience raising their own healthy children and had good problem solving and social skills. The sessions involved education around general maternal and child health, HIV/TB, alcohol use, mental health, and nutrition, and dealing with crises. CHW delivered a brief alcohol intervention as part of this which involved discussing the consequences of alcohol use on children and the amount of alcohol currently being used by the mother (compared to recommended quantities). The intervention was guided by CBT principles and involved role play, goal setting, problem solving, relaxation, assertiveness, and shaping. CHW received regular supervision and logged their contacts with mothers on a mobile app.	Delivered in the home setting over a period of 18 months+ (number of sessions varies, frequency varies); hosted within established home visiting sites.	Usual care control (n = 594) involved accessing services situated ≤5km away which provided HIV related testing and medical care as well as postnatal visits at 1 week (although authors describe this as inconsistent). HIV care was also available during four antenatal visits and within HIV care clinics postpartum, during which time well-baby visits were also provided.	**Authors describe as uni-directional**; women with depression (MH) are at increased likelihood of alcohol use (SU) which is often implicated in IPV (DVA) and all can have a negative impact on children. HIV impacts relationships with partners, children, MH, and physical health. **Intervention treats as uni-directional–SU focused**; targeting alcohol use specifically and hoping for decreased MH and DVA.	**Post-intervention:** **DVA =** mothers’ physical violence victimisation measured using questions adapted from Jewkes et al. **MH =** mothers’ depression measured using EPDS and GHQ-12. **SU =** mothers’ alcohol use measured using AUDIT-C latent variable. **Follow-up (18 months post-intervention):** Same as above.
**23. Silovsky et al. (2011)** (reference number: [68])	Rural country in South West, U.S. 71.3% White	Caregivers (aged 16+) who had at least one child ≤5 years and displayed at least one of the following risk factors for child maltreatment; IPV, MH, or SU. **Exclusion criteria:** current child welfare or service involvement, two prior child welfare referrals, caregiver perpetrated child sexual abuse, severe psychosis, severe MH or any other issue that might prevent caregiver from providing valid self-report data.	Various; child maltreatment prevention and parenting services, referrals from professionals and community-based organisations (e.g., schools, faith organisations).	Tertiary	**Home visiting supplement** (n = 48) called ‘SafeCare+’ aiming to prevent child maltreatment by reducing parent IPV, depression and SU. Parents^3^ received home visits from home visitors who were supported my IPV MH and SU professionals. The standard SafeCare home visiting programme is underpinned by an eco-behavioural approach targeting different levels of the ecological model of child maltreatment and parenting behaviours related to ‘child health, home safety and cleanliness, and parent-child bonding’ through modelling, practice and feedback, ongoing measurement of behaviours, and parent training. It also involves recognising and responding to factors such as IPV, depression and SU and recognising the role of poverty. The home visiting supplement (leading to SafeCare+) expands on this to provide additional training in identifying and responding to IPV, depression and SU as well as in motivational interviewing with the view that this will encourage parents to address these issues.	Delivered within the home setting over a period of less than 6 months (number of sessions and frequency not stated); hosted within established home visiting services.	Active control (n = 57) received a community MH program which offered individual and family therapy and case management. Support was tailored to families’ needs based on what they wanted to address (e.g., SU, depression, anxiety, anger management). **Intervention treats as co-occuring;** identifying and responding to IPV, depression and SU as co-occurring risk factors for child maltreatment.	**Authors do not state**; all three known risk factors for child maltreatment.	**Post-intervention (treated here as 6 months):** **DVA =** parents’^3^ physical, psychological, sexual and injury-related victimisation measured using CTS2. **MH =** parents’^3^ depression measured using BDI-II. **SU =** parents’^3^ alcohol and drug use measured using DIS module. **Follow-up (6 months post-intervention):** Same as above.
**26. Stover et al. (2019)** (reference number: [84])	Large metropolitan area in South East, U.S. 74% Euro-American heritage	Fathers (English speaking) in residential SU treatment who were in contact with children and reported physical or psychological IPV towards female co-parent in last 12 months. **Exclusion criteria:** None stated.	Residential SU.	Treatment / tertiary	**Therapy intervention** (n = 33) called ‘Fathers for Change’ (F4C) aiming to reduce SU, IPV, negative parenting and increase positive co-parenting. Fathers (and mother and child where appropriate) received individual therapy sessions delivered by masters-level clinicians with experience in residential SU. Sessions targeted the intersection between DVA, SU and child maltreatment and were based on Substance Abuse Domestic Violence CBT (SADV) and behavioural couples’ therapy. First part focused on encouraging and supporting father in abstinence of SU and DVA using motivational enhancement, discussing child development, own childhood experiences of DVA and SU and how DVA and SU can impact parenting, and developing skills in reflective functioning and emotional regulation to reduce hostility. Second part involved focus on parental communication and problem-solving. Third part involved focus on restorative parenting.	Delivered in residential SU over a period of 12 weeks (12 sessions, weekly) plus 4 booster sessions (frequency not stated); hosted within residential SU.	Active control (n = 29) received ‘Dads n Kids’ (DNK) intervention delivered by masters-level clinicians over a period of 12 weeks (12 sessions, weekly). Involved clinician helping with basic family needs through problem-solving and providing fathers with a choice of leaflets that provided fathers with education on things such as child development, parenting, nutrition, lifestyle etc. and psychoeducation around parenting.	**Authors describe as uni-directional**; IPV (DVA) and SU co-occur and can lead to psychosocial problems (MH) which can impact role as father. **Intervention treats as bi-directional**; providing integrated treatment for DVA and SU in combination.	**Post-intervention (around 16 weeks):** **DVA =** fathers’ psychological, verbal and physical perpetration measured using TLFB-SV. **MH =** fathers’ general MH measured using BSI GSI. **SU =** fathers’ general SU measured using TLFB. **Follow-up (3 months post-intervention):** Same as above but in addition, measure psychological and physical perpetration and victimisation using CTS2.
**32. Trevillion et al. (2020)** (reference number: [69])	South East London, UK 66% White	Pregnant women (aged 16+, ≤26 weeks gestation) meeting the criteria for major depressive disorder or mixed anxiety and depressive disorder on DSM-IV. **Exclusion criteria:** Receiving MH support (e.g., CBT, therapy, secondary MH service), unable to provide informed consent/follow, intervention due to language barriers, other current MH disorder including psychosis, eating disorder, BPD, PTSD, or experiencing suicidality.	NHS maternity units / referrals from related research study.	Treatment	**Therapy** intervention (n = 26) aiming to reduce depressive symptoms. Mothers received guided self-help sessions delivered by Psychological Wellbeing Practitioners (PWPs) who worked within IAPT. Involved working through a workbook which provided mothers with psychoeducation on antenatal depression, information on relationships and parenthood planning, and health/lifestyle. Also involved regular homework tasks.	Delivered in clinic and/or remotely (telephone) over a period of 6–8 weeks (9 sessions, frequency not stated); hosted within Improving Access to Psychological Therapies (IAPT) programme within NHS.	Usual care control (n = 27) (no further details given).	**Authors do not state.** **Intervention treats as uni-directional–MH focused**; targeting depression with the view this may also lead to potential changes in DVA and SU.	**Post-intervention (14 weeks post randomisation):** **DVA =** mothers’ physical, emotional, and harassment-related victimisation measured using CAS. **MH =** mothers’ depression and anxiety measured using EPDS, PHQ-9, and GAD-7, respectively. **SU =** mothers’ alcohol use measured using AUDIT-C. **Follow-up (3 months post-intervention):** Same as above.

**NB.** Green coloured cells indicate studies that have had, or report to have, combined positive impacts on two or more outcomes. ^1^ Olds et al. (2004) conducted a three-arm RCT examining the effectiveness of a home visiting intervention delivered by paraprofessionals (intervention group 1) and a home visiting intervention delivered by nurses (intervention group 2) as compared to a minimal care control group. Therefore, there are two separate entries for this study, one with the paraprofessional delivered intervention as the intervention group and one with the nurse delivered intervention as the intervention group. ^2^ For outcome data compare intervention to active control as intervention designed to outperform on key outcomes. ^3^Parents were mostly mothers (only 1 was father). ASI = Addiction Severity Index; ASSIST = Alcohol Smoking and Substance Involvement Screening Test; AUDIT-C = Alcohol use disorders identification test for consumption; BAI = Beck Anxiety Inventory; BDI = Beck Depression Inventory; BPD = Borderline personality disorder; BSI = Brief Symptom Inventory; CAS = Composite Abuse Scale; CES-D = Center for Epidemiologic Studies Depression Scale; CIDI = Composite International Diagnostic Interview; CTS = Conflict Tactics Scale; DAST = Drug Abuse Screening Test; DIS = Diagnostic Inventory Schedule; DVA = Domestic violence; EPDS = Edinburgh Postnatal Depression Scale; GAD-7 = Generalised Anxiety Disorder-7; GHQ = General Health Questionnaire; GSI = Global Severity Index; IPV = Intimate partner violence; MH = Mental ill-health; MHI-5 = Mental Health Index-5; NFP = Nurse Family Partnership; PHQ-9 = Patient Health Questionnaire-9; PTSD = Post traumatic stress disorder; SF- = Short Form-; SU = Substance misuse; TLFB = Timeline Follow Back Interview; TLFB-SV = Timeline Follow Back Interview-Spousal Violence; WEB = Women’s Experience of Battering Scale.

The included studies comprised a mixture of secondary prevention [80, 86], treatment of DVA, MH, or SU [69, 70, 77, 82, 87, 88, 90, 92, 93], and tertiary prevention [68, 78, 83, 85, 91, 97–99, 101, 105, 106] interventions, and many provided support at multiple preventive levels [63–67, 79, 81, 84, 89, 94, 95, 100, 102–104].

The studies varied in terms of the type of intervention delivered which we categorised as follows; home visiting or parenting [67, 80, 94, 97–99, 101–103, 105, 106], home visiting or parenting supplements [63, 68, 78, 79, 97], therapy [65, 69, 70, 77, 84–86, 88–91], multi-component [66, 82, 83, 87, 92], empowerment/advocacy [81, 93, 104], coping skills [64, 95], and brief alcohol interventions [100] (S1 Appendix). Across these intervention types, there were three main approaches to addressing DVA, MH, or SU including approaches which treated these issues as: 1) co-occurring, intervening with DVA, MH, and SU in separate, distinct ways using the same intervention component or separate components, and not addressing the relationship *between* these issues [63, 64, 67, 68, 78, 79, 85, 91, 92, 94, 95, 97–99, 102, 103, 106]; 2) uni-directional, intervening by focusing on one main issue (either DVA [89, 97], MH [69, 88], or SU [77, 80, 83, 87, 90, 100] and hypothesising that this will lead changes in the others or by targeting the relationship between issues in one direction; or 3) bi-directional, intervening concurrently using the same intervention component and addressing the relationships between two or more of DVA, MH, and/or SU [65, 66, 70, 81, 82, 84, 86, 93, 104].

Studies varied in the combination of outcomes measured with eight measuring DVA and MH [70, 78, 81, 86, 92, 93, 97, 104], four measuring DVA and SU [65, 83, 98, 99], 13 measuring MH and SU [64, 66, 67, 77, 85, 87–91, 94, 95, 100], and 12 measuring all three outcomes [63, 68, 69, 79, 80, 82, 84, 101–103, 105, 106]. Outcomes were measured post-intervention [63–70, 77–94, 97–101, 104–106] or follow-up ranging from 6 weeks to 16 years post-intervention [63, 64, 66–69, 80, 82–86, 88, 90, 91, 95, 102–106].

### Risk of bias

Table 5 reports quality appraisal, further details of which can be found in S1 Appendix. The overall risk of bias judgement for the majority of studies was either ‘some concerns’ or ‘high risk’ of bias. Common issues included the use of self-report measures for DVA, MH, and SU, which may be prone to bias given that participants were often aware of their group allocation (as this is unavoidable for RCTs of psychosocial interventions), and lack of a publicly available, pre-specified data-analysis plan, making it difficult to assess whether data analysis had been conducted as intended. Several studies also failed to account for missing outcome data or provided limited information on how this was completed (n = 19). Other issues included limited information on, or problems with, the randomisation process (n = 10), failure to use valid and reliable measures (particularly for SU outcomes for which many authors relied on single self-report questions over validated measures) (n = 9), baseline differences between groups that may indicate bias in the randomisation process (n = 8), deviations from intended group assignments due to trial context (n = 4), inappropriate analysis to examine effect of assignment to intervention (n = 2), and bias in selection of reported results (n = 1).

**Table 5:** Quality appraisal results of included studies using RoB2.

Study number and author	1. Randomisation process	1b. Identification and recruitment of participants cluster RCTs	2. Effect of assignment to intervention	3.Missing outcome data	4. Measurement of outcome	5. Selection of reported result	Overall risk of bias judgement
**1.** Cupples et al.	L	N/A	L	H	SC	SC	H
**2.** Duggan et al.	L	N/A	L	H	H	SC	H
**3.** Duggan et al.	SC	N/A	L	SC	H	SC	H
**4.** El-Mohandes et al.	L	N/A	L	L	SC	SC	SC
**5.** Fergusson et al.	L	N/A	L	L	H	SC	H
**6.** Fleming et al.	L	N/A	L	SC	L	SC	SC
**7.** Grigg	L	N/A	SC	H	SC	SC	H
**8.** Slesnick et al.	L	N/A	SC	L	SC	SC	SC
**9.** Jack et al.	SC	L	L	SC	SC	SC	SC
**10.** Jacobs et al.	L	N/A	L	SC	SC	SC	SC
**11.** Jones et al.	SC	N/A	SC	L	SC	SC	SC
**12.** Lam et al.	SC	N/A	L	L	SC	SC	SC
**13.** Lecroy et al.	H	N/A	SC	SC	H	SC	H
**14.** Luthar et al.	SC	N/A	L	H	SC	SC	H
**15.** McWhirter	SC	N/A	L	L	H	SC	H
**16.** Nagle	L	N/A	L	SC	SC	SC	SC
**17.** Olds et al.	L	N/A	L	SC	H	SC	H
**18.** Olds et al.	L	N/A	L	L	H	SC	H
**19.** Ondersma et al.	L	N/A	L	L	SC	SC	SC
**20.** Rotheram-Borus	SC	N/A	L	SC	H	SC	H
**21.** Rotheram-Borus	SC	N/A	L	L	H	SC	H
**22.** Rotheram-Borus	L	SC	L	SC	H	SC	H
**23.** Silovsky et al.	L	N/A	H	L	SC	SC	H
**24.** Wu and Slesnick	SC	N/A	L	L	SC	SC	SC
**25.** Stover	L	N/A	H	H	SC	H	H
**26.** Stover et al.	L	N/A	H	L	SC	SC	H
**27.** Suchman et al.	SC	N/A	L	L	L	SC	SC
**28.** Suchman et al.	SC	N/A	L	L	SC	SC	SC
**29.** Sullivan et al.	SC	N/A	L	SC	H	SC	H
**30.** Taft et al.	H	H	L	L	SC	L	H
**31.** Tiwari et al.	L	N/A	L	L	SC	SC	SC
**32.** Trevillion et al.	L	N/A	L	L	SC	L	L
**33.** Volpicelli et al.	SC	N/A	L	SC	SC	SC	H
**34.** Walkup et al.	L	N/A	SC	H	H	SC	H
**35.** Zlotnick et al.	SC	N/A	L	L	SC	SC	SC
**36.** Dinmohammadi et al.	L	N/A	H	H	SC	H	H
**37.** Skar et al.	L	N/A	H	L	SC	SC	H

**NB.** L = Low risk of bias; SC = Some concerns; H = High risk of bias.

### Data synthesis

**Fig 2:**
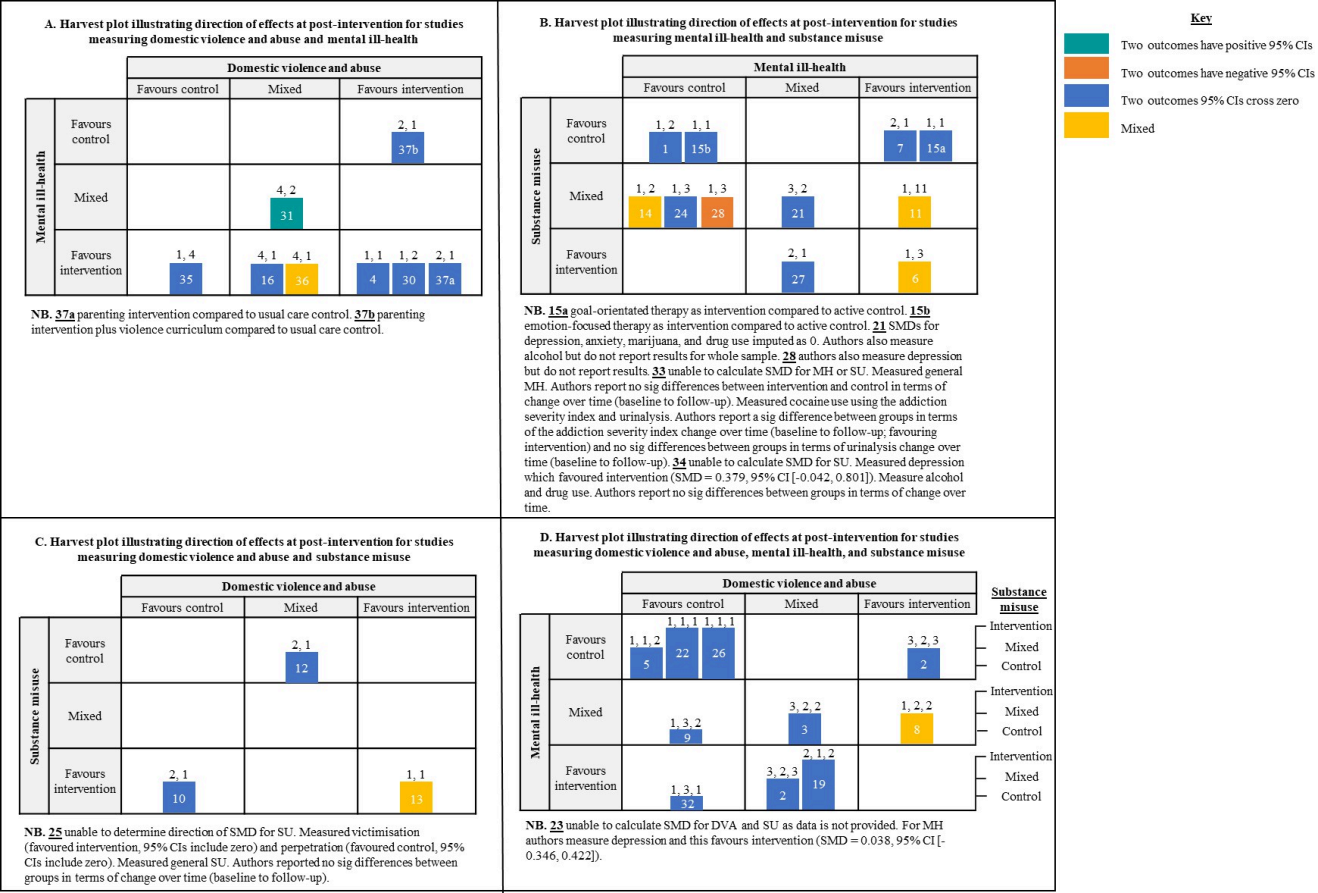
Direction of effects for combinations of DVA, MH, and SU outcomes at post-intervention. **Harvest plots A, B, and C: Bars** represent studies; **Placement of bars** represents direction of effect for DVA, MH, and/or SU outcomes; **Numbers above bars** represent number of outcome measures the categorisation is based on displayed in the following order where applicable: DVA, MH, SU; **Number in bars** represent the study number; **Colour** represents whether any of the SMDs 95% confidence intervals are positive, cross 0, or are negative (see key). **Harvest plot D** is same as previous but with the following addition: **Height of the bar** represents direction of effect for SU.

**Fig 3:**
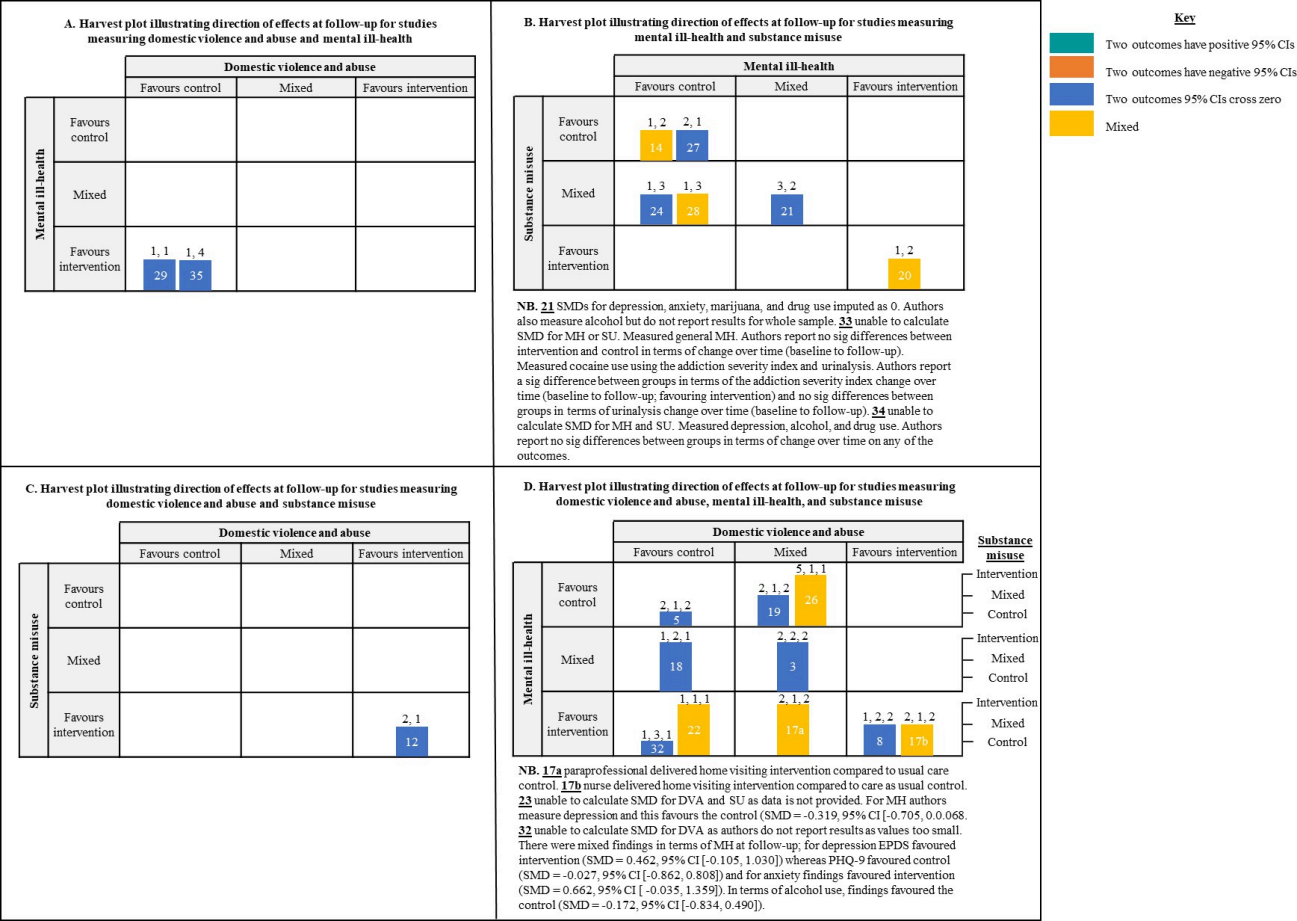
Direction of effects for combinations of DVA, MH, and SU outcomes at follow-up. **Harvest plots A, B, and C: Bars** represent studies; **Placement of bars** represents direction of effect for DVA, MH, and/or SU outcomes; **Numbers above bars** represent number of outcome measures the categorisation is based on displayed in the following order where applicable: DVA, MH, SU; **Number in bars** represent the study number; **Colour** represents whether any of the SMDs 95% confidence intervals are positive, cross 0, or are negative (see key). **Harvest plot D** is same as previous but with the following addition: **Height of the bar** represents direction of effect for SU.

Findings are presented and synthesised under four main headings corresponding to the combination of outcomes that studies measured: 1) DVA and MH; 2) DVA and SU; 3) MH and SU; and 4) DVA, MH, and SU. Figs 2 and 3 summarise the direction of effects for DVA, MH, and SU outcomes within each of the 37 studies using calculated SMDs and 95% CIs. Where we have been unable to calculate SMDs and 95% CIs, findings are reported narratively. Tables containing all SMDs and 95% CIs and harvest plots illustrating the direction of effect for sub-categories of DVA, MH, and SU can be found in S1 Appendix.

#### Domestic violence and abuse and mental ill-health

Eight studies measured a combination of DVA and MH (Table 1, Figs 2A and 3A). Three examined advocacy/empowerment interventions for mothers who had either experienced intimate partner violence (IPV) [93, 104] or were identified as psychologically distressed or had experienced IPV [81]. The remaining five studies involved a multicomponent intervention for mothers experiencing IPV and/or depression [92], a home-visiting supplement for first-time pregnant women living in poverty [78], a parenting intervention and parenting intervention supplement for low-income parents living in areas characterised with high levels of violence [97], and therapy interventions for low-income pregnant women who were at risk of MH due to experiencing IPV [86] and pregnant women experiencing minor or moderate DVA [70].

One study examining a culturally informed empowerment intervention delivered as a one-off session demonstrated a positive impact on combined outcomes of DVA and MH. Tiwari et al. [93] found mothers in the intervention group demonstrated better outcomes in terms of psychological abuse (SMD = 0.47, 95% CI [0.08–0.85]), minor physical abuse (SMD = 0.47, 95% CI [0.09–0.86]), and depression (SMD = 0.75, 95% CI [0.26–1.24]) at post-intervention as compared to a care as usual control group. However, the intervention group fared less favourably on a measure of general MH (SMD = -0.54, 95% CI [-0.15 –-0.93]) and there was no impact on more severe forms of physical violence or sexual abuse. The authors did not assess outcomes at follow-up. This study was rated as ‘some concerns’ in terms of risk of bias but this was mainly due to the use of self-reported measures for DVA and MH and lack of a pre-specified data-analysis plan.

One study also demonstrated a positive singular impact on DVA. Dinmohammadi et al. [70] examined the effectiveness of a solution-focused therapy intervention as compared to a usual care control. They found the intervention group demonstrated more positive outcomes in terms of psychological abuse at post-intervention (SMD = 0.63, 95% CI [0.189, 1.076]) however, this was not the case for other forms of violence or general MH. This study was rated as ‘high risk’ of bias.

The remaining studies did not demonstrate any impacts on our primary outcomes at post-intervention or follow-up when comparing the intervention to care as usual [81, 92, 97], a minimal support control [86], an active control [78], or a control group that was not described [104].

#### Domestic violence and abuse and substance misuse

Four studies measured a combination of DVA and SU (Table 2, Figs 2C and 3C). Two studies examined home-visiting interventions for young, first-time mothers [98] and new parents deemed at risk of child maltreatment [99]; one examined a multicomponent intervention for fathers who had been diagnosed with alcohol abuse/dependence and were voluntarily entering SU treatment [83]; and one examined a therapy intervention for fathers experiencing SU and DVA perpetration [65].

None of the studies demonstrated combined impacts on DVA and SU. However, two studies examining home-visiting interventions demonstrated impacts on singular outcomes. Jacobs et al. [98] found the intervention group demonstrated more positive outcomes in terms of marijuana use compared to the minimal-care control condition at post-intervention (SMD = 0.17, 95% CI [0.02, 0.32]) and LeCroy et al. [99] found intervention group mothers reported less physical violence victimisation as compared to the minimal-care control group at post-intervention (SMD = 1.13, 95% CI [0.80, 1.45]). The former was rated as ‘some concerns’, and the latter ‘high risk’, of bias. The remaining two studies did not demonstrate any differences between intervention and active control groups in terms of DVA and SU at post-intervention [83] and follow-up [65].

#### Mental ill-health and substance misuse

Fourteen studies examined a combination of MH and SU outcomes (Table 3, Figs 2B and 3B). Seven focused on therapy interventions targeting fathers experiencing alcohol misuse [77], mothers who were diagnosed with heroin addiction [88], mothers who had experienced IPV and were living in a family homeless shelter [89], mothers with SU disorder and reported parenting problems [90], and mothers enrolled in outpatient SU services [85, 91]. Two studies examined multicomponent interventions for men using opioids with opioid-dependent pregnant partners [87] and mothers diagnosed with cocaine-dependency [66]. Two studies involved home-visiting interventions targeting mothers due to a range of risk factors [67, 94]. Two studies focused on coping-skills interventions for mothers/parents living with HIV [64, 95] and one study examined a brief intervention for mothers screened at risk of alcohol misuse [100].

No studies had combined positive impacts on MH and SU. However, one therapy intervention had combined negative impacts on these outcomes compared to an active control. In Suchman et al. [91], mothers receiving individual mentalisation-based therapy sessions demonstrated worse general MH (SMD = -1.51, 95% CI [-1.03, -1.98]) and greater heroin use (SMD = -0.67, 95% CI [-0.24, -1.10]) at post-intervention, compared to those receiving a manualised parenting education intervention. Although this result was maintained for general MH at follow-up (SMD = -1.26, 95% CI [-0.80, -1.72]), the intervention group fared better in terms of heroin (SMD = 1.00, 95% CI [0.55, 1.45]) and cocaine use (SMD = 1.03, 95% CI [0.58, 1.48]) at follow-up. This study was rated as ‘some concerns’ in terms of risk of bias.

Two other therapy intervention studies had mixed impacts on MH and SU compared to an active control. Although one study [87] found intervention mothers had better depression outcomes (SMD = 0.66, 95% CI [0.09, 1.23]) they fared worse in terms of heroin use (SMD = -1.78, 95% CI [-2.42, -1.14]), drug use (SMD = -1.64, 95% CI [-2.27, -1.02]), and alcohol use (any use SMD = -1.84, 95% CI [-2.48, -1.19]; intoxication SMD = -1.27, 95% CI [-1.87, -0.67]; composite SU SMD = -1.44, 95% CI = [-2.27, -1.02]) at post-intervention. Another study demonstrated that, although intervention mothers had better cocaine use outcomes at post-intervention (SMD = 0.54, 95% CI [0.19, 0.89]), this effect was lost at follow-up and mothers demonstrated worse depression outcomes (SMD = -0.38, 95% CI [-0.73, -0.03]) [88]. These studies were rated as ‘some concerns’ and ‘high risk’ of bias, respectively.

Three studies had a positive singular impact on SU outcomes. One study reported reductions in alcohol use at post-intervention as compared to a minimal control (mean number of drinks in previous 28 days SMD = 0.35, 95% CI [0.10, 0.61] and mean number of heavy drinking days SMD = 0.34, 95% CI [0.08, 0.60]) [100]. One reported a reduction in current general substance use as compared with care as usual (SMD = 0.50, 95% CI [0.01, 0.88]) [95]. One study reported reductions in cocaine use compared to an active control (authors report significant difference between groups in terms of change from baseline to follow-up favouring the intervention) [66]. The first of these studies was rated as ‘low risk’ in terms of risk of bias for MH outcomes but ‘some concerns’ in terms of risk of bias for SU outcomes [100]. The other two were rated overall as either ‘some concerns’ or ‘high risk’ of bias [66, 95].

The remaining studies did not have an impact on any of our primary outcomes compared to usual care [94], an active control [67, 77, 85, 89–91], or a control group that was not described [64].

#### Domestic violence and abuse, mental ill-health, and substance misuse

Twelve studies measured a combination of parental DVA, MH, and SU outcomes (Table 4, Figs 2D and 3D). The majority of these studies examined home-visiting interventions [80, 101–103, 105, 106] or home-visiting supplements [63, 68, 79] targeting mothers/parents for a range of demographic or contextual risk factors. Two studies focused on a therapy intervention for fathers experiencing SU and DVA perpetration [84] and for pregnant women meeting criteria for depression [69]. One study examined a multicomponent intervention for homeless mothers experiencing SU [82].

No studies demonstrated a combined impact on two or more of parental DVA, MH, or SU outcomes and only four had an impact on one of these outcomes. Two home-visiting studies demonstrated positive singular impacts on outcomes. One found improved general MH in a paraprofessional delivered home-visiting intervention (SMD = 0.21, 95% CI [0.02, 0.40]) and less physical victimisation in a nurse delivered home-visiting intervention (past six-month physical victimisation SMD = 0.42, 95% CI [0.05, 0.78]; past 12-month physical victimisation SMD = 0.28, 95% CI [0.01, 0.55]) as compared to a minimal-care control at follow-up [103]. Another found improved depression in the intervention group as compared to a care as usual control at follow-up (SMD = 0.14, 95% CI [0.01, 0.27]) [80]. These studies were rated as ‘some concerns’ [103] and ‘high risk’ [80] of bias. One multi-component intervention demonstrated positive outcomes in terms of alcohol use compared to an active control group at post-intervention (SMD = 0.56, 95% CI [0.04, 1.07]) but this was not maintained at follow-up [82]. One study examining a therapy intervention reported that fathers in the active control group receiving a manualised intervention involving clinician support and parenting education demonstrated greater reductions in physical violence perpetration (SMD = -0.62, 95% CI [-0.67, -0.11]) and victimisation (SMD = -0.810, 95% CI [-1.33, -0.29]) compared to the intervention group at three months follow-up [84]. However, this study was rated as ‘high risk’ of bias.

None of the other studies had an impact on DVA, MH, or SU outcomes at post-intervention or follow-up as compared to care as usual [69, 101, 106], minimal control [102], active control [63, 68, 79], or a control group that was not described [105]. One of these studies was rated as ‘low risk’ of bias [69], whereas the others were rated as either ‘some concerns’ [63, 79] or ‘high risk’ [68, 101, 102, 105, 106] of bias.

## Discussion

Commissioners and service providers are seeking better ways to prevent and respond to families with multiple and complex needs, including clustered parental DVA, MH, and SU. However, there remains a lack of evidence-based guidance for them to draw upon. To address this gap, we synthesised evidence from 37 studies to examine the effectiveness of family focused interventions targeting DVA, MH or SU. Our aim was to examine whether interventions are effective in addressing these outcomes in combination and, if so, to identify the current ‘best bet’ preventive family focused interventions.

Of the 37 studies we examined, no studies demonstrated combined positive impacts on all three of these outcomes within the timeframes examined and only one intervention had a combined positive impact on two of these outcomes. This study targeted DVA and MH, used a brief, one-off empowerment-based approach, and treated these issues as bi-directional, offering concurrent support for DVA and MH, recognising and addressing the relationships between them. Studies targeting MH and SU often demonstrated more mixed or negative impacts on outcomes which could be related to the type of intervention delivered [128], or perhaps because they remove a trauma coping mechanism without providing additional support to manage this. There were also several studies that demonstrated singular impacts on outcomes despite attempting to tackle DVA, MH, and SU in combination. Most of the studies were rated as either ‘some concerns’ or ‘high risk’ of bias, reducing our confidence in any positive findings.

Most interventions either implicitly or explicitly conceived the relationship between DVA, MH, and SU as co-occurring, providing support for each issue in separate, distinct ways without addressing the relationship between them, or were uni-directional, providing support targeting one main issue in the expectation that this will lead to changes in the others. However, these conceptual approaches resulted in interventions that appeared to be largely ineffective in addressing combined/clustered parental DVA, MH, and SU at post-intervention and follow-up. Uni-directional interventions tended to use therapy-based approaches to target SU as the primary issue (and MH as secondary) but failed to demonstrate any combined, or consistent singular, impacts across outcomes. Interventions that conceived these issues as co-occurring were more common; most utilised home visiting interventions or supplements, with an identification and onward referral approach for supporting these issues. However, these interventions too demonstrated no combined, and very few singular, impacts on outcomes. The commissioners and service providers we spoke to raised additional concerns with this approach indicating that, even where successful identification occurs, families are likely to be referred to existing siloed services which may present multiple barriers to access [18, 35, 36], providing another opportunity for intervention failure. Our findings here add to concerns already raised about the usefulness of uni-directional approaches within the context of complex behaviour change interventions [129] and question whether the ‘integrated’ nature of co-occurring approaches are integrated enough.

Integrated approaches to addressing co-occurring and clustering issues can take many forms and pose challenges for interventions targeting multiple behaviours [130]. They can involve increasing communication, collaboration, or co-ordination between services, organisations, and/or systems; co-location of services addressing interrelated needs; introduction of multidisciplinary teams; or equipping practitioners with knowledge and understanding of co-occurring and clustered issues [131–133]. We suggest that integrated approaches for parental DVA, MH, and SU may need to go beyond these measures to concurrently recognise and address the *bi-directional* and complex nature of these issues [10–16], while also addressing the underlying risk factors that may give rise to, or exacerbate, them. Other researchers have highlighted the importance of adopting such an approach, particularly when working with women experiencing DVA, MH, and SU [134–136]. In our review, the only study that demonstrated combined impacts on two outcomes (DVA and MH) conceived the relationship between these issues as bi-directional; providing concurrent support for DVA and MH through a culturally informed, empowerment intervention, empathetic-understanding component, and social support from the person delivering the intervention [93]. This provides some support for the use of such an approach, particularly where women are experiencing DVA. However, we also found interventions implicitly conceiving these issues as bi-directional that had very few or mixed impacts. These interventions were less likely to be explicitly culturally informed and/or adapted, and focused on different cultural groups, which may, in part, provide some explanation for differing impacts. Tiwari et al. [93] targeted the specific relationship between psychological abuse and psychological well-being considering the influences of cultural perceptions and norms. This specificity in the intervention target may have been important for its effectiveness however, the scarcity of evidence precludes us from drawing any strong conclusions.

Integration also applies to joined up working between adult and child services. Parental DVA, MH, and SU are intergenerational issues; within the family context they not only impact mothers and fathers [17], but also parenting capacity [23, 24] and children [24–27, 29, 30]. Whole family approaches that work with the mothers, fathers and children have been advocated to effectively address parental DVA, MH, and SU and the negative impact these issues can have on children [see 137 for example]. Our review sought family focused approaches that provide integrated support for parents and children, recognising the interrelated nature of their needs [138]. Of the 37 studies included in our review, only eight studies attempted to directly work with children alongside their parents. These studies involved the child in family therapy [90], parent and child groups and/or child-only groups focused on strengths-based advocacy [104], empowerment and goal-setting [89], coping skills [64, 95], or restorative parent sessions [65, 84]. Furthermore, the majority targeted mothers alone and only six studies explicitly involved working with the father. In three studies, work with fathers occurred in parallel with working with the mother [77, 83, 87] and, in three, the mother and child [65, 84, 90]. Where studies did involve the father, this was where fathers already displayed established DVA perpetration or SU. No studies explicitly targeted fathers due to MH or for risk factors related to DVA, MH, or SU (as they did mothers). These findings echo that of previous work which highlighted that practices working at the intersection of DVA, MH, and SU tend to ‘converge’ on mothers, which may reinforce victim-blaming, monitoring of mothers, and invisibility of abusers [139]. Although working with mothers is important, evidence suggests that paternal DVA, MH, and SU also adversely impacts child outcomes by negatively impacting mother-child relationships [140] or through paternal depression and diminished ability to co-parent in the case of DVA perpetration [141]. We did not identify any clear patterns in terms of parental outcomes and *who* the intervention targeted. To effectively address DVA, MH, and SU, we believe one of the first steps is to redesign family focused interventions in this space to recognise and work with the whole family.

Although there were few studies in our review that demonstrated combined or singular impacts on outcomes, it is important to note that those that did were more likely to compare the intervention group with a ‘care as usual’ or ‘minimal care’ control group as opposed to an active control. However, reporting of what ‘care as usual’ involved varied across studies making it difficult to assess the generalisability of the results to other contexts/countries. Careful documentation and reporting of the ‘usual care’ control groups receive is important given that this can vary considerably across different locations and time [142], helps readers interpret the applicability of findings, and is important for the interpretation of null findings [143]. Furthermore, fifteen studies included in our review used ‘active’ control groups and some of these demonstrated reductions in both groups over time (e.g., [89]); this could reflect regression to the mean [144] or be indicative of both intervention and control having positive impacts on outcomes, making these active control interventions worth further exploration.

### Limitations of individual studies

Studies included in our review demonstrated several common limitations. Firstly, outcome measurements for DVA, MH, and SU varied greatly across studies and, in the case of SU, studies often relied on their own scales or single questions rather than measures with established reliability and validity. Although this may have been done to reduce participant burden [129], it makes synthesis across studies difficult and lowers our confidence in the findings. We encourage future authors to utilise resources on core outcomes (see https://www.comet-initiative.org/) alongside PPIE to guide selection of appropriate, reliable, and valid DVA, MH, and SU outcomes [145]. Secondly, many studies lacked detailed information on theory, behaviour-change techniques, and mechanisms of change underpinning the interventions. This is essential to allow for an in-depth synthesis of complex interventions that is useful for decision makers [145]. However, in the context of our review, even where these were reported, theories of change were quite distinct, and impacts on outcomes sporadic, making a synthesised family focused model for clustered risk unlikely at this stage. Thirdly, several studies failed to provide adequate data to allow us to calculate SMDs and CIs for post-intervention and follow-up timepoints. Fourthly, most studies were rated as either ‘some concerns’ or ‘high risk’ of bias when assessing quality using the RoB2 (see [69] for exception).

### Strengths and limitations of the review

Our review is the first to examine the effectiveness of preventive family focused interventions in addressing a combination of parental DVA, MH, and SU. Its strengths include having a publicly available pre-defined protocol, examining the high-quality evidence (RCTs), a comprehensive search strategy that was developed in consultation with information specialists, broad inclusion/exclusion criteria, and being informed by PPIE work with those with lived experience, service providers, and commissioners in terms of the design, conduct, and interpretation of the results. It is unique in synthesising evidence on multiple outcomes in this space and adds to previous literature that has done this in relation to other risk behaviours (e.g., [146]).

As well as considering its strengths, our review should also be considered within the context of its limitations. Firstly, our searches focused specifically on RCTs to ensure it was feasible and captured the highest quality effectiveness evidence available [147]. Consequently, our review does not include other useful forms of evidence (e.g., natural experiments such as quasi-experimental trials, ‘before-and-after’ trials, or qualitative studies) which may be preferable on pragmatic or ethical grounds [148, 149]. Future work should consider what we can also learn from the wider evidence base; we are aware of one research team already beginning to explore this area [150]. Secondly, the parameters of our search mean that there are interventions that could be effective in preventing and addressing the combination of DVA, MH, and SU that did not meet our inclusion criteria, for example of targeting outcomes in parents (rather adults generally). There is a growing body of evidence on multisystemic therapy in emerging adults which has demonstrated some positive impacts in terms of violence, anti-social behaviour, MH, and SU in adolescents [151]. Furthermore, RCTs that measure proxies of parental DVA, MH, and/or SU (rather than these outcomes specifically) would not have been identified by our review but may be a useful avenue for future research, particularly in the context of secondary prevention. Finally, our search strategy (designed for both the current review and a larger, intervention components analysis) resulted in a large number of search results. Although we consider our comprehensive search strategy a strength of the review, the additional time and resource needed to screen titles and abstracts, and consequent opportunity for error, is a notable limitation. Using a combination of automated filters and second screening is a useful way to make screening for such reviews more manageable and accurate, and we encourage others to adopt a similar approach when undertaking reviews on combined impacts.

Our review focuses on interventions to address one specific ACE cluster: parental DVA, MH, and SU. We examined interventions addressing this cluster due to the increased policy and practice focus on these three public health issues [32, 36, 37], the adverse impacts they can have on both parents and children [17, 24, 29, 30], and calls from commissioners, service providers and academics to advance our knowledge of interventions to address ACE clusters [152, 153]. There are undoubtedly other ACE clusters that are important to consider, and other ACEs that could be considered in combination with parental DVA, MH, and SU. For example, recent work has demonstrated that poverty is an important risk factor influencing the expression of parental DVA, MH, and SU [154] and that upstream approaches to supporting families (e.g., financial support, housing, income supplementation) offer promise in reducing children’s exposure to various ACEs [155]. Although we did not include poverty as an intervention target in our review, several studies targeted parents due to low-income and the majority of study samples displayed above average levels of low socio-economic status based on indicators of income, education, and unemployment. Notably, the only study demonstrating combined impacts on two outcomes [93] was one of the few studies which included a sample that was not characterised by low SES across indicators. While being mindful not to make interventions so multifaceted and complex as to be implausible, considering poverty alongside DVA, MH, and SU is likely to be important target for future interventions.

### Future research directions

Family focused interventions included in our review not only aimed to address parental DVA, MH, and SU, but also the negative impact these issues might have on the child. They did this by working directly with the mother (and in some cases the father) with the view this might indirectly impact the child or, in a few cases, by working directly with both the mother (and in some cases the father) and the child/ren [138]. In addition, some studies collected direct and indirect impacts on the children’s emotional and behavioural development [64, 67, 80, 82, 83, 88–90, 94, 95, 98, 101–106]. Despite the limited positive impacts on parental outcomes, these studies may offer important insights into key intervention types and mechanisms that have the potential to ameliorate child outcomes in the context of parental DVA, MH, and SU. Furthermore, future work may also want to explore whether family focused interventions might be useful in reducing the impact DVA, MH, and SU may have on other outcomes (e.g., family functioning, parent-child interactions); outcomes many of the included studies measured.

Given the dearth of effective, targeted, family focused interventions for parental DVA, MH, and SU, we also now need to turn our attention to what an effective ‘best bet’ intervention might look like for families at risk of, or experiencing, parental DVA, MH, and/or SU. One way we can do this is examining whether there are *shared* intervention components that have *common* impacts across these three public health issues, and those which may have iatrogenic effects. We are currently undertaking work to address this, which will help highlight key intervention components that are more/less helpful and guide the development of future interventions and provision of current services in this space. An exploration of the wider literature, e.g., process evaluations, might also elucidate whether there are commonalities in why studies in our review largely failed to demonstrate combined impacts on parental outcomes. For example, issues around intervention fidelity and family engagement were occasionally cited by study authors to, in part, explain null findings and could be an important focus for future work. Furthermore, although we did not restrict our search to studies from high-income countries, we identified very few studies from low- and middle-income countries. Others have identified a similar gap in the literature [156], and we know of one review which will explore this in more depth; reviewing interventions addressing DVA, MH, and/or SU in low- and middle-income countries, specifically [157]. Future work should seek to fill this gap in the evidence-base, taking into account lessons that can be learnt from high-income countries and their implementation of integrated interventions [158].

## Conclusion

Parental DVA, MH, and SU are three public health issues that not only co-occur but cluster and can create an intergenerational cycle of disadvantage exacerbated by the historically siloed nature of service provision. Our systematic review highlights the distinct lack of family focused interventions that have demonstrated combined impacts in this space. This is likely to be due to the design and function of family focused interventions which often fail to address whole family needs and the interrelationships between DVA, MH, and SU. Academics are encouraged to join forces with colleagues in policy and practice and those with lived experience to explore new ways to target these clustering issues, recognise and address their bi-directional nature, and better support families most at risk.

## Supporting information

S1 Appendix Supporting information. All supporting information tables and figures.
(DOCX)

S2 Appendix Outcome data. List of included studies and extracted outcome data.
(XLSX)
