# Supplementary material for: Family focused interventions that address parental domestic violence and abuse, mental ill-health, and substance misuse in combination: A systematic review
Source: PLoS One. 2022 Jul 29;17(7):e0270894. doi: 10.1371/journal.pone.0270894 (PMC9337671; doi:10.1371/journal.pone.0270894)
Supplement: S1 Appendix — All supporting information tables and figures. (DOCX) [file pone.0270894.s001.docx]

**Supporting Information**

**Appendix A – Key terms and definitions**

Domestic violence and abuse

Domestic violence and abuse (DVA) refers to *“any incident or pattern of incidents of controlling, coercive, threatening behaviour, violence or abuse between those aged 16 or over who are, or have been, intimate partners or family members, regardless of gender or sexuality. The abuse can encompass, but is not limited to, psychological, physical, sexual, financial and emotional”* [1]. This definition was expanded to recognise DVA is a gendered issue that disproportionately affects women and girls.

Mental ill-health

Mental ill-health (MH) refers to *common mental health disorders* including depression, anxiety, post-traumatic stress disorder (PTSD), panic disorder and obsessive-compulsive disorder [2].

Substance misuse

Substance misuse (SU) refers to use of illicit psychoactive drugs or alcohol that is not in line with medical use, despite the negative impact this has on the individual or their family in relation to psychological, sociological, financial or physiological factors [3, 4]. For the purposes of this review, this definition will not include tobacco as this is a drug which is more likely to cause harm later on in life rather than in the short-term [5].

Adverse Childhood Experiences

Adverse Childhood Experiences (ACEs) refer to a range of adverse events that may occur during childhood including, but not limited to; child abuse (physical, psychological, or sexual), child neglect (physical or psychological), parental separation/divorce, incarceration, parental domestic violence and abuse, parental mental ill-health, and parental substance misuse [6-8], and structural ACEs such as racism [9] and poverty [10]. Numerous studies have demonstrated a count-response relationship between ACEs and adverse health, social, emotional, and economic outcomes [11-13]. The more ACEs a child experiences, the greater the odds of negative outcomes during childhood including asthma, allergies, headaches, poor digestion, and secondary school absenteeism [14], and adulthood including chronic health conditions, unemployment, violence, mental ill-health, and substance misuse [12, 13]. This pervasive finding has resulted in greater recognition of ACEs worldwide, with practitioners and academics alike calling for increased efforts to prevent and respond to ACEs and thus, the negative impact they can have across the life course [11-13].

**Appendix B – PRISMA checklists**


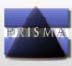
**PRISMA 2020 for Abstracts Checklist**

| **Section and Topic** | **Item #** | **Checklist item** | **Reported (Yes/No)** |
| --- | --- | --- | --- |
| **TITLE** | | |  |
| Title | 1 | Identify the report as a systematic review. | Yes |
| **BACKGROUND** | | |  |
| Objectives | 2 | Provide an explicit statement of the main objective(s) or question(s) the review addresses. | Yes |
| **METHODS** | | |  |
| Eligibility criteria | 3 | Specify the inclusion and exclusion criteria for the review. | Yes |
| Information sources | 4 | Specify the information sources (e.g. databases, registers) used to identify studies and the date when each was last searched. | Yes |
| Risk of bias | 5 | Specify the methods used to assess risk of bias in the included studies. | Yes |
| Synthesis of results | 6 | Specify the methods used to present and synthesise results. | Yes |
| **RESULTS** | | |  |
| Included studies | 7 | Give the total number of included studies and participants and summarise relevant characteristics of studies. | Yes |
| Synthesis of results | 8 | Present results for main outcomes, preferably indicating the number of included studies and participants for each. If meta-analysis was done, report the summary estimate and confidence/credible interval. If comparing groups, indicate the direction of the effect (i.e. which group is favoured). | Yes |
| **DISCUSSION** | | |  |
| Limitations of evidence | 9 | Provide a brief summary of the limitations of the evidence included in the review (e.g. study risk of bias, inconsistency and imprecision). | Yes |
| Interpretation | 10 | Provide a general interpretation of the results and important implications. | Yes |
| **OTHER** | | |  |
| Funding | 11 | Specify the primary source of funding for the review. | Yes |
| Registration | 12 | Provide the register name and registration number. | Yes |

*From:*  Page MJ, McKenzie JE, Bossuyt PM, Boutron I, Hoffmann TC, Mulrow CD, et al. The PRISMA 2020 statement: an updated guideline for reporting systematic reviews. BMJ 2021;372:n71. doi: 10.1136/bmj.n71

For more information, visit: <http://www.prisma-statement.org/>


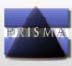
**PRISMA 2020 Checklist**

| **Section and Topic** | **Item #** | **Checklist item** | **Location where item is reported** |
| --- | --- | --- | --- |
| **TITLE** | | |  |
| Title | 1 | Identify the report as a systematic review. | 1 |
| **ABSTRACT** | | |  |
| Abstract | 2 | See the PRISMA 2020 for Abstracts checklist. | 2 |
| **INTRODUCTION** | | |  |
| Rationale | 3 | Describe the rationale for the review in the context of existing knowledge. | 3-4 |
| Objectives | 4 | Provide an explicit statement of the objective(s) or question(s) the review addresses. | 4 |
| **METHODS** | | |  |
| Eligibility criteria | 5 | Specify the inclusion and exclusion criteria for the review and how studies were grouped for the syntheses. | 5 and 9, Appendix B |
| Information sources | 6 | Specify all databases, registers, websites, organisations, reference lists and other sources searched or consulted to identify studies. Specify the date when each source was last searched or consulted. | 5-6 |
| Search strategy | 7 | Present the full search strategies for all databases, registers and websites, including any filters and limits used. | Appendix C |
| Selection process | 8 | Specify the methods used to decide whether a study met the inclusion criteria of the review, including how many reviewers screened each record and each report retrieved, whether they worked independently, and if applicable, details of automation tools used in the process. | 6 |
| Data collection process | 9 | Specify the methods used to collect data from reports, including how many reviewers collected data from each report, whether they worked independently, any processes for obtaining or confirming data from study investigators, and if applicable, details of automation tools used in the process. | 6-7 |
| Data items | 10a | List and define all outcomes for which data were sought. Specify whether all results that were compatible with each outcome domain in each study were sought (e.g. for all measures, time points, analyses), and if not, the methods used to decide which results to collect. | 6-9 |
|  | 10b | List and define all other variables for which data were sought (e.g. participant and intervention characteristics, funding sources). Describe any assumptions made about any missing or unclear information. | 6-9 |
| Study risk of bias assessment | 11 | Specify the methods used to assess risk of bias in the included studies, including details of the tool(s) used, how many reviewers assessed each study and whether they worked independently, and if applicable, details of automation tools used in the process. | 7 |
| Effect measures | 12 | Specify for each outcome the effect measure(s) (e.g. risk ratio, mean difference) used in the synthesis or presentation of results. | 7-9 |
| Synthesis methods | 13a | Describe the processes used to decide which studies were eligible for each synthesis (e.g. tabulating the study intervention characteristics and comparing against the planned groups for each synthesis (item #5)). | 7-9 |
|  | 13b | Describe any methods required to prepare the data for presentation or synthesis, such as handling of missing summary statistics, or data conversions. | 7-9 |
|  | 13c | Describe any methods used to tabulate or visually display results of individual studies and syntheses. | 7-9 |
|  | 13d | Describe any methods used to synthesize results and provide a rationale for the choice(s). If meta-analysis was performed, describe the model(s), method(s) to identify the presence and extent of statistical heterogeneity, and software package(s) used. | 7-9 |
|  | 13e | Describe any methods used to explore possible causes of heterogeneity among study results (e.g. subgroup analysis, meta-regression). | 7-9 |
|  | 13f | Describe any sensitivity analyses conducted to assess robustness of the synthesized results. | N/A |
| Reporting bias assessment | 14 | Describe any methods used to assess risk of bias due to missing results in a synthesis (arising from reporting biases). | 7-9 |
| Certainty assessment | 15 | Describe any methods used to assess certainty (or confidence) in the body of evidence for an outcome. | 7-9 |
| **RESULTS** | | |  |
| Study selection | 16a | Describe the results of the search and selection process, from the number of records identified in the search to the number of studies included in the review, ideally using a flow diagram. | 11 |
|  | 16b | Cite studies that might appear to meet the inclusion criteria, but which were excluded, and explain why they were excluded. | N/A |
| Study characteristics | 17 | Cite each included study and present its characteristics. | 14-52 |
| Risk of bias in studies | 18 | Present assessments of risk of bias for each included study. | 53-54 |
| Results of individual studies | 19 | For all outcomes, present, for each study: (a) summary statistics for each group (where appropriate) and (b) an effect estimate and its precision (e.g. confidence/credible interval), ideally using structured tables or plots. | Appendix H |
| Results of syntheses | 20a | For each synthesis, briefly summarise the characteristics and risk of bias among contributing studies. | 11-13, 57-62 |
|  | 20b | Present results of all statistical syntheses conducted. If meta-analysis was done, present for each the summary estimate and its precision (e.g. confidence/credible interval) and measures of statistical heterogeneity. If comparing groups, describe the direction of the effect. | Appendix H |
|  | 20c | Present results of all investigations of possible causes of heterogeneity among study results. | 57-62 |
|  | 20d | Present results of all sensitivity analyses conducted to assess the robustness of the synthesized results. | 57-62 |
| Reporting biases | 21 | Present assessments of risk of bias due to missing results (arising from reporting biases) for each synthesis assessed. | 53-54 |
| Certainty of evidence | 22 | Present assessments of certainty (or confidence) in the body of evidence for each outcome assessed. | Appendix H |
| **DISCUSSION** | | |  |
| Discussion | 23a | Provide a general interpretation of the results in the context of other evidence. | 62-70 |
|  | 23b | Discuss any limitations of the evidence included in the review. | 66-67 |
|  | 23c | Discuss any limitations of the review processes used. | 67-69 |
|  | 23d | Discuss implications of the results for practice, policy, and future research. | 69-70 |
| **OTHER INFORMATION** | | |  |
| Registration and protocol | 24a | Provide registration information for the review, including register name and registration number, or state that the review was not registered. | 4 |
|  | 24b | Indicate where the review protocol can be accessed, or state that a protocol was not prepared. | 4 |
|  | 24c | Describe and explain any amendments to information provided at registration or in the protocol. | N/A |
| Support | 25 | Describe sources of financial or non-financial support for the review, and the role of the funders or sponsors in the review. | 71 |
| Competing interests | 26 | Declare any competing interests of review authors. | Declared in application |
| Availability of data, code and other materials | 27 | Report which of the following are publicly available and where they can be found: template data collection forms; data extracted from included studies; data used for all analyses; analytic code; any other materials used in the review. | See appendices |

**Appendix C – Inclusion/Exclusion criteria**

| **Category** | **Include** | **Exclude** |
| --- | --- | --- |
| **Population** | - Majority must be parents/carers (aged 16 or above) of one or more children (under the age of 18)^*^ who are at risk of^+^, or experiencing, one or more of the following:  1. Domestic violence and abuse 2. Mental-ill health 3. Substance misuse   AND/OR the children (under the age of 18) in their care.  ^*^Taking a life course approach, this may include pregnant women.  ^+^Parents/carers ‘at risk’ of these issues include young parents, first-time parents, low-income parents, or those who are explicitly described as ‘at risk’ due to some other factor. | - Older parents/carers aged 65 years or over. - Teenage parents/carers under the age of 16 years. - Parents/carers who have been diagnosed with severe mental health disorders (e.g. bipolar disorder, psychosis, schizophrenia). - Women who are not yet pregnant. - Children have already been removed from the care of both parents/caregivers. - Parents/carers targeted due to the fact their child has a long-term health condition (e.g. cancer, autism, preterm infant). |
| **Intervention** | - Primary aim is to prevent/treat parental domestic violence, mental ill-health, and/or substance misuse **and/or** the negative impact these issues might have on children in their care. This includes interventions aiming to prevent/reduce child maltreatment as long as it does this by targeting parental domestic violence, mental ill-health, and/or substance misuse. - Family focused which is defined as; *‘any intervention that includes a parent-child component. This may include a component focusing on parents’ skills, parenting capacity, parent-child relationship, working with the parent in order to improve child outcomes (in the context of parental risk of, or experiences of, domestic violence, mental ill-health, and/or substance misuse), or working with the child alone to reduce the impact of parental domestic violence, mental ill-health and/or substance misuse. Family focused interventions may involve the parent/carer and/or the child(ren) in their care. In addition, they may also involve the wider family network (e.g. partners, ex-partners, grandparents, and friends). Where this is the case, the intervention may be delivered to family members in a group, individually (separately, but simultaneously), or delivered using a combination of the two. Interventions may be delivered by professionals and non-professionals alike.’* Interventions solely targeting parents (where the whole sample are described as parents) are assumed to be ‘family focused’. - Psychosocial in nature. Psychosocial interventions include those that have a predominately psychological and/or social focus. However, they may also include other additional educational, pharmaceutical, economic, or legal components. - Prevention/treatment focused. This may include:  1. Family focused interventions aiming to prevent parental domestic violence, mental ill-health, and/or substance misuse from happening or getting worse by targeting parents/carers at risk of domestic violence, mental ill-health, and/or substance misuse or experiencing early signs of any one or more of these three issues (secondary prevention) 2. Family focused interventions aiming to prevent parental domestic violence, mental ill-health, and/or substance misuse from having a negative impact on the children in their care (tertiary prevention) 3. Family focused interventions aiming to treat current parental domestic violence, mental ill-health, and/or substance misuse (treatment) | - Primary aim is to prevent/treat:   - Anxiety around a singular, specific event (e.g. pre-operative interventions, anxiety around labour).   - Parenting stress (where this has not been operationalised as depression/anxiety and has not been linked with mental ill-health constructs).   - Child related behaviour (e.g. child conduct disorder) but which is not in the context of parental domestic violence, mental ill-health, and/or substance misuse.   - ‘General’ trauma, where this trauma has not been specifically linked to parental domestic violence, mental ill-health and/or substance misuse (e.g. may include trauma related to gang violence, life threatening accidents). - Not family focused (i.e. intervention is not solely targeting parents and/or children in their care / does not have a clear family focused element). - Predominately pharmaceutical in nature - Predominately physical in nature (e.g. exercise interventions, skin to skin contact interventions including kangaroo care) - Primary prevention/universal interventions targeting the general population who are not known/explicitly stated to be at any increased risk of domestic violence, mental ill-health and/or substance misuse. |
| **Comparator** | - Any control group. This could be a wait-list control, active control, normal control as long as this is part of an RCT (see criteria below). | - Any study that does not involve a control group. |
| **Outcomes** | - Measures **two or more** of the following quantitative outcomes:   - Inter-parental violence victimisation/perpetration – this may include physical, psychological, emotional, financial, or sexual violence.   - Parent/carer mental ill-health – this may include depression, anxiety, PTSD, OCD, panic disorder, or general mental ill-health.   - Parent/carer substance misuse – this may include alcohol or drug use. | - Do not report on **two or more** of the following outcomes; inter-parental violence victimisation/perpetration, parent/carer mental ill-health, and/or parent/carer substance misuse. - Outcomes such as relationship quality, risky behaviours etc. that are not specifically measuring the outcomes described opposite. - Qualitative outcomes. |
| **Study design** | - RCTs including fully powered RCTs or RCTs conducted as part of a feasibility/pilot study. Authors should explicitly state that the trial is an RCT or that the participants (or clusters) have been randomly allocated to intervention/active control/control groups. | - Non-RCTs. - Any studies described as ‘quasi-experimental’ or ‘quasi-randomised trials’. |
| **Additional** | - No restrictions on the date of the publication - Written in English - Within an academic journal, theses, or dissertation | |

**Appendix D – Search strategy for all databases**

| **OVID Medline** | | |
| --- | --- | --- |
|  | **#** | **Search terms** |
| **DVA terms** | **1** | domestic violence.ti,ab |
|  | **2** | (abuse* adj3 wom*n).ti,ab. |
|  | **3** | (abuse* adj3 spous*).ti,ab. |
|  | **4** | (abuse* adj3 partner*).ti,ab. |
|  | **5** | ((wife or wives) adj3 abuse*).ti,ab. |
|  | **6** | ((wife or wives) adj3 batter*).ti,ab. |
|  | **7** | (partner* adj3 violen*).ti,ab. |
|  | **8** | (spous* adj3 violen*).ti,ab. |
|  | **9** | (gender adj3 violen*).ti,ab. |
|  | **10** | (batter* adj3 wom*n).ti,ab. |
|  | **11** | IPV.ti,ab. (6050) |
|  | **12** | (VAW or VAWG or VAWC).ti,ab. (151) |
|  | **13** | interpersonal violence.ti,ab. (1669) |
|  | **14** | domestic violence/ or spouse abuse/ or battered women/ or intimate partner violence/ |
|  | **15** | 1 or 2 or 3 or 4 or 5 or 6 or 7 or 8 or 9 or 10 or 11 or 12 or 13 or 14 |
| **MH terms** | **16** | (mental* adj2 health).ti,ab. |
|  | **17** | (mental* adj3 disorder*).ti,ab. |
|  | **18** | (mental* adj3 ill*).ti,ab. |
|  | **19** | mood disorder*.ti,ab. |
|  | **20** | (well being or well-being or wellbeing).ti,ab. |
|  | **21** | (depression or depressed or depressive disorder).ti,ab. |
|  | **22** | anxiet*.ti,ab. |
|  | **23** | (post-traumatic stress or post traumatic stress or posttraumatic stress or PTSD).ti,ab. |
|  | **24** | (obsessive compulsive disorder or OCD).ti,ab. |
|  | **25** | panic disorder*.ti,ab. |
|  | **26** | trauma.ti,ab. |
|  | **27** | mental health/ or depression/ or exp depressive disorder/ or anxiety/ or exp anxiety disorders/ or exp stress disorders, traumatic/ or exp psychological trauma/ or obsessive-compulsive disorder/ or panic disorder/ |
|  | **28** | 16 or 17 or 18 or 19 or 20 or 21 or 22 or 23 or 24 or 25 or 26 or 27 |
| **SU terms** | **29** | ((substance* or drug* or stimulant* or polydrug*) adj6 (misuse* or "use" or abus* or dependen* or disorder* or addict* or intoxicat*)).ti,ab. |
|  | **30** | ((heroin or opiod* or methadone or temegesic or subutex or opiate* or cocaine or ecstasy or methamphetamine* or crystal meth or amphetamine* or cannabis or marijuana or marihuana or lsd or magic mushrooms or mephedrone or khat or cathinone or ketamine or steroid* or performance enhancing drug* or gammahydroxybutrate or ghb or amyl nitrate) adj3 (misuse* or "use" or abus* or dependen* or disorder* or addict* or intoxicat*)).ti,ab. |
|  | **31** | (alcohol adj3 (dependen* or drink* or intoxicat* or abus* or misus* or risk* or consum* or excess* or reduc* or intervention*)).ti,ab. |
|  | **32** | (drink* adj3 (excess or heavy or heavily or harm or harmful or hazard* or risky or binge or harmful or problem*)).ti,ab. |
|  | **33** | exp alcohol drinking/ or exp "marijuana use"/ or alcohol-related disorders/ or amphetamine-related disorders/ or cocaine-related disorders/ or marijuana abuse/ or "marijuana use"/ or opioid-related disorders/ or substance abuse, intravenous/ or substance abuse, oral/ |
|  | **34** | 29 or 30 or 31 or 32 or 33 |
| **Parent terms** | **35** | (parent or parents or parental).ti,ab. |
|  | **36** | (mother? or mom? or mum? or father? or dad?).ti,ab. |
|  | **37** | (pregnant or pregnancy or postpartum or paternal).ti,ab. |
|  | **38** | (family or families).ti,ab. |
|  | **39** | maternal deprivation/ or parent-child relations/ or father-child relations/ or mother-child relations/ or parenting/ or paternal behavior/ or paternal deprivation/ or nuclear family/ or exp parents/ or single-parent family/ |
|  | **40** | 35 or 36 or 37 or 38 or 39 |
| **RCT terms** | **41** | randomized controlled trial.pt. |
|  | **42** | controlled clinical trial.pt. |
|  | **43** | (randomized or randomised).ab. |
|  | **44** | placebo.ab. |
|  | **45** | clinical trials as topic.sh. |
|  | **46** | randomly.ab. |
|  | **47** | trial.ab. |
|  | **48** | 41 or 42 or 43 or 44 or 45 or 46 or 47 |
|  | **49** | exp animals/ not humans.sh. |
|  | **50** | 48 not 49 |
| **Combo** | **51** | 15 or 28 or 34 |
|  | **52** | 40 and 50 and 51 |
|  | **53** | **limit 52 to english language** |

| **OVID PsycInfo** | | |
| --- | --- | --- |
|  | **#** | **Search terms** |
| **DVA terms** | **1** | domestic violence.ti,ab. |
|  | **2** | (abuse* adj3 wom*n).ti,ab. |
|  | **3** | (abuse* adj3 spous*).ti,ab. |
|  | **4** | (abuse* adj3 partner*).ti,ab. |
|  | **5** | ((wife or wives) adj3 abuse*).ti,ab. |
|  | **6** | ((wife or wives) adj3 batter*).ti,ab. |
|  | **7** | (partner* adj3 violen*).ti,ab. |
|  | **8** | (spous* adj3 violen*).ti,ab. |
|  | **9** | (gender adj3 violen*).ti,ab. |
|  | **10** | (batter* adj3 wom*n).ti,ab. |
|  | **11** | IPV.ti,ab. |
|  | **12** | (VAW or VAWG or VAWC).ti,ab. |
|  | **13** | interpersonal violence.ti,ab. |
|  | **14** | domestic violence/ or battered females/ or intimate partner violence/ or conflict resolution/ |
|  | **15** | 1 or 2 or 3 or 4 or 5 or 6 or 7 or 8 or 9 or 10 or 11 or 12 or 13 or 14 |
| **MH terms** | **16** | (mental* adj2 health).ti,ab. |
|  | **17** | (mental* adj3 disorder*).ti,ab. |
|  | **18** | (mental* adj3 ill*).ti,ab. |
|  | **19** | mood disorder*.ti,ab. |
|  | **20** | (well being or well-being or wellbeing).ti,ab. |
|  | **21** | (depression or depressed or depressive disorder).ti,ab. |
|  | **22** | anxiet*.ti,ab. |
|  | **23** | (post-traumatic stress or post traumatic stress or posttraumatic stress or PTSD).ti,ab. |
|  | **24** | (obsessive compulsive disorder or OCD).ti,ab. |
|  | **25** | panic disorder*.ti,ab. |
|  | **26** | trauma.ti,ab. |
|  | **27** | mental health/ or exp "depression (emotion)"/ or anxiety/ or social anxiety/ or anxiety disorders/ or generalized anxiety disorder/ or obsessive compulsive disorder/ or panic disorder/ or exp posttraumatic stress disorder/ or post-traumatic stress/ |
|  | **28** | 16 or 17 or 18 or 19 or 20 or 21 or 22 or 23 or 24 or 25 or 26 or 27 |
| **SU terms** | **29** | 29 ((substance* or drug* or stimulant* or polydrug*) adj6 (misuse* or "use" or abus* or dependen* or disorder* or addict* or intoxicat*)).ti,ab. |
|  | **30** | ((heroin or opiod* or methadone or temegesic or subutex or opiate* or cocaine or ecstasy or methamphetamine* or crystal meth or amphetamine* or cannabis or marijuana or marihuana or lsd or magic mushrooms or mephedrone or khat or cathinone or ketamine or steroid* or performance enhancing drug* or gammahydroxybutrate or ghb or amyl nitrate) adj3 (misuse* or "use" or abus* or dependen* or disorder* or addict* or intoxicat*)).ti,ab. |
|  | **31** | (alcohol adj3 (dependen* or drink* or intoxicat* or abus* or misus* or risk* or consum* or excess* or reduc* or intervention*)).ti,ab. |
|  | **32** | (drink* adj3 (excess or heavy or heavily or harm or harmful or hazard* or risky or binge or harmful or problem*)).ti,ab. |
|  | **33** | drug usage/ or alcohol drinking patterns/ or heroin addiction/ or intravenous drug usage/ or marijuana usage/ or prescription drug misuse/ or exp alcohol abuse/ or drug abuse prevention/ or addiction/ or "alcohol use disorder"/ or "cannabis use disorder"/ or drug abuse/ or exp drug dependency/ or inhalant abuse/ or "opioid use disorder"/ or "tobacco use disorder"/ |
|  | **34** | 29 or 30 or 31 or 32 or 33 |
| **Parent terms** | **35** | (parent or parents or parental).ti,ab. |
|  | **36** | (mother? or mom? or mum? or father? or dad?).ti,ab. |
|  | **37** | (pregnant or pregnancy or postpartum or paternal).ti,ab. |
|  | **38** | (family or families).ti,ab. |
|  | **39** | exp parents/ or coparenting/ or parenting/ or parent child relations/ or father child relations/ or mother child relations/ or biological family/ or dysfunctional family/ or family history/ or family relations/ or military families/ or nuclear family/ |
|  | **40** | 35 or 36 or 37 or 38 or 39 |
| **RCT terms** | **41** | exp randomized controlled trials/ |
|  | **42** | placebo/ |
|  | **43** | random*.ti,ab. |
|  | **44** | placebo*.ti,ab. |
|  | **45** | (double adj1 blind*).ti,ab. |
|  | **46** | ((compar* or control) adj1 group).ab. |
|  | **47** | 41 or 42 or 43 or 44 or 45 or 46 |
| **Combo** | **48** | 15 or 28 or 34 |
|  | **49** | 40 and 47 and 48 |
|  | **50** | **limit 49 to english language** |

| **OVID Embase** | | |
| --- | --- | --- |
|  | **#** | **Search terms** |
| **DVA terms** | **1** | domestic violence.ti,ab. |
|  | **2** | (abuse* adj3 wom*n).ti,ab. |
|  | **3** | (abuse* adj3 spous*).ti,ab. |
|  | **4** | (abuse* adj3 partner*).ti,ab. |
|  | **5** | ((wife or wives) adj3 abuse*).ti,ab. |
|  | **6** | ((wife or wives) adj3 batter*).ti,ab. |
|  | **7** | (partner* adj3 violen*).ti,ab. |
|  | **8** | (spous* adj3 violen*).ti,ab. |
|  | **9** | (gender adj3 violen*).ti,ab. |
|  | **10** | (batter* adj3 wom*n).ti,ab. |
|  | **11** | IPV.ti,ab. |
|  | **12** | (VAW or VAWG or VAWC).ti,ab. |
|  | **13** | interpersonal violence.ti,ab. |
|  | **14** | domestic violence/ or battered woman/ or exp partner violence/ |
|  | **15** | 1 or 2 or 3 or 4 or 5 or 6 or 7 or 8 or 9 or 10 or 11 or 12 or 13 or 14 |
| **MH terms** | **16** | (mental* adj2 health).ti,ab. |
|  | **17** | (mental* adj3 disorder*).ti,ab. |
|  | **18** | (mental* adj3 ill*).ti,ab. |
|  | **19** | mood disorder*.ti,ab. |
|  | **20** | (well being or well-being or wellbeing).ti,ab. |
|  | **21** | (depression or depressed or depressive disorder).ti,ab. |
|  | **22** | anxiet*.ti,ab. |
|  | **23** | (post-traumatic stress or post traumatic stress or posttraumatic stress or PTSD).ti,ab. |
|  | **24** | (obsessive compulsive disorder or OCD).ti,ab. |
|  | **25** | panic disorder*.ti,ab. |
|  | **26** | trauma.ti,ab. |
|  | **27** | mental health/ or exp depression/ or anxiety/ or anxiety disorders/ or generalized anxiety disorder/ or "mixed anxiety and depression"/ or posttraumatic stress disorder/ or exp psychological trauma/ or obsessive-compulsive disorder/ or panic/ |
|  | **28** | 16 or 17 or 18 or 19 or 20 or 21 or 22 or 23 or 24 or 25 or 26 or 27 |
| **SU terms** | **29** | ((substance* or drug* or stimulant* or polydrug*) adj6 (misuse* or "use" or abus* or dependen* or disorder* or addict* or intoxicat*)).ti,ab. |
|  | **30** | ((heroin or opiod* or methadone or temegesic or subutex or opiate* or cocaine or ecstasy or methamphetamine* or crystal meth or amphetamine* or cannabis or marijuana or marihuana or lsd or magic mushrooms or mephedrone or khat or cathinone or ketamine or steroid* or performance enhancing drug* or gammahydroxybutrate or ghb or amyl nitrate) adj3 (misuse* or "use" or abus* or dependen* or disorder* or addict* or intoxicat*)).ti,ab. |
|  | **31** | (alcohol adj3 (dependen* or drink* or intoxicat* or abus* or misus* or risk* or consum* or excess* or reduc* or intervention*)).ti,ab. |
|  | **32** | (drink* adj3 (excess or heavy or heavily or harm or harmful or hazard* or risky or binge or harmful or problem*)).ti,ab. |
|  | **33** | exp alcohol drinking/ or exp "marijuana use"/ or alcohol-related disorders/ or amphetamine-related disorders/ or cocaine-related disorders/ or marijuana abuse/ or "marijuana use"/ or opioid-related disorders/ or substance abuse/ |
|  | **34** | 29 or 30 or 31 or 32 or 33 |
| **Parent terms** | **35** | (parent or parents or parental).ti,ab. |
|  | **36** | (mother? or mom? or mum? or father? or dad?).ti,ab. |
|  | **37** | (pregnant or pregnancy or postpartum or paternal).ti,ab. |
|  | **38** | (family or families).ti,ab. |
|  | **39** | maternal deprivation/ or parent-child relations/ or father-child relations/ or mother-child relations/ or parenting/ or paternal behavior/ or paternal deprivation/ or nuclear family/ or exp parents/ or single-parent family/ or family/ |
|  | **40** | 35 or 36 or 37 or 38 or 39 |
| **RCT terms** | **41** | placebo/ |
|  | **42** | random*.ti,ab. |
|  | **43** | placebo*.ti,ab. |
|  | **44** | (double adj1 blind*).ti,ab. |
|  | **45** | 41 or 42 or 43 or 44 |
| **Combo** | **46** | 15 or 28 or 34 |
|  | **47** | 40 and 45 and 46 |
|  | **48** | **limit 47 to english language** |

| **EBSCOhost CINAHL** | | |
| --- | --- | --- |
|  | **#** | **Search terms** |
| **DVA terms** | **S1** | TI "domestic violence" OR AB "domestic violence" |
|  | **S2** | TI abuse* N2 wom?n OR AB abuse* N2 wom?n |
|  | **S3** | TI abuse* N2 spous* OR AB abuse* N2 spous* |
|  | **S4** | TI abuse* N2 partner* OR AB abuse* N2 partner* |
|  | **S5** | TI ((wife or wives) N2 abuse*) OR AB ((wife or wives) N2 abuse*) |
|  | **S6** | TI ((wife or wives) N2 batter*) OR AB ((wife or wives) N2 batter*) |
|  | **S7** | TI partner* N2 violen* OR AB partner* N2 violen* |
|  | **S8** | TI spous* N2 violen* OR AB spous* N2 violen* |
|  | **S9** | TI gender N2 violen* OR AB gender N2 violen* |
|  | **S10** | TI batter* N2 wom?n OR AB batter* N2 wom?n |
|  | **S11** | TI IPV OR AB IPV |
|  | **S12** | TI (VAW or VAWG or VAWC) OR AB (VAW or VAWG or VAWC) |
|  | **S13** | TI "interpersonal violence" OR AB "interpersonal violence" |
|  | **S14** | (MH "Domestic Violence") OR (MH "Intimate Partner Violence") OR (MH "Gender-Based Violence") |
|  | **S15** | S1 OR S2 OR S3 OR S4 OR S5 OR S6 OR S7 OR S8 OR S9 OR S10 OR S11 OR S12 OR S13 OR S14 |
| **MH terms** | **S16** | TI mental* N1 health OR AB mental* N1 health |
|  | **S17** | TI mental* N2 disorder* OR AB mental* N2 disorder* |
|  | **S18** | TI mental* N2 ill* OR AB mental* N2 ill* |
|  | **S19** | TI "mood disorder*" OR AB "mood disorder*" |
|  | **S20** | TI ("well#being" or "well being") OR AB ("well#being" or "well being") |
|  | **S21** | TI (depression or depressed or "depressive disorder") OR AB (depression or depressed or "depressive disorder") |
|  | **S22** | TI anxiet* OR AB anxiet* |
|  | **S23** | TI ("post#traumatic stress" or "post traumatic stress" or "PTSD") OR AB ("post#traumatic stress" or "post traumatic stress" or "PTSD") |
|  | **S24** | TI ("obsessive compulsive disorder" or OCD) OR AB ("obsessive compulsive disorder" or OCD) |
|  | **S25** | TI "panic disorder*" OR AB "panic disorder*" |
|  | **S26** | TI "trauma" OR AB "trauma" |
|  | **S27** | (MH "Mental Health") OR (MH "Depression") OR (MH "Anxiety") OR (MH "Stress Disorders, Post-Traumatic") OR (MH "Obsessive-Compulsive Disorder") OR (MH "Panic Disorder") OR (MH "Generalized Anxiety Disorder") OR (MH "Social Anxiety Disorders") |
|  | **S28** | S16 OR S17 OR S18 OR S19 OR S20 OR S21 OR S22 OR S23 OR S24 OR S25 OR S26 OR S27 |
| **SU terms** | **S29** | TI ( (substance* or drug* or stimulant* or polydrug*) N5 (misuse* or use* or abus* or dependen* or disorder* or addict* or intoxicat*) ) OR AB ( (substance* or drug* or stimulant* or polydrug*) N5 (misuse* or "use" or abus* or dependen* or disorder* or addict* or intoxicat*) ) |
|  | **S30** | TI ((heroin or opiod* or methadone or temegesic or subutex or opiate* or cocaine or ecstasy or methamphetamine* or "crystal meth" or amphetamine* or cannabis or marijuana or marihuana or lsd or "magic mushrooms" or mephedrone or khat or cathinone or ketamine or steroid* or "performance enhancing drug*" or gammahydroxybutrate or ghb or "amyl nitrate" ) N2 (misuse* or "use" or abus* or dependen* or disorder* or addict* or intoxicat*)) OR AB ((heroin or opiod* or methadone or temegesic or subutex or opiate* or cocaine or ecstasy or methamphetamine* or "crystal meth" or amphetamine* or cannabis or marijuana or marihuana or lsd or "magic mushrooms" or mephedrone or khat or cathinone or ketamine or steroid* or "performance enhancing drug*" or gammahydroxybutrate or ghb or "amyl nitrate" ) N2 (misuse* or "use" or abus* or dependen* or disorder* or addict* or intoxicat*)) |
|  | **S31** | TI (alcohol N2 (dependen* or drink* or intoxicat* or abus* or misus* or risk* or consum* or excess* or reduc* or intervention*)) OR AB (alcohol N2 (dependen* or drink* or intoxicat* or abus* or misus* or risk* or consum* or excess* or reduc* or intervention*)) |
|  | **S32** | TI (drink* N2 (excess or heavy or heavily or harm or harmful or hazard* or risky or binge or harmful or problem*)) OR AB (drink* N2 (excess or heavy or heavily or harm or harmful or hazard* or risky or binge or harmful or problem*)) |
|  | **S33** | (MH "Substance Abuse+") |
|  | **S34** | S29 OR S30 OR S31 OR S32 OR S33 |
| **Parent terms** | **S35** | TI (parent or parents or parental) OR AB (parent or parents or parental) |
|  | **S36** | TI (mother# or mom# or mum# or father# or dad#) OR AB (mother# or mom# or mum# or father# or dad#) |
|  | **S37** | TI (pregnant or pregnancy or postpartum or paternal) OR AB (pregnant or pregnancy or postpartum or paternal) |
|  | **S38** | TI (family or families) OR AB (family or families) |
|  | **S39** | (MH "Parents+") OR (MH "Parent-Child Relations+") OR (MH "Parenting") |
|  | **S40** | S35 OR S36 OR S37 OR S38 OR S39 |
| **RCT terms** | **S41** | MH randomized controlled trials |
|  | **S42** | MH double-blind studies |
|  | **S43** | MH single-blind studies |
|  | **S44** | MH random assignment |
|  | **S45** | MH pretest-posttest design |
|  | **S46** | MH cluster sample |
|  | **S47** | TI (randomised OR randomized) |
|  | **S48** | AB (random*) |
|  | **S49** | TI (trial) |
|  | **S50** | MH (sample size) AND AB (assigned OR allocated OR control) |
|  | **S51** | MH (placebos) |
|  | **S52** | PT (randomized controlled trial) |
|  | **S53** | AB (control W5 group) |
|  | **S54** | MH (crossover design) OR MH (comparative studies) |
|  | **S55** | AB (cluster W3 RCT) |
|  | **S56** | MH animals+ |
|  | **S57** | MH (animal studies) |
|  | **S58** | TI (animal model*) |
|  | **S59** | S56 OR S57 OR S58 |
|  | **S60** | MH (human) |
|  | **S61** | S59 NOT S60 |
|  | **S62** | S41 OR S42 OR S43 OR S44 OR S45 OR S46 OR S47 OR S48 OR S49 OR S50 OR S51 OR S52 OR S53 OR S54 OR S55 |
|  | **S63** | S62 NOT S61 |
| **Combo** | **S64** | S15 OR S28 OR S34 |
|  | **S65** | S40 AND S63 AND S64 |
|  | **S66** | **S65 AND LA English** |

| **EBSCOhost ERIC** | | |
| --- | --- | --- |
|  | **#** | **Search terms** |
| **DVA terms** | **S1** | TI "domestic violence" OR AB "domestic violence" |
|  | **S2** | TI abuse* N2 wom?n OR AB abuse* N2 wom?n |
|  | **S3** | TI abuse* N2 spous* OR AB abuse* N2 spous* |
|  | **S4** | TI abuse* N2 partner* OR AB abuse* N2 partner* |
|  | **S5** | TI ((wife or wives) N2 abuse*) OR AB ((wife or wives) N2 abuse*) |
|  | **S6** | TI ((wife or wives) N2 batter*) OR AB ((wife or wives) N2 batter*) |
|  | **S7** | TI partner* N2 violen* OR AB partner* N2 violen* |
|  | **S8** | TI spous* N2 violen* OR AB spous* N2 violen* |
|  | **S9** | TI gender N2 violen* OR AB gender N2 violen* |
|  | **S10** | TI batter* N2 wom?n OR AB batter* N2 wom?n |
|  | **S11** | TI IPV OR AB IPV |
|  | **S12** | TI (VAW or VAWG or VAWC) OR AB (VAW or VAWG or VAWC) |
|  | **S13** | TI "interpersonal violence" OR AB "interpersonal violence" |
|  | **S14** | DE "Family Violence" |
|  | **S15** | S1 OR S2 OR S3 OR S4 OR S5 OR S6 OR S7 OR S8 OR S9 OR S10 OR S11 OR S12 OR S13 OR S14 |
| **MH terms** | **S16** | TI mental* N1 health OR AB mental* N1 health |
|  | **S17** | TI mental* N2 disorder* OR AB mental* N2 disorder* |
|  | **S18** | TI mental* N2 ill* OR AB mental* N2 ill* |
|  | **S19** | TI "mood disorder*" OR AB "mood disorder*" |
|  | **S20** | TI ("well#being" or "well being") OR AB ("well#being" or "well being") |
|  | **S21** | TI (depression or depressed or "depressive disorder") OR AB (depression or depressed or "depressive disorder") |
|  | **S22** | TI anxiet* OR AB anxiet* |
|  | **S23** | TI ("post#traumatic stress" or "post traumatic stress" or "PTSD") OR AB ("post#traumatic stress" or "post traumatic stress" or "PTSD") |
|  | **S24** | TI ("obsessive compulsive disorder" or OCD) OR AB ("obsessive compulsive disorder" or OCD) |
|  | **S25** | TI "panic disorder*" OR AB "panic disorder*" |
|  | **S26** | TI "trauma" OR AB "trauma" |
|  | **S27** | DE "Anxiety" OR DE "Depression (Psychology)" OR DE "Posttraumatic Stress Disorder" OR DE "Anxiety Disorders" OR DE "Mental Health" |
|  | **S28** | S16 OR S17 OR S18 OR S19 OR S20 OR S21 OR S22 OR S23 OR S24 OR S25 OR S26 OR S27 |
| **SU terms** | **S29** | TI ( (substance* or drug* or stimulant* or polydrug*) N5 (misuse* or use* or abus* or dependen* or disorder* or addict* or intoxicat*) ) OR AB ( (substance* or drug* or stimulant* or polydrug*) N5 (misuse* or "use" or abus* or dependen* or disorder* or addict* or intoxicat*) ) |
|  | **S30** | TI ((heroin or opiod* or methadone or temegesic or subutex or opiate* or cocaine or ecstasy or methamphetamine* or "crystal meth" or amphetamine* or cannabis or marijuana or marihuana or lsd or "magic mushrooms" or mephedrone or khat or cathinone or ketamine or steroid* or "performance enhancing drug*" or gammahydroxybutrate or ghb or "amyl nitrate" ) N2 (misuse* or "use" or abus* or dependen* or disorder* or addict* or intoxicat*)) OR AB ((heroin or opiod* or methadone or temegesic or subutex or opiate* or cocaine or ecstasy or methamphetamine* or "crystal meth" or amphetamine* or cannabis or marijuana or marihuana or lsd or "magic mushrooms" or mephedrone or khat or cathinone or ketamine or steroid* or "performance enhancing drug*" or gammahydroxybutrate or ghb or "amyl nitrate" ) N2 (misuse* or "use" or abus* or dependen* or disorder* or addict* or intoxicat*)) |
|  | **S31** | TI (alcohol N2 (dependen* or drink* or intoxicat* or abus* or misus* or risk* or consum* or excess* or reduc* or intervention*)) OR AB (alcohol N2 (dependen* or drink* or intoxicat* or abus* or misus* or risk* or consum* or excess* or reduc* or intervention*)) |
|  | **S32** | TI (drink* N2 (excess or heavy or heavily or harm or harmful or hazard* or risky or binge or harmful or problem*)) OR AB (drink* N2 (excess or heavy or heavily or harm or harmful or hazard* or risky or binge or harmful or problem*)) |
|  | **S33** | DE "Alcohol Abuse" OR DE "Alcoholism" OR DE "Drug Abuse" OR DE "Drug Addiction" |
|  | **S34** | S29 OR S30 OR S31 OR S32 OR S33 |
| **Parent terms** | **S35** | TI (parent or parents or parental) OR AB (parent or parents or parental) |
|  | **S36** | TI (mother# or mom# or mum# or father# or dad#) OR AB (mother# or mom# or mum# or father# or dad#) |
|  | **S37** | TI (pregnant or pregnancy or postpartum or paternal) OR AB (pregnant or pregnancy or postpartum or paternal) |
|  | **S38** | TI (family or families) OR AB (family or families) |
|  | **S39** | DE "Family (Sociological Unit)" OR DE "Parent Child Relationship" OR DE "Early Parenthood" OR DE "Fathers" OR DE "Mothers" OR DE "Unwed Mothers" OR DE "Parenting Skills" |
|  | **S40** | S35 OR S36 OR S37 OR S38 OR S39 |
| **RCT terms** | **S41** | DE "randomized controlled trials" |
|  | **S42** | DE "Control Groups" OR DE "Experimental Groups" |
|  | **S43** | TI (randomized or randomised or RCT) OR AB (randomized or randomised or RCT) |
|  | **S44** | AB placebo |
|  | **S45** | AB ((control or compar*) N5 (group) |
|  | **S46** | AB randomly |
|  | **S47** | AB trial# |
|  | **S48** | AB groups |
|  | **S49** | AB intervention* |
|  | **S50** | AB experiment* |
|  | **S51** | S41 OR S42 OR S43 OR S44 OR S45 OR S46 OR S47 OR S48 OR S49 OR S50 |
| **Combo** | **S52** | S15 OR S28 OR S34 |
|  | **S53** | S40 AND S51 AND S52 |
|  | **S54** | **S53 AND LA English** |

| **ProQuest Sociological Abstracts** | | |
| --- | --- | --- |
|  | **#** | **Search terms** |
| **DVA terms** | **S1** | TI,AB("domestic violence") |
|  | **S2** | TI,AB(abuse* NEAR/3 wom?n) |
|  | **S3** | TI,AB(abuse* NEAR/3 spous*) |
|  | **S4** | TI,AB(abuse* NEAR/3 partner*) |
|  | **S5** | TI,AB((wife OR wives) NEAR/3 abuse*) |
|  | **S6** | TI,AB((wife OR wives) NEAR/3 batter*) |
|  | **S7** | TI,AB(partner* NEAR/3 violen*) |
|  | **S8** | TI,AB(spous* NEAR/3 violen*) |
|  | **S9** | TI,AB(gender NEAR/3 violen*) |
|  | **S10** | TI,AB(batter* NEAR/3 wom?n) |
|  | **S11** | TI,AB(IPV) |
|  | **S12** | TI,AB(VAW OR VAWG OR VAWC) |
|  | **S13** | TI,AB("interpersonal violence") |
|  | **S14** | mainsubject.Exact("intimate partner violence" OR "battered women" OR "domestic violence" OR "spouse abuse" OR "partner abuse") |
|  | **S15** | S1 OR S2 OR S3 OR S4 OR S5 OR S6 OR S7 OR S8 OR S9 OR S10 OR S11 OR S12 OR S13 OR S14 |
| **MH terms** | **S16** | TI,AB(mental* NEAR/2 health) |
|  | **S17** | TI,AB(mental* NEAR/3 disorder*) |
|  | **S18** | TI,AB(mental* NEAR/3 ill*) |
|  | **S19** | TI,AB("mood disorder" OR "mood disorders") |
|  | **S20** | TI,AB("well being" OR well-being OR wellbeing) |
|  | **S21** | TI,AB(depression OR depressed OR "depressive disorder") |
|  | **S22** | TI,AB(anxiet*) |
|  | **S23** | TI,AB("post-traumatic stress" OR "post traumatic stress" OR "posttraumatic stress" OR PTSD) |
|  | **S24** | TI,AB("obsessive compulsive disorder" OR OCD) |
|  | **S25** | TI,AB("panic disorder" OR "panic disorders") |
|  | **S26** | TI,AB(trauma) |
|  | **S27** | mainsubject.exact("Mental Illness" OR "Posttraumatic Stress Disorder" OR "Anxiety" OR "Trauma" OR "Mental Health" OR "Obsessive Compulsive Disorder" OR "Panic Disorder" OR "Depression (Psychology)") |
|  | **S28** | S16 OR S17 OR S18 OR S19 OR S20 OR S21 OR S22 OR S23 OR S24 OR S25 OR S26 OR S27 |
| **SU terms** | **S29** | TI,AB((substance* OR drug* OR stimulant* OR polydrug* OR alcohol) NEAR/6 (misuse* OR "use" OR abus* OR dependen* OR disorder* OR addict* OR intoxicat*)) |
|  | **S30** | MAINSUBJECT.EXACT("drug dependency" OR "drug abuse" OR "alcohol abuse" OR "alcohol dependence" OR "alcoholic parents" OR "drug addiction" OR "alcohol drinking" OR "alcohol use" OR "drinking behaviours") |
|  | **S31** | S29 OR S30 |
| **Parent terms** | **S32** | TI,AB(parent OR parents OR parental) |
|  | **S33** | TI,AB(mother? OR mom? OR mum? OR father? OR dad?) |
|  | **S34** | TI,AB(pregnant OR pregnancy OR postpartum OR paternal) |
|  | **S35** | TI,AB(family OR families) |
|  | **S36** | MAINSUBJECT.EXACT("Parent Child Relations" OR "Nuclear Family" OR "Parents" OR "Single Parent Family") |
|  | **S37** | S32 OR S33 OR S34 OR S35 OR S36 |
| **RCT terms** | **S38** | Exact("randomised controlled trials" OR "clinical randomized controlled trials" OR "randomized controlled trials as topic" OR "randomized controlled trials" OR "cluster randomized controlled trials" OR "trials" OR "control groups") |
|  | **S39** | TI,AB(randomized OR randomised OR RCT) |
|  | **S40** | AB(placebo) |
|  | **S41** | AB(randomly) |
|  | **S42** | AB(trial?) |
|  | **S43** | AB(groups) |
|  | **S44** | AB((control OR compar*) NEAR/4 (group)) |
|  | **S45** | AB(intervention*) |
|  | **S46** | AB(experiment*) |
|  | **S47** | S38 OR S39 OR S40 OR S41 OR S42 OR S43 OR S44 OR S45 OR S46 |
| **Combo** | **S48** | S15 OR S28 OR S31 |
|  | **S49** | S48 AND S37 AND S47 |
|  | **S50** | **(S48 AND S37 AND S47) AND la.exact("ENG")** |

| **ProQuest Applied Social Sciences Index & Abstracts (ASSIA)** | | |
| --- | --- | --- |
|  | **#** | **Search terms** |
| **DVA terms** | **S1** | TI,AB("domestic violence") |
|  | **S2** | TI,AB(abuse* NEAR/3 wom?n) |
|  | **S3** | TI,AB(abuse* NEAR/3 spous*) |
|  | **S4** | TI,AB(abuse* NEAR/3 partner*) |
|  | **S5** | TI,AB((wife OR wives) NEAR/3 abuse*) |
|  | **S6** | TI,AB((wife OR wives) NEAR/3 batter*) |
|  | **S7** | TI,AB(partner* NEAR/3 violen*) |
|  | **S8** | TI,AB(spous* NEAR/3 violen*) |
|  | **S9** | TI,AB(gender NEAR/3 violen*) |
|  | **S10** | TI,AB(batter* NEAR/3 wom?n) |
|  | **S11** | TI,AB(IPV) |
|  | **S12** | TI,AB(VAW OR VAWG OR VAWC) |
|  | **S13** | TI,AB("interpersonal violence") |
|  | **S14** | mainsubject.Exact("Domestic violence" OR "Battered women") |
|  | **S15** | S1 OR S2 OR S3 OR S4 OR S5 OR S6 OR S7 OR S8 OR S9 OR S10 OR S11 OR S12 OR S13 OR S14 |
| **MH terms** | **S16** | TI,AB(mental* NEAR/2 health) |
|  | **S17** | TI,AB(mental* NEAR/3 disorder*) |
|  | **S18** | TI,AB(mental* NEAR/3 ill*) |
|  | **S19** | TI,AB("mood disorder" OR "mood disorders") |
|  | **S20** | TI,AB("well being" OR well-being OR wellbeing) |
|  | **S21** | TI,AB(depression OR depressed OR "depressive disorder") |
|  | **S22** | TI,AB(anxiet*) |
|  | **S23** | TI,AB("post-traumatic stress" OR "post traumatic stress" OR "posttraumatic stress" OR PTSD) |
|  | **S24** | TI,AB("obsessive compulsive disorder" OR OCD) |
|  | **S25** | TI,AB("panic disorder" OR "panic disorders") |
|  | **S26** | TI,AB(trauma) |
|  | **S27** | mainsubject.Exact("Anxiety disorders" OR "Obsessive-Compulsive neuroses" OR Generalized anxiety disorders" OR Posttraumatic stress disorder" OR "Parental depression" OR "Mental health" OR "Depression" OR "Panic disorders") OR MAINSUBJECT.EXACT.EXPLODE("Anxiety") |
|  | **S28** | S16 OR S17 OR S18 OR S19 OR S20 OR S21 OR S22 OR S23 OR S24 OR S25 OR S26 OR S27 |
| **SU terms** | **S29** | TI,AB((substance* OR drug* OR stimulant* OR polydrug* OR alcohol) NEAR/6 (misuse* OR "use" OR abus* OR dependen* OR disorder* OR addict* OR intoxicat*)) |
|  | **S30** | mainsubject.Exact("Substance dependency" OR "Drug dependency" OR "Substance abuse disorders" OR "Alcohol dependence" or "alcohol use") OR MAINSUBJECT.EXACT.EXPLODE("Addiction" OR "Substance abuse" OR "Abusers") |
|  | **S31** | S29 OR S30 |
| **Parent terms** | **S32** | TI,AB(parent OR parents OR parental) |
|  | **S33** | TI,AB(mother? OR mom? OR mum? OR father? OR dad?) |
|  | **S34** | TI,AB(pregnant OR pregnancy OR postpartum OR paternal) |
|  | **S35** | TI,AB(family OR families) |
|  | **S36** | MAINSUBJECT.EXACT("Parent-Child relationships" OR "Parents" OR "Adolescent parents" OR "Mothers" OR "Fathers" OR "Single parents" OR "Single parent families" OR "Mentally ill parents" OR "Alcoholic parents") |
|  | **S37** | S32 OR S33 OR S34 OR S35 OR S36 |
| **RCT terms** | **S38** | MAINSUBJECT.EXACT("Single blind randomized controlled trials" OR "Clinical trials" OR "Randomized controlled trials" OR "Double blind randomized controlled trials" OR "Cluster randomized controlled trials" OR "Clinical randomized controlled trials") |
|  | **S39** | TI,AB(randomized OR randomised OR RCT) |
|  | **S40** | AB(placebo) |
|  | **S41** | AB(randomly) |
|  | **S42** | AB(trial?) |
|  | **S43** | AB(groups) |
|  | **S44** | AB((control OR compar*) NEAR/4 (group)) |
|  | **S45** | AB(intervention*) |
|  | **S46** | AB(experiment*) |
|  | **S47** | S38 OR S39 OR S40 OR S41 OR S42 OR S43 OR S44 OR S45 OR S46 |
| **Combo** | **S48** | S15 OR S28 OR S31 |
|  | **S49** | S48 AND S37 AND S47 |
|  | **S50** | **(S48 AND S37 AND S47) AND la.exact("ENG")** |

| **ProQuest ProQuest Dissertations & Theses Global‎** | | |
| --- | --- | --- |
|  | **#** | **Search terms** |
| **DVA terms** | **S1** | TI,AB("domestic violence") |
|  | **S2** | TI,AB(abuse* NEAR/3 wom?n) |
|  | **S3** | TI,AB(abuse* NEAR/3 spous*) |
|  | **S4** | TI,AB(abuse* NEAR/3 partner*) |
|  | **S5** | TI,AB((wife OR wives) NEAR/3 abuse*) |
|  | **S6** | TI,AB((wife OR wives) NEAR/3 batter*) |
|  | **S7** | TI,AB(partner* NEAR/3 violen*) |
|  | **S8** | TI,AB(spous* NEAR/3 violen*) |
|  | **S9** | TI,AB(gender NEAR/3 violen*) |
|  | **S10** | TI,AB(batter* NEAR/3 wom?n) |
|  | **S11** | TI,AB(IPV) |
|  | **S12** | TI,AB(VAW OR VAWG OR VAWC) |
|  | **S13** | TI,AB("interpersonal violence") |
|  | **S14** | Exact("domestic violence") |
|  | **S15** | S1 OR S2 OR S3 OR S4 OR S5 OR S6 OR S7 OR S8 OR S9 OR S10 OR S11 OR S12 OR S13 OR S14 |
| **MH terms** | **S16** | TI,AB(mental* NEAR/2 health) |
|  | **S17** | TI,AB(mental* NEAR/3 disorder*) |
|  | **S18** | TI,AB(mental* NEAR/3 ill*) |
|  | **S19** | TI,AB("mood disorder" OR "mood disorders") |
|  | **S20** | TI,AB("well being" OR well-being OR wellbeing) |
|  | **S21** | TI,AB(depression OR depressed OR "depressive disorder") |
|  | **S22** | TI,AB(anxiet*) |
|  | **S23** | TI,AB("post-traumatic stress" OR "post traumatic stress" OR "posttraumatic stress" OR PTSD) |
|  | **S24** | TI,AB("obsessive compulsive disorder" OR OCD) |
|  | **S25** | TI,AB("panic disorder" OR "panic disorders") |
|  | **S26** | TI,AB(trauma) |
|  | **S27** | Exact("anxiety" OR "mental health" OR "post traumatic stress disorder" OR "obsessive compulsive disorder" OR "mental depression") |
|  | **S28** | S16 OR S17 OR S18 OR S19 OR S20 OR S21 OR S22 OR S23 OR S24 OR S25 OR S26 OR S27 |
| **SU terms** | **S29** | TI,AB((substance* OR drug* OR stimulant* OR polydrug* OR alcohol) NEAR/6 (misuse* OR "use" OR abus* OR dependen* OR disorder* OR addict* OR intoxicat*)) |
|  | **S30** | Exact("drug abuse" OR "alcohol use" OR "alcoholism" OR "drug addiction" OR "drug use") |
|  | **S31** | S29 OR S30 |
| **Parent terms** | **S32** | TI,AB(parent OR parents OR parental) |
|  | **S33** | TI,AB(mother? OR mom? OR mum? OR father? OR dad?) |
|  | **S34** | TI,AB(pregnant OR pregnancy OR postpartum OR paternal) |
|  | **S35** | TI,AB(family OR families) |
|  | **S36** | Exact("families & family life" OR "parents & parenting" OR "teenage parents" OR "single parents") |
|  | **S37** | S32 OR S33 OR S34 OR S35 OR S36 |
| **RCT terms** | **S38** | TI,AB(random*) |
|  | **S39** | TI,AB(experiment*) |
|  | **S40** | S38 OR S39 |
| **Combo** | **S41** | S15 OR S28 OR S31 |
|  | **S42** | S41 AND S37 AND S40 |
|  | **S43** | **(S41 AND S37 AND S40) AND la.exact("ENG")** |

| **Web of Science Web of Science Core Collection** | | |
| --- | --- | --- |
|  | **#** | **Search terms** |
| **DVA terms** | **#1** | TS="domestic violence" |
|  | **#2** | TI=(abuse* NEAR/3 wom?n) |
|  | **#3** | TI=(abuse* NEAR/3 spous*) |
|  | **#4** | TI=(abuse* NEAR/3 partner*) |
|  | **#5** | TI=((wife or wives) NEAR/3 abuse*) |
|  | **#6** | TI=((wife or wives) NEAR/3 batter*) |
|  | **#7** | TI=(partner* NEAR/3 violen*) |
|  | **#8** | TI=(spous* NEAR/3 violen*) |
|  | **#9** | TI=(gender NEAR/3 violen*) |
|  | **#10** | TI=(batter* NEAR/3 wom?n) |
|  | **#11** | TS=IPV |
|  | **#12** | TS=(VAW or VAWG or VAWC) |
|  | **#13** | TS="interpersonal violence" |
|  | **#14** | #13 OR #12 OR #11 OR #10 OR #9 OR #8 OR #7 OR #6 OR #5 OR #4 OR #3 OR #2 OR #1 |
| **MH terms** | **#15** | TS=(mental* NEAR/2 health) |
|  | **#16** | TI=(mental* NEAR/3 disorder*) |
|  | **#17** | TI=(mental* NEAR/3 ill*) |
|  | **#18** | TI="mood disorder*" |
|  | **#19** | TI=(well being or well$being) |
|  | **#20** | TI=(depression or depressed or depressive disorder) |
|  | **#21** | TI=anxiet* |
|  | **#22** | TI=("post traumatic stress" or "post$traumatic stress" or PTSD) |
|  | **#23** | TI=("obsessive compulsive disorder" or OCD) |
|  | **#24** | TI="panic disorder*" |
|  | **#25** | TI=trauma |
|  | **#26** | #25 OR #24 OR #23 OR #22 OR #21 OR #20 OR #19 OR #18 OR #17 OR #16 OR #15 |
| **SU terms** | **#27** | TS=((substance* or drug* or stimulant* or polydrug*) NEAR/6 (misuse* or "use" or abus* or dependen* or disorder* or addict* or intoxicat*)) |
|  | **#28** | TI=((heroin or opiod* or methadone or temegesic or subutex or opiate* or cocaine or ecstasy or methamphetamine* or "crystal meth" or amphetamine* or cannabis or marijuana or marihuana or lsd or "magic mushrooms" or mephedrone or khat or cathinone or ketamine or steroid* or "performance enhancing drug*" or gammahydroxybutrate or ghb or "amyl nitrate") NEAR/3 (misuse* or "use" or abus* or dependen* or disorder* or addict* or intoxicat*)) |
|  | **#29** | TI=(alcohol NEAR/3 (dependen* or drink* or intoxicat* or abus* or misus* or risk* or consum* or excess* or reduc* or intervention*)) |
|  | **#30** | TI=(drink* NEAR/3 (excess or heavy or heavily or harm or harmful or hazard* or risky or binge or harmful or problem*)) |
|  | **#31** | #30 OR #29 OR #28 OR #27 |
| **Parent terms** | **#32** | TS=(parent or parents or parental) |
|  | **#33** | TI=(mother$ or mom$ or mum$ or father$ or dad$) |
|  | **#34** | TI=(pregnant or pregnancy or postpartum or paternal) |
|  | **#35** | TI=(family or families) |
|  | **#36** | #35 OR #34 OR #33 OR #32 |
| **RCT terms** | **#37** | TS=(randomized or randomised or RCT) |
|  | **#38** | TI=(placebo) |
|  | **#39** | TS=(randomly) |
|  | **#40** | TI=(trial$) |
|  | **#41** | TI=(groups) |
|  | **#42** | TI=((control or compar*) NEAR/4 group) |
|  | **#43** | TS=(intervention*) |
|  | **#44** | TI=(experiment*) |
|  | **#45** | #44 OR #43 OR #42 OR #41 OR #40 OR #39 OR #38 OR #37 |
| **Combo** | **#46** | #31 OR #26 OR #14 |
|  | **#47** | #46 AND #45 AND #36 |
|  | **#48** | **(#47) *AND*LANGUAGE: (English)** |

| **Cochrane CENTRAL** | | |
| --- | --- | --- |
|  | **#** | **Search terms** |
| **DVA terms** | **#1** | ("domestic violence"):ti OR ("domestic violence"):ab in Trials |
|  | **#2** | (abuse* NEAR/3 wom?n):ti OR (abuse* NEAR/3 wom?n):ab in Trials |
|  | **#3** | (abuse* NEAR/3 spous*):ti OR (abuse* NEAR/3 spous*):ab in Trials |
|  | **#4** | (abuse* NEAR/3 partner*):ti OR (abuse* NEAR/3 partner*):ab in Trials |
|  | **#5** | ((wife or wives) NEAR/3 abuse*):ti OR ((wife or wives) NEAR/3 abuse*):ab in Trials |
|  | **#6** | ((wife or wives) NEAR/3 batter*):ti OR ((wife or wives) NEAR/3 batter*):ab in Trials |
|  | **#7** | (partner* NEAR/3 violen*):ti OR (partner* NEAR/3 violen*):ab in Trials |
|  | **#8** | (spous* NEAR/3 violen*):ti OR (spous* NEAR/3 violen*):ab in Trials |
|  | **#9** | (gender NEAR/3 violen*):ti OR (gender NEAR/3 violen*):ab in Trials |
|  | **#10** | (batter* NEAR/3 wom?n):ti OR (batter* NEAR/3 wom?n):ab in Trials |
|  | **#11** | (IPV):ti OR (IPV):ab in Trials |
|  | **#12** | (VAW or VAWG or VAWC):ti OR (VAW or VAWG or VAWC):ab in Trials |
|  | **#13** | ("interpersonal violence"):ti OR ("interpersonal violence"):ab in Trials |
|  | **#14** | MeSH descriptor: [Domestic Violence] this term only |
|  | **#15** | MeSH descriptor: [Intimate Partner Violence] explode all trees |
|  | **#16** | MeSH descriptor: [Battered Women] this term only |
|  | **#17** | #1 OR #2 OR #3 OR #4 OR #5 OR #6 OR #7 OR #8 OR #9 OR #10 OR #11 OR #12 OR #13 OR #14 OR #15 OR #16 in Trials |
| **MH terms** | **#18** | (mental* NEAR/2 health):ti OR (mental* NEAR/2 health):ab in Trials |
|  | **#19** | (mental* NEAR/3 disorder*):ti OR (mental* NEAR/3 disorder*):ab in Trials |
|  | **#20** | (mental* NEAR/3 ill*):ti OR (mental* NEAR/3 ill*):ab in Trials |
|  | **#21** | (mood NEXT disorder*):ti OR (mood NEXT disorder*):ab in Trials |
|  | **#22** | ("well being" or well?being):ti OR ("well being" or well?being):ab in Trials |
|  | **#23** | (depression or depressed or "depressive disorder"):ti OR (depression or depressed or "depressive disorder"):ab in Trials |
|  | **#24** | (anxiet*):ti OR (anxiet*):ab in Trials |
|  | **#25** | ("post traumatic stress" or (post?traumatic NEXT stress) or PTSD):ti OR ("post traumatic stress" or (post?traumatic NEXT stress) or PTSD):ab in Trials 5 |
|  | **#26** | ("obsessive compulsive disorder" or OCD):ti OR ("obsessive compulsive disorder" or OCD):ab in Trials |
|  | **#27** | (panic NEXT disorder*):ti OR (panic NEXT disorder*):ab in Trials |
|  | **#28** | (trauma):ti OR (trauma):ab in Trials |
|  | **#29** | MeSH descriptor: [Mental Health] this term only |
|  | **#30** | MeSH descriptor: [Depression] this term only |
|  | **#31** | MeSH descriptor: [Anxiety] this term only |
|  | **#32** | MeSH descriptor: [Stress Disorders, Post-Traumatic] this term only |
|  | **#33** | MeSH descriptor: [Obsessive-Compulsive Disorder] this term only |
|  | **#34** | MeSH descriptor: [Panic Disorder] this term only |
|  | **#35** | #18 OR #19 OR #20 OR #21 OR #22 OR #23 OR #24 OR #25 OR #26 OR #27 OR #28 OR #29 OR #30 OR #31 OR #32 OR #33 OR #34 in Trials |
| **SU terms** | **#36** | ((substance* or drug* or stimulant* or polydrug*) NEAR/6 (misuse* or "use" or abus* or dependen* or disorder* or addict* or intoxicat*)):ti OR ((substance* or drug* or stimulant* or polydrug*) NEAR/6 (misuse* or "use" or abus* or dependen* or disorder* or addict* or intoxicat*)):ab in Trials |
|  | **#37** | ((heroin or opiod* or methadone or temegesic or subutex or opiate* or cocaine or ecstasy or methamphetamine* or "crystal meth" or amphetamine* or cannabis or marijuana or marihuana or lsd or "magic mushrooms" or mephedrone or khat or cathinone or ketamine or steroid* or (performance NEXT enhancing NEXT drug*) or gammahydroxybutrate or ghb or "amyl nitrate") NEAR/3 (misuse* or "use" or abus* or dependen* or disorder* or addict* or intoxicat*)):ti OR ((heroin or opiod* or methadone or temegesic or subutex or opiate* or cocaine or ecstasy or methamphetamine* or "crystal meth" or amphetamine* or cannabis or marijuana or marihuana or lsd or "magic mushrooms" or mephedrone or khat or cathinone or ketamine or steroid* or (performance NEXT enhancing NEXT drug*) or gammahydroxybutrate or ghb or "amyl nitrate") NEAR/3 (misuse* or "use" or abus* or dependen* or disorder* or addict* or intoxicat*)):ab in Trials |
|  | **#38** | (alcohol NEAR/3 (dependen* or drink* or intoxicat* or abus* or misus* or risk* or consum* or excess* or reduc* or intervention*)):ti OR (alcohol NEAR/3 (dependen* or drink* or intoxicat* or abus* or misus* or risk* or consum* or excess* or reduc* or intervention*)):ab in Trials |
|  | **#39** | (drink* NEAR/3 (excess or heavy or heavily or harm or harmful or hazard* or risky or binge or harmful or problem*)):ti OR (drink* NEAR/3 (excess or heavy or heavily or harm or harmful or hazard* or risky or binge or harmful or problem*)):ab in Trials |
|  | **#40** | MeSH descriptor: [Drug Misuse] explode all trees |
|  | **#41** | MeSH descriptor: [Alcohol-Related Disorders] explode all trees |
|  | **#42** | MeSH descriptor: [Amphetamine-Related Disorders] this term only |
|  | **#43** | MeSH descriptor: [Cocaine-Related Disorders] explode all trees |
|  | **#44** | MeSH descriptor: [Inhalant Abuse] this term only |
|  | **#45** | MeSH descriptor: [Marijuana Abuse] this term only |
|  | **#46** | MeSH descriptor: [Marijuana Use] this term only |
|  | **#47** | MeSH descriptor: [Substance Abuse, Intravenous] this term only |
|  | **#48** | MeSH descriptor: [Substance Abuse, Oral] this term only |
|  | **#49** | #36 OR #37 OR #38 OR #39 OR #40 OR #41 OR #42 OR #43 OR #44 OR #45 OR #46 OR #47 OR #48 in Trials |
| **Parent terms** | **#50** | (parent or parents or parental):ti OR (parent or parents or parental):ab in Trials |
|  | **#51** | (mother? or mom? or mum? or father? or dad?):ti OR (mother? or mom? or mum? or father? or dad?):ab in Trials |
|  | **#52** | (pregnant or pregnancy or postpartum or paternal):ti OR (pregnant or pregnancy or postpartum or paternal):ab in Trials |
|  | **#53** | (family or families):ti OR (family or families):ab in Trials |
|  | **#54** | MeSH descriptor: [Parent-Child Relations] explode all trees |
|  | **#55** | MeSH descriptor: [Parenting] this term only |
|  | **#56** | MeSH descriptor: [Maternal Deprivation] this term only |
|  | **#57** | MeSH descriptor: [Paternal Deprivation] this term only |
|  | **#58** | MeSH descriptor: [Military Family] this term only |
|  | **#59** | MeSH descriptor: [Nuclear Family] explode all trees |
|  | **#60** | MeSH descriptor: [Single-Parent Family] explode all trees |
| **Combo** | **#61** | #50 OR #51 OR #52 OR #53 OR #54 OR #55 OR #56 OR #57 OR #58 OR #59 OR #60 in Trials |
|  | **#62** | #17 0R #35 OR #49 in Trials |
|  | **#63** | **#62 AND #61 in Trials** |

**Appendix E – Data extraction form**

| **Study summary** | |
| --- | --- |
| **Data extraction variable** | **Description** |
| **Author(s)** | Authors names. |
| **Date** | Date the paper was published. |
| **Design (RCT, cluster RCT, pilot RCT)** | Indicate whether the study was an individual RCT, cluster RCT, pilot RCT. |
| **No. of study conditions** | How many study conditions the RCT employed (n). |
| **Country RCT takes place** | What country the RCT takes place. For the U.S., please also indicate the state (where possible). |
| **Intervention** | Information about what type of intervention was under examination. Details how the authors' describe this (e.g., home visiting, peer-mentoring, CBT, family counselling, family therapy etc.). |
| **Control/Comparison** | Information about control/comparison group involved. As much detail as possible here is provided here and if there was more than one control group, all control groups are described. |
| **Recruitment setting** | Information about where the study recruited participants from (e.g., hospitals, local community centre, domestic violence shelter, head start etc.). |
| **Study sample** | Information about whether the study sample were parents at risk of DV, MH or SU; parents experiencing DV, MH or SU; children in the context of parental DV, MH or SU. |
| **Primary study aim** | The main aim of the study (which may be different to the intervention aim). |
| **Inclusion criteria** | Full details of the authors' inclusion criteria. |
| **Exclusion criteria** | Full details of the authors' exclusion criteria. |
| **Definition of DV/MH/SU** | Details of the authors' definition of DVA/MH/SU (if provided) or how they have conceptualised/operationalised DV/MH/SU (e.g., 'DVA operationalised as physical violence against a current intimate partner'). |
| **Theoretical link between DV/MH/SU** | Details of how the authors believe DVA/MH/SU to be related to one another. This is normally found in the introduction. |
| **Definition of exposure to DV/MH/SU (where targeting children)** | Details of the authors' definition of child exposure to DV/MH/SU (if provided). |
| **Number of participants that declined to take part in study (and reasons)** | The number of participants that declined to take part in the study (and the reasons why, if specified). |
| **Number of participants allocated to intervention** | The number of participants randomly allocated to the intervention group. |
| **Number of participants allocated to control** | The number of participants randomly allocated to the control group. |
| **Parent age - mean (SD) (overall and split by intervention/control)** | Mean age (and SD) of parents - overall and by intervention and control. |
| **Parent gender (overall and split by intervention/control)** | Percentage of female parents – overall and by intervention and control. |
| **Parent ethnicity (overall and split by intervention/control)** | Details on parents' ethnicity including n or % - overall sample and split by intervention and control, if possible. |
| **Child age - mean (SD) (overall and split by intervention/control)** | Mean age (and SD) of children - overall and by intervention and control. |
| **Child gender (overall and split by intervention/control)** | Percentage of female children – overall and by intervention and control. |
| **Child ethnicity (overall and split by intervention/control)** | Details on children’s ethnicity including n or % - overall sample and split by intervention and control, if possible. |
| **Socio-economic status** | Percentage of study sample experiencing low-income, less than high school education, or unemployment. |
| **Primary outcome measures** | What the authors report as their primary outcome measures (e.g., depression, mental health, and substance misuse). |
| **Secondary outcome measures** | What the authors report as their secondary outcome measures (e.g., depression, mental health, and substance misuse). |
| **Additional RCT follow-up papers** | Details on any additional follow-up papers published as part of the RCT. |
| **Other papers related to RCT** | Details on any other papers associated with this RCT. This includes papers examining moderators, mediators, process evaluation, qualitative data. |
| **Intervention** | |
| **Data extraction variable** | **Description** |
| **Intervention name** | Name of the intervention if the authors' give a name. |
| **Intervention type (secondary prevention, tertiary prevention, treatment)** | Whether the intervention is secondary prevention, tertiary prevention, or treatment. |
| **Intervention primary aim** | The primary aim of the intervention as described by the authors. |
| **Intervention description (what does it involve)** | Details on what the intervention involves as described by the authors. |
| **Intervention theoretical underpinnings (why)** | Details on the theoretical underpinnings of the intervention as described by the authors. |
| **Intervention recipient (e.g., parent and child, whole family, parent etc.)** | Information on the intended intervention recipient. |
| **Intervention provider (who delivers the intervention)** | Information on who delivers the intervention. |
| **Intervention provider skills, expertise, relationship with intervention participants** | Full details of the intervention provider skills, expertise, and relationship with intervention participants. |
| **Mode of delivery (face-to-face; internet; telephone etc.)** | The mode of delivery for the intervention (e.g., face-to-face, online, phone). |
| **Setting (where intervention delivered)** | Information on where the intervention is delivered (e.g., home, clinic, online, over the phone, in the community). |
| **Duration** | The duration of the intervention in months (or days if shorter than one month). |
| **Number of sessions** | Number of sessions the intervention involves. Split by intervention recipient if necessary. |
| **Frequency of sessions** | Information on the frequency of these sessions. Split by intervention recipient if necessary. |
| **Length of sessions** | Information on the length of the sessions in minutes. Split by intervention recipient if necessary. |
| **Tailoring (tailored for individual?)** | Information on whether the intervention is tailored in any way to meet individuals' needs. Split by intervention recipient if necessary. |
| **Modifications for parents** | Details on whether there any modifications made to the intervention to specifically cater for parents. |
| **PPI used to develop intervention** | Whether the intervention was developed using PPI (yes/no). |
| **Deviations from intended intervention parameters** | Details on whether there are any ways in which the intervention planned to be delivered within this study (as described above) that deviates from the interventions intended parameters (e.g., intervention as described in the fields above has been used with parents but was originally designed for older adults). Description of how any deviations were described/taken into account. |
| **Fidelity to model** | Details on whether the intervention was delivered as planned in the authors' opinion. |
| **Authors thoughts on key components of the intervention** | Details on what authors suggest the key components of the intervention are. |
| **Authors thoughts on impact of participant engagement** | Details on whether authors believe that engagement or lack of engagement in the intervention has impacted the results of the study. |
| **Authors thoughts on impact of fidelity to model** | Details on whether authors believe that fidelity to model has impacted the results in any way. |
| **Associated intervention paper/manual** | Link to any associated intervention description paper/manual. |
| **Outcomes** | |
| **Data extraction variable** | **Description** |
| **Data collection time points** | Information on data collection time points, including baseline. |
| **Domestic violence outcomes (parent)** | Information on what DVA outcomes were measured and at what time points. If authors have derived categorical variable from measure this is described here. |
| **Summary of results** | Summary of the DVA outcome measure results. Details on N, mean, SD, %, SE, CI, p-value, or any other relevant statistical information. |
| **Mental health outcomes (parent)** | Information on what MH outcomes were measured and at what time points. If authors have derived categorical variable from measure this is described here. |
| **Summary of results** | Summary of the MH outcome measure results. Details on N, mean, SD, %, SE, CI, p-value, or any other relevant statistical information. |
| **Substance misuse outcomes (parent)** | Information on what SU outcomes were measured and at what time points. If authors have derived categorical variable from measure this is described here. |
| **Summary of results** | Summary of the SU outcome measure results. Details on N, mean, SD, %, SE, CI, p-value, or any other relevant statistical information. |
| **Other outcomes (parent)** | List of any other outcomes that were measured in relation to the parent. |
| **Summary of results** | Brief summary of the results related to other parent outcomes as reported by authors. |
| **Externalising behaviour outcomes (child)** | Information on what externalising behaviour outcomes were measured and at what time points. If authors have derived categorical variable from measure this is described here. |
| **Summary of results** | Summary of the externalising behaviour outcome measure results. Details on N, mean, SD, %, SE, CI, p-value, or any other relevant statistical information. |
| **Internalising behaviour outcomes (child)** | Information on what internalising behaviour outcomes were measured and at what time points. If authors have derived categorical variable from measure this is described here. |
| **Summary of results** | Summary of the internalising behaviour outcome measure results. Details on N, mean, SD, %, SE, CI, p-value, or any other relevant statistical information. |
| **Substance misuse outcomes (child)** | Information on what child SU outcomes were measured and at what time points. If authors have derived categorical variable from measure this is described here. |
| **Summary of results** | Summary of the child SU outcome measure results. Details on N, mean, SD, %, SE, CI, p-value, or any other relevant statistical information. |
| **Other outcomes (child)** | List of any other outcomes that were measured in relation to the child. |
| **Summary of results** | Brief summary of the results related to other child outcomes as reported by authors. |
| **Family outcomes** | List of any family outcomes that were measured. |
| **Summary of results** | Brief summary of the results related to other family outcomes as reported by authors. |
| **Analysis technique employed (and does this consider interaction between DV, MH and SU?)** | Information on whether the analysis considered the interaction between DVA, MH and SU. |
| **Moderator analysis** | Whether any moderator analysis was conducted (yes/no). |
| **Moderator analysis results** | Information on results from moderator analysis. |
| **Author conclusions about intervention effectiveness** | Authors’ conclusions about study from abstract and discussion. |
| **Author conclusions about intervention effectiveness** | Summary of authors conclusions about intervention effectiveness. |
| **Authors recommendations for future research** | Summary of authors recommendations for future research. |
| **Authors recommendations for practice** | Summary of authors recommendations for practice. |

**Appendix F – Study population and SES**

| **Studies measuring DVA and MH** | | | | |
| --- | --- | --- | --- | --- |
| **Study** | **Low income % (defined as low income, or receiving welfare benefits)** | **Education % (defined as less than high school education)** | **Unemployed %** | **Notes** |
| 4. El-Mohandes et al. (2008)  U.S. | 79% | 31.1% | 64.5% | **Income** – percentage receiving Medicaid  **Employment** – worked out percentage employed and assumed the rest are ‘unemployed’. |
| 16. Nagle (2002)  U.S. | 100%  To take part all participants had to have low income (below 133% federal poverty level) | NR | 74.9% | **Income** – to take part, all participants had to have low income so have used this to populate this field.  **Education** – 10.6 years in education. |
| 29. Sullivan et al. (2002)  U.S. | 88% | NR | 66% | **Income** – percentage receiving governmental assistance |
| 30. Taft et al. (2011)  Australia | 72.9% | 49.6% | NR | **Income** – has health card which is a subsidy for those with inadequate income.  **Education** – Year 12 or less. |
| 31. Tiwari et al. (2005)  China | 15.45% | NR | Women - 61.9%  Partner - 28.2% | **Income** – under national average wage  **Employment** – worked out percentage of women and partners in paid work and then assumed the rest were unemployed. |
| 35. Zlotnick et al. (2011)  U.S. | 100%  To take part all participants had to have low income. | 25.9% | 33.3% | **Income –** to take part, all participants had to have low income so have used this to populate this field.  **Education** – some high school but did not graduate. |
| 36. Dinomohammadi et al. (2021)  Iran | NR | NR | NR | **Income** – report on economic class and social class but is not clear what numbers refer to i.e., whether ‘first’ economic class refers to low class or higher class. |
| 37. Skar et al. (2021)  Columbia | 100%  All participants were attending services for low-income families. | 5.7% | 19.9% | **Education** – all that were classed as no education or primary school. Did not include secondary school education in here. |
| **Studies measuring DVA and SU** | | | | |
| **Study** | **Low income % (defined as low income, or receiving welfare benefits)** | **Education % (defined as less than high school education)** | **Unemployed %** | **Notes** |
| 10. Jacobs et al. (2016)  U.S. | 59.4% | NR | NR | **Income** – experiencing financial difficulties. |
| 12. Lam et al. (2009)  U.S. | NR | NR | NR | **Income** – mean annual income in $1,000s = 34.6  **Education** – male and female mean years in education. Male - 12.9 years  Female - 13.6 years |
| 13. LeCroy et al. (2011)  U.S. | 90.3% | NR | 71.3% | **Income –** number receiving health insurance with Medicaid Arizona.  **Employment** – assumed those not employed were unemployed. |
| 25. Stover (2015)  U.S. | NR | NR | 38% | **Education** – 11.67 years fathers’ education.  **Employment** – assumed those that were not employed at least part-time were unemployed. |
| **Studies measuring MH and SU** | | | | |
| **Study** | **Low income % (defined as low income, or receiving welfare benefits)** | **Education % (defined as less than high school education)** | **Unemployed %** | **Notes** |
| 1. Cupples et al. (2010)   UK | 100%  Had to live in socio-economically deprived areas to be included in the study. | 10.8% | 48.7% | **Income** – only thing reported is someone in household owned a car (55.1%)  **Education** – no educational qualifications. **Also report on those with GCSEs and NVQs but this is high school completion therefore not less than high school.**  **Employment** - someone in household was unemployed. |
| 6. Fleming et al. (2008); Wilton et al. (2009)  U.S. | NR | 12.8% | 30.3% | **Employment** – not in labour force or currently seeking employment. |
| 7. Grigg (1994)  Canada | 17.6% | NR | Fathers - 24.1%  Mothers - 17.7% | **Income** - Struggling financially (below $19k per year) |
| 11. Jones et al. (2011)  U.S. | NR | NR | 67.3% | **Income –** only give mean income earned and income from public assistance.  **Education** – mean number of years in education  **Employment** – assuming if not employed then unemployed. |
| 14. Luthar et al. (2007)  U.S. | NR | 36.2% | 81.9% | **Employment** – unemployed or welfare. |
| 15. McWhirter (2011)  U.S. | NR  All participants residing in temporary family homeless shelter. | 35% | NR | **Education** – some high school. |
| 20. Rotheram-Borus et al. (2003)  U.S. | NR | NR | NR | **No information given on SES.** |
| 21. Rotheram-Borus et al. (2012)  U.S. | 63.1% | 67.6% | 28.4% | **Income –** barely paying bills or struggling to survive.  **Employment** – assume if not employed then unemployed. |
| 24. Wu and Slesnick (2019)  U.S. | 60.1% | NR | NR | **Income –** under poverty level for 2-person household ($17,420) conservative estimate as have only included anyone under $15k bracket. |
| 27. Suchman et al. (2010; 2011)  U.S. | NR | NR | 80.9% | **Education** – 12.3 mean years education. |
| 28. Suchman et al. (2017)  U.S. | NR | NR | 79.9% | **Education** – 12.4 mean years education. |
| 33. Volpicelli et al. (2000)  U.S. | NR | NR | NR | **Education** – 11.6 mean years education. |
| 34. Walkup et al. (2009)  U.S. | NR | NR* | NR* | **Education** – less than high school calculated as those who don’t have high school general equivalency diploma or some college.  *Percentages provided based on whole sample rather than those above the age of 16 therefore, NR. |
| **Studies measuring DVA, MH, and SU** | | | | |
| **Study** | **Low income % (defined as low income, or receiving welfare benefits)** | **Education % (defined as less than high school education)** | **Unemployed %** | **Notes** |
| 2. Duggan et al. (2007)  U.S. | 57.5% | 41.8% | 26.8% | **Income –** below poverty line.  **Education** – those that were not marked as graduated from high school have assumed didn’t graduate.  **Employment** – did not work in year prior to employment. |
| 3. Duggan et al. (1999; 2004); McFarlane et al., (2012)  U.S. | 64.7% | NR | 51.3% | **Income –** below poverty line.  **Employment** – did not work in year prior to employment. |
| 5. Fergusson et al. (2006; 2013)  New Zealand | 36.5% | Mothers – 70%  Fathers – 74.9% | NR | **Income** – family income inadequate or very inadequate. Also have percentage that are welfare dependent 89.2%  **Education** – no qualifications. |
| 8. Slesnick and Erdem (2013); Guo et al. (2016)  U.S. | NR  All mothers are homeless living in temporary living/shelters. | NR | 76.7% | **Income** – $300.9 mean personal monthly income.  **Education** – 11.75 highest level of education in years? |
| 9. Jack et al. (2019)  U.S. | 100%  All had to be living in poverty to participate. | 46.7% | 77.1% | **Income** – all participants had to be living in poverty to participate.  **Education** – assumed didn’t graduate from high school if not indicated.  **Employment** – assumed unemployed if did not mark as employed. |
| 17. Olds et al. (2004)  U.S. | 100%  All had to be low income to participate. | NR | NR | **Education** – only report years completed. |
| 18. Olds et al. (2007; 2010; 2019)  U.S. | 100%  All had to be low income to participate. | NR | 54.9% | **Education** – in years and therefore, not reported.  **Employment** – head of household unemployed. Assumed if not recorded as employed then unemployed. |
| 19. Ondersma et al. (2017)  U.S. | 93.6% | 24% | 51.2% | **Income** – percentage receiving “Public assistance” which refers to receipt of food stamps: Women, Infants, and Children food supplements; Temporary Assistance for Needy Families; or housing assistance.  **Percentages based on intervention and active control group.** |
| 22. Rotheram-Borus et al. (2015)  South Africa | 100%  All had to be low income to take part in the study. | 75% | 79% |  |
| 23. Silovsky et al. (2011)  U.S. | NR | 22.9% | 44.8% |  |
| 26. Stover et al. (2019)  U.S. | NR | NR | 54% | **Employment** – assumed if not recently employed then unemployed. |
| 32. Trevillion et al. (2020)  UK | 15.56% | 15.09% | 7.55% | **Income** - <£15k taken as low income given that under £17k would be considered low income for two adults in household.  **Education** - None/only school qual. |

**NB.** **Orange coloured cells** indicate that % is currently above the national average for that country. **Blue coloured cells** indicate that % is currently below national average for that country.

**Appendix G – Intervention types**

There were a range of family focused intervention types which we categorised as follows; home visiting or parenting (n = 11), home visiting or parenting supplements (n = 5), therapy (n = 11), multi-component (n = 5), coping skills (n = 2), empowerment/advocacy (n = 3), and brief alcohol interventions (n = 1). These are described in more detail below.

**Home visiting and parenting interventions** were designed to reduce child maltreatment through promoting positive parenting practices and, in most cases, good maternal health and well-being (including reducing DVA/MH/SU). They involved regular home visits (or telephone check-ins) from nurses, social workers, or paraprofessionals who provided ongoing support in the home setting for at-risk mothers or families from pregnancy/birth through to early childhood (duration ranging from 7.5 months – 3-5 years; one study did not state duration). One intervention involved 12 sessions delivered in child centres by trained professionals who provided parenting support for parents of young children (duration not stated) [15]. The support provided (and description of this) varied between studies but tended to include elements such as crisis management, education and role modelling of positive parenting practices, education on child health and development, and, in some cases, development of family-initiated goals and support plans. Home visiting and parenting interventions aimed to prevent/reduce parental DVA/MH/SU by increasing parents’ knowledge of DVA/MH/SU and access to community services/resources for these issues. This was done by training the professionals/paraprofessionals to identify parental DVA/MH/SU and, where identified, refer parents to relevant community services. However, in some cases it involved parental education on DVA/MH/SU and, in one, the intervention targeted DVA/MH through parenting and strengthening the parent-child relationship alone [15]. Consequently, although home visiting interventions provided integrated support for these three issues, they treated these three public health issues as co-occurring: offering separate education, support, and referrals for DVA/MH/SU and not acknowledging the relationships between them. Ten studies examined home visiting and parenting interventions, some of which were delivered by professionals [15-18] and others, paraprofessionals [19-24].

One additional study examined a home visiting intervention specifically designed to reduce alcohol use and HIV related behaviours in mothers in South Africa [25]. This home visiting intervention differed from the others in its focus and content; providing specific education for mothers on general maternal and child health, HIV/TB, alcohol use, MH, nutrition, and dealing with crises as well as a brief alcohol intervention which highlighted the impact of alcohol use on child health and development and the mothers’ current alcohol use. It aimed to reduce alcohol use (and subsequently MH/DVA) by increasing knowledge of alcohol use and MH, increasing knowledge of the consequences of alcohol use, and increasing capabilities in terms of goal setting and problem solving. This intervention was delivered by paraprofessionals in the home setting over a period of 18 months.

**Home visiting and parenting supplements** were implemented within already established parenting and home visiting interventions (normally U.S. based) such as the Nurse Family Partnership (NFP), Healthy Families America (HFA), or SafeCare (as per description above). They involved the addition of additional training, resource, or intervention components in order to target DVA/MH/SU more effectively and are normally delivered to and/or by professionals/paraprofessionals in the home setting. However, in one case, the intervention was delivered within a child centre [15]. The duration of home visiting and parenting supplements varied between 6 months – 2-3 years (one study did not state the duration but indicated this was under 6 months [26] and another did not state the duration [15]). Interventions in this group targeted DVA/MH/SU in different ways, tending to treat these issues as co-occurring or uni-directional. A total of five studies examined the effectiveness of home visiting supplements [15, 26-29].

Jack et al. [27] implemented a tailored multicomponent IPV intervention within a pre-existing home visiting intervention to better reduce the risk of IPV and improve mother quality of life. They targeted IPV by empowering mothers, helping them to develop new coping and safety planning skills, and increasing their self-efficacy whilst targeting MH and SU by increasing mothers’ use of community services. Two studies employed motivational interviewing to better target DVA/MH/SU within the home visiting model; Ondersma et al. [28] through an e-intervention delivered to mothers on a tablet during home visits and Silovsky et al. [26] through additional training for home visitors in the identification of DVA/MH/SU and motivational interviewing techniques. Nagle (29) aimed to better strengthen the mother-child relationship and parenting in the context of parental DVA/MH/SU by introducing a child MH professional into the home visiting team who provided support for the team and MH support to mothers. Finally, Skar et al. [15] aimed to better target household violence by adding a violence curriculum to a parenting intervention for parents of young children living with low-income in areas characterised by high levels of violence.

**Therapy interventions** had a range of different aims including reducing already established DVA, MH, or SU and/or promoting parenting in the context of these issues. Therapy interventions included individual, dyadic, family, and group therapies which drew on one or more therapeutic approaches including interpersonal and relational psychotherapy, behavioural couples therapy, experiential systemic therapy, cognitive behavioural therapy (CBT), substance abuse and domestic violence CBT (SADV), family systems therapy, mentalisation- and representation- focused therapy, goal-orientated therapy, emotion-focused therapy, attachment-focused therapy, and solution-focused counselling. Therapy interventions mostly targeted mothers and fathers experiencing SU (child age ranging from 0-16 years), however some targeted pregnant women or mothers at risk of, or experiencing, MH or DVA. The duration of therapy interventions ranged from around 6 weeks to a maximum of six months, and they were delivered by trained therapists who often had specific experience working with DVA, MH, SU, families, or children. In one case, the intervention was delivered by a researcher [30]. The methods for addressing DVA, MH, and SU varied across studies, and some involved also working with the child. Therapy interventions most often employed a uni-directional approach to addressing DVA, MH and/or SU, however, some used approaches which treated these issues as bi-directional or co-occurring. There were ten studies that involved therapy interventions as described above [30-39].

One additional therapy intervention involved guiding parents through a self-help information to help reinforce learning [40]. This was delivered by Psychological Wellbeing Practitioners (PWPs) within IAPT, NHS and was delivered over a period of around 6 weeks. This intervention treated DVA/MH/SU as uni-directional issues.

**Multi-component interventions** predominately aimed to reduce SU, engage parents in SU treatment, or promote parenting within this context. However, one study aimed to reduce risk of DVA and MH for at-risk mothers [41]. Multi-component interventions provided support to parents and/or children using two or more approaches. These approaches included things such as motivational interviewing, therapy (e.g., CBT, couples’ behavioural therapy), empowerment-based interventions, parenting-skills interventions, case management, financial support, housing support etc. Parents/children may receive different aspects of the intervention depending on their specific needs, be given the flexibility to access those they wish to or be required to engage with all aspects of the intervention. Multicomponent interventions tended to be delivered to pregnant women, mothers or fathers (child age ranging from 0-12 years) by one or multiple therapists/counsellors and all aspects of the intervention were normally delivered within the same setting and hosted by the same host organisation. The duration of multicomponent interventions ranged from 12 weeks to 6 months, and one study didn’t disclose the intervention duration. These studies conceived DVA, MH, and SU in a range of ways including co-occurring, uni-directional, and bi-directional. Five studies considered the effectiveness of multicomponent interventions in targeting a combination of DVA/MH/SU [41-45].

**Coping skills interventions** primarily focused on increasing parents and/or children’s coping skills in order to indirectly reduce DVA, MH, and SU. Only two interventions solely employed this approach; both of which were linked and focused on helping parents and children (age ranging from 6-20 years) develop coping skills around parents’ HIV status [46, 47]. Both interventions involved a mothers/parents only group and a mother/parent and child group. The former involved discussing coping skills related to HIV status, ways to improve MH and reduce SU, and decisions on disclosing HIV status to children and custody plans. The latter involved the same but without a focus on custody plans due to the changing profile of HIV. The duration of these interventions ranged from 8 weeks to 2 years, and they were delivered by social workers, graduates, or other professionals. Both conceived MH and SU as co-occurring.

**Empowerment/advocacy interventions** aimed to empower women experiencing IPV/DVA by providing them with information on domestic violence, safety, choice making, and problem-solving, and helping families to access and mobilise critical community-based services to meet their basic needs. This included helping families access and utilise legal assistance, housing, employment, education, childcare, and social support (etc.), providing them with support in arranging, attending, travelling to, and/or completing paperwork for meetings, where necessary. There were three studies within this review that examined family focused empowerment/advocacy interventions.

Tiwari et al. [48] delivered a one-off culturally informed empowerment intervention to provide Chinese pregnant women within an opportunity to disclose abuse within a non-judgemental environment, increasing their knowledge of DVA, increase their skills in safety, choice making and problem solving, and help change their beliefs in the behaviour. Two other studies focused predominately on assessing family’s needs and mobilising critical community-based services to address these. Taft et al. [49] provided pregnant women or mothers with children under 5 years of age with advocacy-based support, parenting support and general be-friending. This was delivered by paraprofessionals in the home setting over a period of 12 months. Sullivan et al. [50] provide advocacy-based support to both mothers and children aged between 7-11 years (as well as a child’s education support group) and have a focus on transferring advocacy skills back to the mother. All these interventions viewed DVA and MH as bi-directional. Although there were only a few interventions that fell into this category, many were informed by empowerment theory.

**Brief alcohol interventions.** One study targeted pregnant women’s high-risk alcohol use through a brief alcohol intervention [51]. The brief intervention involved providing psychoeducation on the effects of alcohol (in terms of impact on mother and baby), meeting mothers at their stage of change, and utilising motivational interviewing, CBT, homework, and follow-up sessions to encourage a reduction in alcohol use (duration 8 weeks). Consequently, the intervention targeted alcohol use by increasing mothers’ knowledge of the impact of alcohol use on themselves and their unborn child, increasing skills in self-monitoring and self-regulation of alcohol use, and providing social support to enable change. As a result, Fleming et al. [51] hypothesise this might also lead to positive changes on a broader array of outcomes, including pregnant women’s MH; treating these issues as uni-directional. The intervention was delivered predominately by clinic nurses as two sessions (one brief session and one reinforcement session) in an obstetric clinic. Each of these sessions were followed by a follow-up telephone call to reinforce learning and discuss any challenges.

**Appendix H – Quality appraisal results**

| **Study** | **Outcome** | **1** | **1b** | **2** | **3** | **4** | **5** | **Overall** | **Notes** |
| --- | --- | --- | --- | --- | --- | --- | --- | --- | --- |
| **1.** Cupples et al. | General MH | L | N/A | L | L | SC | SC | SC | Missing outcome data which could depend on true value (drug only), self-report measures used, do not use a valid and reliable measure for SU, and no publicly available pre-specified data analysis plan. |
|  | Drug | - | - | - | H | SC | SC | H |  |
|  | Alcohol | - | - | - | L | SC | SC | SC |  |
| **2.** Duggan et al. | Victimisation/ perpetration | L | N/A | L | H | SC | SC | H | Missing outcome data that could have depended on true value, self-report measures used, do not use a valid and reliable measure for SU, no publicly available pre-specified analysis plan. |
|  | Depression | - | - | - | H | SC | SC | H |  |
|  | General MH | - | - | - | H | SC | SC | H |  |
|  | Alcohol | - | - | - | H | SC | SC | H |  |
|  | Drug | - | - | - | H | H | SC | H |  |
| **3.** Duggan et al. | Victimisation/ perpetration | SC | N/A | L | L | SC | SC | SC | Differences between groups in key outcomes (DVA and general MH) at baseline, missing data not accounted for (SU), self-report data measures, do not use valid and reliable measure for SU, and no publicly available pre-specified data analysis plan. |
|  | Depression | - | - | - | L | SC | SC | SC |  |
|  | General MH | - | - | - | L | SC | SC | SC |  |
|  | Alcohol | - | - | - | SC | SC | SC | SC |  |
|  | Drug use | - | - | - | SC | H | SC | H |  |
| **4.** El-Mohandes et al. | Victimisation | L | N/A | L | L | SC | SC | SC | Self-report measures used and no publicly available pre-specified analysis plan. |
|  | Depression | - | - | - | L | SC | SC | SC |  |
| **5.** Fergusson et al. | Victimisation | L | N/A | L | L | SC | SC | SC | Self-report measures used and no publicly available pre-specified data analysis plan. |
|  | Depression | - | - | - | L | SC | SC | SC |  |
|  | Alcohol | - | - | - | L | SC | SC | SC |  |
|  | Drug | - | - | - | L | H | SC | H |  |
| **6.** Fleming et al. | Depression | L | N/A | L | SC | L | SC | SC | Missing outcome data that hasn’t been accounted for (MH), and unclear whether MH analysis was part of pre-specified analysis plan. |
|  | Alcohol use | - | - | - | L | L | SC | L |  |
| **7.** Grigg | General MH | L | N/A | SC | SC | SC | SC | H | Do not include all participants in the analysis (remove those that dropped out), missing data not accounted for, self-report measures used, no pre-specified data analysis plan. |
|  | General MH | - | - | - | SC | SC | SC | H |  |
|  | Alcohol | - | - | - | H | SC | SC | H |  |
| **8.** Slesnick et al. | Victimisation | L | N/A | SC | L | SC | SC | SC | Differential attrition in intervention and control groups but unlikely to have impacted outcome, most measures use self-report, and no pre-specified data analysis plan. |
|  | Depression | - | - | - | L | SC | SC | SC |  |
|  | General MH | - | - | - | L | SC | SC | SC |  |
|  | Alcohol and drug use | - | - | - | L | L | SC | SC |  |
| **9.** Jack et al. | Victimisation | SC | L | L | SC | SC | SC | SC | Not enough detail on randomisation and whether recruitment started before or after this, not clear how accounted for missing outcome data, and no publicly available protocol referenced, but a protocol does exist. |
|  | PTSD | - | - | - | SC | SC | SC | SC |  |
|  | Depression | - | - | - | SC | SC | SC | SC |  |
|  | General MH | - | - | - | SC | SC | SC | SC |  |
|  | Alcohol | - | - | - | SC | SC | SC | SC |  |
|  | Drug | - | - | - | SC | SC | SC | SC |  |
| **10.** Jacobs et al. | Victimisation and perpetration | L | N/A | L | SC | SC | SC | SC | No analysis done to account for missing data, do not reference publicly available data analysis plan. |
|  | Alcohol, drug, and marijuana | - | - | - | SC | SC | SC | SC |  |
| **11.** Jones et al. | Depression | SC | N/A | SC | L | L | SC | SC | No information on how randomisation was conducted, no information on adherence, self-report measures used (although validate SU using urine toxicology), do not reference publicly available pre-specified data analysis plan. |
|  | Alcohol, heroin, cocaine, and drug use | - | - | - | L | L | SC | SC |  |
| **12.** Lam et al. | Victimisation/ perpetration | SC | N/A | L | L | SC | SC | SC | Not enough information on randomisation process, self-report measures used, and do not reference publicly available pre-specified data analysis plan. |
|  | Alcohol | - | - | - | L | SC | SC | SC |  |
| **13.** Lecroy et al. | Victimisation | H | N/A | SC | SC | H | SC | H | Not enough info on randomisation process and baseline differences between groups, no information on adherence to intervention/control, do not account for missing data, do not use valid and reliable measures for DVA or SU, no publicly available protocol referenced but a protocol does exist. |
|  | Alcohol | - | - | - | SC | H | SC | H |  |
| **14.** Luthar et al. | Depression | SC | N/A | L | H | SC | SC | H | Differences in baseline data, missing outcome data that could have depended on true value, and lack of pre-specified data analysis plan. |
|  | Opiate | - | - | - | H | L | SC | H |  |
|  | Cocaine | - | - | - | H | L | SC | H |  |
| **15.** McWhirter | Depression | SC | N/A | L | L | L | SC | SC | Not enough information provided on baseline differences, do not use valid and reliable measure for SU, and no publicly available protocol referenced but a protocol does exist. |
|  | Alcohol | - | - | - | L | H | SC | H |  |
| **16.** Nagle | Victimisation and perpetration | L | N/A | L | SC | SC | SC | SC | Missing data not accounted for, use of self-report measures, and no publicly available pre-specified data analysis plan/protocol. |
|  | Depression | - | - | - | SC | SC | SC | SC |  |
| **17.** Olds et al. | Victimisation | L | N/A | L | SC | SC | SC | SC | Missing data not account for however, do control for baseline characteristics, self-report measures used, general MH measure not described, no valid and reliable measure used for SU, and no publicly available pre-specified data analysis plan mentioned. |
|  | General MH | - | - | - | SC | H | SC | H |  |
|  | Alcohol and marijuana | - | - | - | SC | H | SC | H |  |
| **18.** Olds et al. | Victimisation | L | N/A | L | L | SC | SC | SC | Missing data not accounted for, self-report measures used, no valid and reliable measure used for SU, no publicly available pre-specified data analysis plan mentioned. |
|  | Depression |  |  |  | SC | SC | SC | SC |  |
|  | Anxiety |  |  |  | SC | SC | SC | SC |  |
|  | Substance use |  |  |  | SC | H | SC | H |  |
| **19.** Ondersma et al. | Victimisation and perpetration | L | N/A | L | L | SC | SC | SC | Self-report measures, and no publicly available pre-specified data analysis plan mentioned. |
|  | Depression | - | - | - | L | SC | SC | SC |  |
|  | Alcohol | - | - | - | L | SC | SC | SC |  |
|  | Drug | - | - | - | L | L | SC | SC |  |
| **20.** Rotheram-Borus | General MH | SC | N/A | L | SC | SC | SC | SC | Not enough info on how randomisation was conducted, no clear information on missing data and do not do any multiple imputation (include baseline characteristics as co-variates though), self-report measures used and no valid and reliable measure used for SU, no publicly available pre-specified data analysis plan mentioned. |
|  | Substance use | - | - | - | SC | H | SC | H |  |
| **21.** Rotheram-Borus | General MH | SC | N/A | L | L | SC | SC | SC | Differences at baseline including in SU, self-report measures used and do not make it clear how SU has been measured, no publicly available pre-specified data analysis plan mentioned. |
|  | Alcohol, marijuana, and drug | - | - | - | L | H | SC | H |  |
| **22.** Rotheram-Borus | Victimisation | L | SC | L | SC | H | SC | H | Not enough information on cluster randomisation process, missing outcome data that they do not account for, self-report measures and do not use valid and reliable measures for DVA and SU, and no publicly available pre-specified data analysis plan mentioned. |
|  | Depression | - | - | - | SC | SC | SC | SC |  |
|  | Alcohol | - | - | - | SC | H | SC | H |  |
| **23.** Silovsky et al. | Victimisation | L | N/A | H | L | SC | SC | H | Differential uptake in intervention and control likely to be due to those delivering intervention and control groups, self-report measures used, no publicly available pre-specified data analysis plan referenced. |
|  | Depression | - | - | - | L | SC | SC | H |  |
|  | Alcohol and drug use | - | - | - | L | SC | SC | H |  |
| **24.** Wu and Slesnick | Depression | SC | N/A | L | L | SC | SC | SC | Not enough information provided on randomisation process, self-report measures used, no pre-specified data analysis plan |
|  | Alcohol, marijuana, and drug | - | - | - | L | SC | SC | SC |  |
| **25.** Stover | Perpetration and victimisation | L | N/A | H | H | SC | H | H | Did not sig differ on any baseline characteristics but do not provide data for us to examine ourselves, no information on whether used intention-to-treat analysis, doesn’t report on missing data or any analysis done to account for this, self-report measures used, no publicly available pre-specified data analysis plan mentioned and do not report on TLFB-SV measure they used for DVA (only report on CTS2). |
|  | Substance use | - | - | - | H | SC | SC | H |  |
| **26.** Stover et al. | Perpetration | L | N/A | H | L | SC | SC | H | Deviations arose due to trial context with intervention group not receiving as many sessions because they were discharged from residential SU early, self-report data used, no pre-specified data analysis plan mentioned. |
|  | General MH | - | - | - | L | SC | SC | H |  |
|  | Substance use | - | - | - | L | SC | SC | H |  |
| **27.** Suchman et al. | Depression | SC | N/A | L | L | L | SC | SC | Not enough information given about randomisation so unable to assess and no publicly available pre-specified data analysis plan. |
|  | General MH | - | - | - | L | L | SC | SC |  |
|  | Drug | - | - | - | L | L | SC | SC |  |
| **28.** Suchman et al. | Depression | SC | N/A | L | L | SC | SC | SC | Not enough information given about randomisation so unable to assess, self-report measures used, and no publicly available pre-specified data analysis plan. |
|  | General MH | - | - | - | L | SC | SC | SC |  |
|  | Heroin, opioid, cocaine | - | - | - | L | SC | SC | SC |  |
| **29.** Sullivan et al. | Victimisation | SC | N/A | L | SC | H | SC | H | Sig differences at baseline in terms of witnessing abuse, involvement in proactive services, parenting support, and reported drug use. Missing data accounted for using expectation maximisation techniques, but this is not as desirable as multiple imputation or maximum likelihood methods. No valid and reliable measure for DVA and no publicly available pre-specified data analysis plan. |
|  | Depression | - | - | - | SC | SC | SC | H |  |
| **30.** Taft et al. | Victimisation | H | H | L | L | SC | L | H | Randomisation was not concealed from clusters/participants and therefore, recruitment to these was affected resulting in fewer than anticipated numbers recruited to the control group in particular. Self-report measures also used. |
|  | Depression | - | - | - | L | SC | L | H |  |
|  | General MH | - | - | - | L | SC | L | H |  |
| **31.** Tiwari et al. | Victimisation | L | N/A | L | L | SC | SC | SC | Self-report measures used and no publicly available pre-specified data analysis plan. |
|  | Depression | - | - | - | L | SC | SC | SC |  |
|  | General MH | - | - | - | L | SC | SC | SC |  |
| **32.** Trevillion et al. | Victimisation | L | N/A | L | L | SC | L | L | Self-report measures used. |
|  | Depression (EPDS) | - | - | - | L | SC | L | L |  |
|  | Depression (PHQ-9) | - | - | - | L | SC | L | L |  |
|  | Anxiety | - | - | - | L | SC | L | L |  |
|  | Alcohol | - | - | - | L | SC | L | L |  |
| **33.** Volpicelli et al. | General MH | SC | N/A | L | SC | SC | SC | H | Some differences in key prognostic factors (child maltreatment histories), missing outcome data not accounted for, self-report measured used, and no publicly available pre-specified data analysis plan. |
|  | Cocaine (ASI) | - | - | - | SC | SC | SC | H |  |
|  | Cocaine (urinanalysis) | - | - | - | L | SC | SC | H |  |
| **34.** Walkup et al. | Depression | L | N/A | SC | H | SC | SC | H | There was differential attrition across intervention and control groups and there were high levels of missing outcome data which was not accounted for. Self-report measures used, no valid and reliable measure of SU, and no publicly available pre-specified data analysis plan. |
|  | Alcohol and substance use | - | - | - | H | H | SC | H |  |
| **35.** Zlotnick et al. | Victimisation | SC | N/A | L | L | SC | SC | SC | Some baseline differences between groups in terms of prognostic factors (child maltreatment history), self-report measures used, and no publicly available pre-registered data analysis plan. |
|  | Depression (LIFE) | - | - | - | L | SC | SC | SC |  |
|  | Depression (EPDS) | - | - | - | L | SC | SC | SC |  |
|  | PTSD (LIFE) | - | - | - | L | SC | SC | SC |  |
|  | PTSD (Davidson Trauma Inventory) | - | - | - | L | SC | SC | SC |  |
| **36.** Dinmohammadi et al. | Victimisation | L | N/A | H | H | SC | H | H | Inappropriate method used to examine effect of assignment to intervention (per protocol analysis), and this could have impacted results, do not account for missing outcome data, self-report measures used, no publicly available pre-registered data analysis plan, and bias in selection of reported results for victimisation (do not present follow-up data). |
|  | General MH | - | - | - | H | SC | H | H |  |
| **37.** Skar et al. | Victimisation | L | N/A | H | L | SC | SC | H | Inappropriate method used to examine effect of assignment to intervention (per protocol analysis), and this could have impacted results, self-report measures used, and no publicly available pre-registered data analysis plan. |
|  | Perpetration | - | - | - | L | SC | SC | H |  |
|  | General MH | - | - | - | L | SC | SC | H |  |

**Appendix I – Study outcomes tables and harvest plots for sub-categories of DVA, MH, and SU outcomes**

| **Study outcomes for studies measuring DVA and MH** | | | | | | | | | |
| --- | --- | --- | --- | --- | --- | --- | --- | --- | --- |
| **Study number** | **Timepoint** | **Overarching construct** | **Measure** | **Authors’ reported results** | **Cohen’s d** | **95% CI – lower** | **95% CI – upper** | **Direction of effect** | **Combined impacts** |
| 4 | Post intervention | Victimisation | Physical and sexual coercion CTS |  | 0.167 | -0.074 | 0.408 | Intervention | No |
|  |  | Depression | HSCL |  | 0.097 | -0.064 | 0.258 | Intervention |  |
| 16^1^ | Post intervention | Victimisation | Physical PVI current partner |  | 0.303 | -0.048 | 0.655 | Intervention | No |
|  |  |  | Physical PVI ex-partner |  | 0.128 | -0.222 | 0.478 | Intervention |  |
|  |  | Perpetration | Physical PVI current partner |  | 0.183 | -0.167 | 0.533 | Intervention |  |
|  |  |  | Physical PVI ex-partner |  | -0.022 | -0.371 | 0.327 | Control |  |
|  |  | Depression | BDI |  | 0.026 | -0.324 | 0.376 | Intervention |  |
| 29 | Post intervention | Victimisation | Physical and emotional combo of IPS, CTS and 12-item scale to assess injury |  | 0.211 | -0.240 | 0.661 | Intervention | No |
|  |  | Depression | CES-D |  | 0.419 | -0.035 | 0.873 | Intervention |  |
|  | Follow-up | Victimisation | Physical and emotional combo of IPS, CTS and 12-item scale to assess injury |  | -0.033 | -0.482 | 0.416 | Control | No |
|  |  | Depression | CES-D |  | 0.347 | -0.106 | 0.799 | Intervention |  |
| 30 | Post intervention | Victimisation | Physical, emotional and harassment CAS |  | 0.323 | -0.047 | 0.693 | Intervention | No |
|  |  | Depression | EPDS |  | 0.183 | -0.185 | 0.550 | Intervention |  |
|  |  | General MH | SF-36 MH subscale |  | 0.076 | -0.291 | 0.443 | Intervention |  |
| 31 | Post intervention | Victimisation | Psychological CTS |  | 0.468 | 0.082 | 0.854 | Intervention | Yes - Positive |
|  |  |  | Minor physical CTS |  | 0.471 | 0.085 | 0.857 | Intervention |  |
|  |  |  | Severe physical CTS |  | -0.087 | -0.468 | 0.294 | Control |  |
|  |  |  | Sexual CTS |  | 0.223 | -0.159 | 0.605 | Intervention |  |
|  |  | Depression | EPDS |  | 0.749 | 0.256 | 1.242 | Intervention |  |
|  |  | General MH | SF-36 MH subscale |  | -0.541 | -0.929 | -0.153 | Control |  |
| 35 | Post intervention | Victimisation | Physical, psychological, sexual CTS2 |  | -0.134 | -0.669 | 0.400 | Control | No |
|  |  | PTSD | Davidson Trauma Scale |  | 0.325 | -0.213 | 0.862 | Intervention |  |
|  |  |  | LIFE |  | 0.358 | -0.226 | 0.943 | Intervention |  |
|  |  | Depression | LIFE |  | 0.027 | -0.553 | 0.607 | Intervention |  |
|  |  |  | EPDS |  | 0.086 | -0.448 | 0.620 | Intervention |  |
|  | Follow-up | Victimisation | Physical, psychological, sexual CTS2 |  | -0.161 | -0.696 | 0.374 | Control | No |
|  |  | Depression | LIFE |  | 0.027 | -0.553 | 0.607 | Intervention |  |
|  |  |  | EPDS |  | 0.324 | -0.213 | 0.861 | Intervention |  |
|  |  | PTSD | LIFE |  | 0.358 | -0.226 | 0.943 | Intervention |  |
|  |  |  | Davidson Trauma Scale |  | 0.053 | -0.481 | 0.587 | Intervention |  |
| 36 | Post intervention | Victimisation | Psychological CTS-2 |  | 0.632 | 0.189 | 1.076 | Intervention | No |
|  |  |  | Minor physical CTS-2 |  | 0.000 | 0.000 | 0.000 | NEITHER |  |
|  |  |  | Severe physical CTS-2 |  | 0.000 | 0.000 | 0.000 | NEITHER |  |
|  |  |  | Sexual CTS-2 |  | 0.000 | 0.000 | 0.000 | NEITHER |  |
|  |  | General MH | SF-36 subscale |  | 0.308 | -0.126 | 0.746 | Intervention |  |
| 37a | Post intervention | Victimisation | HITS |  | 0.318 | -0.689 | 1.326 | Intervention | No |
|  |  | Perpetration |  |  | 0.085 | -0.821 | 0.991 | Intervention |  |
|  |  | General MH | SSQ |  | 0.503 | -0.290 | 1.296 | Intervention |  |
| 37b | Post intervention | Victimisation | HITS |  | 0.780 | -0.489 | 2.049 | Intervention | No |
|  |  | Perpetration |  |  | 0.780 | -0.489 | 2.049 | Intervention |  |
|  |  | General MH | SSQ |  | -0.016 | -0.653 | 0.622 | Control |  |
| ^1^RCT includes three arms; effect sizes calculated based on comparison between intervention arm and standard control arm. BDI = Beck Depression Inventory; CAS = Composite Abuse Scale; CES-D = Center for Epidemiologic Studies Depression Scale; CTS = Conflict Tactics Scale; DVA = Domestic violence and abuse; EPDS = Edinburgh Postnatal Depression Scale; HITS = Hurt, Insult, Threaten, Scream; HSCL = Hopkins Symptom Checklist; LIFE = Longitudinal Interval Follow-up Examination; MH = Mental ill-health; NR = Not reported; NS = No sig difference between intervention and control; PVI = Partner Violence Index; SF- = Short Form-; SSQ = Shona Symptom Questionnaire. | | | | | | | | | |

| **Study outcomes for studies measuring DVA and SU** | | | | | | | | | |
| --- | --- | --- | --- | --- | --- | --- | --- | --- | --- |
| **Study number** | **Timepoint** | **Overarching construct** | **Measure** | **Authors’ reported results** | **Cohen’s d** | **95% CI – lower** | **95% CI – upper** | **Direction of effect** | **Combined impacts** |
| 10 | Post intervention | Perpetration | Physical, psychological, sexual coercion and injury CTS2 short form |  | -0.027 | -0.155 | 0.103 | Control | No |
|  |  | Victimisation | Physical, psychological, sexual coercion and injury CTS2 short form |  | -0.029 | -0.157 | 0.096 | Control |  |
|  |  | Alcohol use | YRBSS | NR | N/A | N/A | N/A | N/A |  |
|  |  | Drug use | YRBSS | NR | N/A | N/A | N/A | N/A |  |
|  |  | Marijuana use | YRBSS |  | 0.171 | 0.020 | 0.323 | Intervention |  |
| 12^1^ | Post intervention | Victimisation/perpetration | TLFB-SV male to female |  | -0.029 | -0.905 | 0.848 | Control | No |
|  |  |  | TLFB-SV female to male |  | 0.015 | -0.861 | 0.892 | Intervention |  |
|  |  | Alcohol | TLFB |  | -0.130 | -1.007 | 0.748 | Control |  |
|  | Follow-up | Victimisation/perpetration | TLFB-SV male to female |  | 0.015 | -0.862 | 0.892 | Intervention | No |
|  |  |  | TLFB-SV female to male |  | 0.014 | -0.862 | 0.891 | Intervention |  |
|  |  | Alcohol | TLFB |  | 0.040 | -0.836 | 0.917 | Intervention |  |
| 13 | Post intervention | Victimisation | Physical CTS2 |  | 1.125 | 0.802 | 1.448 | Intervention | No |
|  |  | Alcohol | Own questions |  | 0.351 | -0.157 | 0.859 | Intervention |  |
| 25 | Post intervention | Perpetration | Physical CTS2 |  | -0.046 | -0.970 | 0.879 | Control | No |
|  |  | Victimisation | Physical CTS2 |  | 0.282 | -0.646 | 1.211 | Intervention |  |
|  |  | Substance use | TLFB | NS differences between groups. | N/A | N/A | N/A | NR |  |
| ^1^RCT includes three arms; effect sizes calculated based on comparison between intervention arm and behavioural couples therapy arm. CTS2 = Conflict Tactics Scale; DVA = Domestic violence and abuse; NR = Not reported; NS = No sig difference between intervention and control; SU = Substance misuse; TLFB = Timeline Follow Back Interview; TLFB-SV = Timeline Follow Back Interview-Spousal Violence; YRBSS = Youth Risk Behaviour Surveillance System. | | | | | | | | | |

| **Study outcomes for studies measuring MH and SU** | | | | | | | | | |
| --- | --- | --- | --- | --- | --- | --- | --- | --- | --- |
| **Study number** | **Timepoint** | **Overarching construct** | **Measure** | **Authors’ reported results** | **Cohen’s d** | **95% CI – lower** | **95% CI – upper** | **Direction of effect** | **Combined impacts** |
| 1 | Post intervention | General MH | SF-36 MH subscale |  | -0.027 | -0.239 | 0.185 | Control | No |
|  |  | Alcohol* | Self-report lifestyle questionnaire |  | -0.111 | -0.323 | 0.101 | Control |  |
|  |  | Drug* | Self-report lifestyle questionnaire |  | -0.023 | -0.362 | 0.316 | Control |  |
| 6 | Post intervention | Depression | EPDS |  | 0.223 | -0.052 | 0.498 | Intervention | No |
|  |  | Alcohol | TLFB mean n of drinks in previous 28 days |  | 0.354 | 0.096 | 0.612 | Intervention |  |
|  |  |  | TLFB mean n of drinking days in previous 28 days |  | 0.144 | -0.112 | 0.400 | Intervention |  |
|  |  |  | TLFB mean n of heavy drinking days in previous 28 days |  | 0.337 | 0.079 | 0.595 | Intervention |  |
| 7 | Post intervention | Depression | BDI |  | N/A | N/A | N/A | NR | No |
|  |  | Depression | SCL-90 R depression subscale |  | N/A | N/A | N/A | NR |  |
|  |  | Anxiety | SCL-90 R anxiety subscale |  | N/A | N/A | N/A | NR |  |
|  |  | Obsessive compulsive | SCL-90 R OC subscale |  | N/A | N/A | N/A | NR |  |
|  |  | General MH (father) | SCL-90 R GSI |  | 0.139 | -0.399 | 0.676 | Intervention |  |
|  |  | General MH (mother) | SCL-90 R GSI |  | 0.404 | -0.138 | 0.946 | Intervention |  |
|  |  | Alcohol | ADD |  | -0.023 | -0.560 | 0.514 | Control |  |
| 11 | Post intervention | Depression | BDI-II |  | 0.663 | 0.093 | 1.233 | Intervention | Yes - Mixed |
|  |  | Heroin | ASI/UT |  | 0.112 | -0.081 | 0.670 | Intervention |  |
|  |  |  | UT |  | 0.217 | -0.343 | 0.776 | Intervention |  |
|  |  |  | ASI |  | -1.782 | -2.422 | -1.142 | Control |  |
|  |  | Cocaine | ASI/UT |  | 0.407 | -0.155 | 0.970 | Intervention |  |
|  |  |  | UT |  | 0.493 | -0.072 | 1.058 | Intervention |  |
|  |  |  | ASI |  | 0.388 | -0.175 | 0.950 | Intervention |  |
|  |  | Alcohol | ASI any use |  | -1.837 | -2.482 | -1.192 | Control |  |
|  |  |  | ASI intoxication |  | -1.267 | -1.868 | -0.667 | Control |  |
|  |  |  | ASI composite |  | -1.439 | -2.052 | -0.826 | Control |  |
|  |  | Drug | ASI composite |  | -1.644 | -2.273 | -1.016 | Control |  |
|  |  |  | RAB |  | -0.024 | -0.582 | 0.534 | Control |  |
| 14 | Post intervention | Depression | BDI |  | -0.035 | -0.384 | 0.313 | Control | No |
|  |  | Opiate | UT |  | -0.197 | -0.546 | 0.152 | Control |  |
|  |  | Cocaine | UT |  | 0.540 | 0.185 | 0.894 | Intervention |  |
|  | Follow-up | Depression | BDI |  | -0.378 | -0.730 | -0.027 | Control | No |
|  |  | Opiate | UT |  | -0.098 | -0.447 | 0.250 | Control |  |
|  |  | Cocaine | UT |  | -0.108 | -0.457 | 0.241 | Control |  |
| 15a | Post intervention | Depression | CES-D |  | 0.340 | -0.243 | 0.922 | Intervention | No |
|  |  | Alcohol | Own questions |  | 0.490 | -0.097 | 1.078 | Intervention |  |
| 15b | Post intervention | Depression | CES-D |  | -0.340 | -0.922 | 0.243 | Control | No |
|  |  | Alcohol | Own questions |  | -0.490 | -1.078 | 0.097 | Control |  |
| 20 | Follow-up | General MH | BSI |  | 0.167 | -0.057 | 0.391 | Intervention | No |
|  |  | Substance use | Self-report current use |  | 0.499 | 0.014 | 0.884 | Intervention |  |
|  |  |  | Self-report relapse |  | 0.075 | -0.132 | 0.283 | Intervention |  |
| 21 | Post intervention | Depression^+^ | BSI |  | 0.000 | -0.213 | 0.213 | NEITHER | No |
|  |  | Anxiety^+^ | BSI |  | 0.000 | -0.213 | 0.213 | NEITHER |  |
|  |  | General MH | BSI |  | 0.062 | -0.151 | 0.275 | Intervention |  |
|  |  | Alcohol^+^ | Self-report | NR for whole sample. | N/A | N/A | N/A | NR |  |
|  |  | Marijuana^+^ | Self-report |  | 0.000 | -0.213 | 0.213 | NEITHER |  |
|  |  | Hard drugs^+^ | Self-report |  | 0.000 | -0.213 | 0.213 | NEITHER |  |
|  | Follow-up | Depression^+^ | BSI |  | 0.000 | -0.213 | 0.213 | NEITHER | No |
|  |  | Anxiety^+^ | BSI |  | 0.000 | -0.213 | 0.213 | NEITHER |  |
|  |  | General MH | BSI |  | 0.000 | -0.213 | 0.213 | NEITHER |  |
|  |  | Alcohol | Self-report | NR for whole sample. | N/A | N/A | N/A | NR |  |
|  |  | Marijuana^+^ | Self-report |  | 0.000 | -0.213 | 0.213 | NEITHER |  |
|  |  | Hard drugs^+^ | Self-report |  | 0.000 | -0.213 | 0.213 | NEITHER |  |
| 24 | Post intervention | Depression | BDI-II |  | -0.306 | -0.638 | 0.026 | Control | No |
|  |  | Alcohol | Form-90 |  | 0.306 | -0.026 | 0.638 | Intervention |  |
|  |  | Marijuana | Form-90 |  | 0.240 | -0.092 | 0.571 | Intervention |  |
|  |  | Hard drugs | Form-90 |  | -0.108 | -0.439 | 0.222 | Control |  |
|  | Follow-up | Depression | BDI-II |  | -0.065 | -0.397 | 0.268 | Control | No |
|  |  | Alcohol | Form-90 |  | 0.304 | -0.030 | 0.638 | Intervention |  |
|  |  | Marijuana | Form-90 |  | 0.129 | -0.203 | 0.462 | Intervention |  |
|  |  | Hard drugs | Form-90 |  | 0.000 | -0.332 | 0.332 | NEITHER |  |
| 27 | Post intervention | Depression | BDI |  | 0.335 | -0.241 | 0.911 | Intervention | No |
|  |  | General MH | BSI |  | -0.085 | -0.657 | 0.487 | Control |  |
|  |  | Drug | UT |  | 0.056 | -0.517 | 0.628 | Intervention |  |
|  | Follow-up | Depression | BDI |  | -0.335 | -0.911 | 0.241 | Control | No |
|  |  | General MH | BSI |  | -0.018 | -0.590 | 0.554 | Control |  |
|  |  | Drug | UT |  | -0.045 | -0.617 | 0.527 | Control |  |
| 28 | Post intervention | Depression | BDI | NR. | N/A | N/A | N/A | NR | Yes - Negative |
|  |  | General MH | BSI |  | -1.506 | -1.029 | -1.984 | Control |  |
|  |  | Heroin | TLFB |  | -0.667 | -1.100 | -0.234 | Control |  |
|  |  | Opioid | TLFB |  | 0.000 | 0.000 | 0.000 | NEITHER |  |
|  |  | Cocaine | TLFB |  | -0.333 | -0.758 | 0.091 | Control |  |
|  | Follow-up | Depression | BDI | NS change over time. | N/A | N/A | N/A | N/A | Yes - Mixed |
|  |  | General MH | BSI |  | -1.259 | -1.721 | -0.798 | Control |  |
|  |  | Heroin | TLFB |  | 1.000 | 0.553 | 1.447 | Intervention |  |
|  |  | Opioid | TLFB |  | 0.000 | 0.000 | 0.000 | NEITHER |  |
|  |  | Cocaine | TLFB |  | 1.029 | 0.581 | 1.478 | Intervention |  |
| 33 | Post intervention | General MH | BSI | NS change over time. | N/A | N/A | N/A | N/A | No |
|  |  | Cocaine | ASI | Sig difference between groups in terms of change in self-reported cocaine use over time (favouring intervention). | N/A | N/A | N/A | N/A |  |
|  |  |  | UT | NS urinalysis of cocaine over time. | N/A | N/A | N/A | N/A |  |
|  | Follow-up | General MH | BSI | NS change over time (baseline to follow-up). | N/A | N/A | N/A | N/A | No |
|  |  | Cocaine (self-report) | ASI | Sig difference between groups in terms of change over time (baseline to follow-up). Favours intervention. | N/A | N/A | N/A | N/A |  |
|  |  | Cocaine (urinalysis) | UT | NS (not sure whether this is change across time or at follow-up data collection point). | N/A | N/A | N/A | N/A |  |
| 34 | Post intervention | Depression | CES-D |  | 0.379 | -0.042 | 0.801 | Intervention | No |
|  |  | Alcohol | Items from SAMHSA | NS change over time. | N/A | N/A | N/A | N/A |  |
|  |  | Illegal Substances | Items from SAMHSA | NS change over time. | N/A | N/A | N/A | N/A |  |
|  | Follow-up | Depression | CES-D | NS change over time (T2 to follow-up i.e., T3). | N/A | N/A | N/A | N/A | No |
|  |  | Alcohol | Items from SAMHSA | NS change over time (T2 to follow-up i.e., T3). | N/A | N/A | N/A | N/A |  |
|  |  | Illegal substances | Items from SAMHSA | NS change over time (T2 to follow-up i.e., T3). | N/A | N/A | N/A | N/A |  |
| ^+^Where results reported as NS, and no other information provided, SMD imputed as 0 and CIs calculated using sample size (Borenstein et al., 2009). ADD = Alcohol Dependency Data; ASI = Addiction Severity Index; BDI = Beck Depression Inventory; BSI = Brief Symptom Inventory; CES-D = Center for Epidemiologic Studies Depression Scale; EPDS = Edinburgh Postnatal Depression Scale; MH = Mental ill-health; NR = Not reported; NS = No sig difference between intervention and control; RAB = Risk Assessment Battery; SAMHSA = Substance Abuse and Mental Health Services Administration; SF- = Short Form-; SCL-90-R = Symptom Checklist 90 Revised; SU = Substance misuse; TLFB = Timeline Follow Back Interview; UT = Urine toxicology. | | | | | | | | | |

| **Study outcomes for studies measuring DVA, MH, and SU** | | | | | | | | | |
| --- | --- | --- | --- | --- | --- | --- | --- | --- | --- |
| **Study number** | **Timepoint** | **Overarching construct** | **Measure** | **Authors’ reported results** | **Cohen’s d** | **95% CI – lower** | **95% CI – upper** | **Direction of effect** | **Combined impacts** |
| 2 | Post intervention | Victimisation/perpetration | Psychological CTS2 |  | 0.07 | -1.221 | 1.361 | Intervention | No |
|  |  |  | Physical CTS2 |  | -0.06 | -0.666 | 0.546 | Control |  |
|  |  |  | Injury CTS2 |  | 0.15 | -0.432 | 0.732 | Intervention |  |
|  |  | Depression | CES-D |  | 0.14 | -0.179 | 0.316 | Intervention |  |
|  |  | General MH | MHI-5 |  | 0.14 | -0.179 | 0.316 | Intervention |  |
|  |  | Drug | Self-report |  | -0.106 | -0.452 | 0.241 | Control |  |
|  |  | Alcohol | CAGE |  | 0.350 | -0.020 | 0.721 | Intervention |  |
|  |  | Drug and Alcohol | Self-report/CAGE |  | 0.130 | -0.168 | 0.428 | Intervention |  |
| 3 | Post intervention | Victimisation/perpetration | Psychological CTS2 |  | -0.092 | -0.284 | 0.100 | Control | No |
|  |  |  | Physical CTS2 |  | 0.027 | -0.179 | 0.232 | Intervention |  |
|  |  |  | Injury CTS |  | 0.038 | -0.208 | 0.285 | Intervention |  |
|  |  | Depression | CES-D |  | 0.000 | -0.256 | 0.256 | NEITHER |  |
|  |  | General MH | MHI-5 |  | 0.078 | -0.125 | 0.282 | Intervention |  |
|  |  | Drug | Self-report |  | -0.123 | -0.440 | 0.194 | Control |  |
|  |  | Alcohol | CAGE |  | 0.215 | -0.122 | 0.551 | Intervention |  |
|  | Follow-up | Victimisation | Psychological, physical, and injury CTS2 |  | 0.000 | -0.184 | 0.184 | NEITHER | No |
|  |  | Perpetration | Psychological, physical, and injury CTS2 |  | -0.041 | -0.225 | 0.143 | Control |  |
|  |  | Depression | CES-D |  | 0.123 | -0.216 | 0.462 | Intervention |  |
|  |  | General MH | MHI-5 |  | 0.000 | -0.243 | 0.243 | NEITHER |  |
|  |  | Alcohol | CAGE |  | 0.194 | -0.154 | 0.541 | Intervention |  |
|  |  | Drug | ASI |  | 0.123 | -0.216 | 0.462 | Intervention |  |
| 5 | Post intervention | Victimisation | Physical CTS2 |  | -0.105 | -0.511 | 0.301 | Control | No |
|  |  | Depression | Items from CIDI |  | -0.040 | -0.336 | 0.256 | Control |  |
|  |  | Alcohol | Items from CIDI |  | -0.234 | -0.579 | 0.112 | Control |  |
|  |  | Drug | Items from CIDI |  | 0.098 | -0.389 | 0.585 | Intervention |  |
|  | Follow-up | Victimisation | Psychological and physical CTS2 |  | -0.040 | -0.245 | 0.164 | Control | No |
|  |  | Perpetration | Psychological and physical CTS2 |  | -0.020 | -0.224 | 0.184 | Control |  |
|  |  | Depression | Items from CIDI |  | -0.053 | -0.257 | 0.152 | Control |  |
|  |  | Alcohol | Items from CIDI |  | -0.116 | -0.538 | 0.306 | Control |  |
|  |  | Drug | Items from CIDI |  | -0.185 | -0.461 | 0.091 | Control |  |
| 8 | Post intervention | Victimisation | Emotional WEB |  | 0.012 | -0.494 | 0.519 | Intervention | No |
|  |  | Depression | BDI-II |  | 0.022 | -0.484 | 0.529 | Intervention |  |
|  |  | General MH | SF-36 MH |  | -0.052 | -0.558 | 0.454 | Control |  |
|  |  | Alcohol | Form90/UT |  | 0.558 | 0.043 | 1.074 | Intervention |  |
|  |  | Drug | Form90/UT |  | -0.056 | -0.562 | 0.451 | Control |  |
|  | Follow-up | Victimisation | Emotional WEB |  | 0.027 | -0.479 | 0.533 | Intervention | No |
|  |  | Depression | BDI-II |  | 0.013 | -0.494 | 0.519 | Intervention |  |
|  |  | General MH | SF-36 MH |  | 0.005 | -0.502 | 0.502 | Intervention |  |
|  |  | Alcohol | Form90/UT |  | -0.177 | -0.708 | 0.355 | Control |  |
|  |  | Drug | Form90/UT |  | 0.145 | -0.386 | 0.677 | Intervention |  |
| 9 | Post intervention | Victimisation | CAS |  | -0.249 | -0.563 | 0.066 | Control | No |
|  |  | PTSD | SPAN |  | 0.045 | -0.234 | 0.324 | Intervention |  |
|  |  | Depression | PRIME-MD (PHQ-9) |  | 0.083 | -0.227 | 0.393 | Intervention |  |
|  |  | General MH | SF-12 MH |  | -0.112 | -0.290 | 0.065 | Control |  |
|  |  | Alcohol | TWEAK |  | -0.131 | -0.420 | 0.159 | Control |  |
|  |  | Drug | DAST |  | -0.272 | -0.659 | 0.115 | Control |  |
| 17a^1^ | Follow-up | Victimisation | Physical CTS2 (past 6m) |  | -0.028 | -0.329 | 0.273 | Control | No |
|  |  | Victimisation | Physical CTS2 (past 12m) |  | 0.056 | -0.192 | 0.305 | Intervention |  |
|  |  | General MH | NR |  | 0.211 | 0.021 | 0.400 | Intervention |  |
|  |  | Alcohol | Own measure |  | 0.195 | -0.402 | 0.791 | Intervention |  |
|  |  | Marijuana | Own measure |  | 0.033 | -0.328 | 0.395 | Intervention |  |
| 17b^1^ | Follow-up | Victimisation | Physical CTS2 (past 6m) |  | 0.415 | 0.049 | 0.782 | Intervention | No |
|  |  | Victimisation | Physical CTS2 (past 12m) |  | 0.278 | 0.009 | 0.547 | Intervention |  |
|  |  | General MH | NR |  | 0.068 | -0.123 | 0.258 | Intervention |  |
|  |  | Alcohol | Own measure |  | -0.058 | -0.594 | 0.479 | Control |  |
|  |  | Marijuana | Own measure |  | 0.083 | -0.289 | 0.455 | Intervention |  |
| 18^2^ | Follow-up | Victimisation | Physical CTS2 |  | -0.014 | -0.123 | 0.096 | Control | No |
|  |  | Depression | BDI |  | -0.059 | -0.401 | 0.283 | Control |  |
|  |  | Anxiety | BAI |  | 0.040 | -0.287 | 0.367 | Intervention |  |
|  |  | Substance use | Drug use screening inventory |  | 0.075 | -0.142 | 0.292 | Intervention |  |
| 19^3^ | Post intervention | Victimisation | Physical and injury CTS2 |  | -0.152 | -0.302 | 0.294 | Control | No |
|  |  | Perpetration | Physical and injury CTS2 |  | 0.126 | -0.263 | 0.232 | Intervention |  |
|  |  | Depression | EPDS |  | 0.132 | -0.101 | 0.365 | Intervention |  |
|  |  | Alcohol | ASSIST |  | 0.066 | -0.218 | 0.040 | Intervention |  |
|  |  | Drug | ASSIST |  | 0.120 | -0.413 | 0.057 | Intervention |  |
|  | Follow-up | Victimisation^+^ | Physical and injury CTS2 |  | 0.000 | -0.233 | 0.233 | NEITHER | No |
|  |  | Perpetration^+^ | Physical and injury CTS2 |  | 0.000 | -0.233 | 0.233 | NEITHER |  |
|  |  | Depression | EPDS |  | -0.080 | -0.313 | 0.154 | Control |  |
|  |  | Alcohol^+^ | ASSIST |  | 0.000 | -0.233 | 0.233 | NEITHER |  |
|  |  | Drugs^+^ | ASSIST |  | 0.000 | -0.233 | 0.233 | NEITHER |  |
| 22 | Post intervention | Victimisation | Physical items adapted from Jewkes et al. |  | -0.080 | -0.211 | 0.050 | Control | No |
|  |  | Depression | EPDS and GHQ-12 |  | -0.060 | -0.191 | 0.070 | Control |  |
|  |  | Alcohol | AUDIT-C latent variable |  | 0.060 | -0.070 | 0.191 | Intervention |  |
|  | Follow-up | Victimisation | Physical items adapted from Jewkes et al. |  | -0.040 | -0.171 | 0.091 | Control | No |
|  |  | General MH | EPDS and GHQ-12 |  | 0.141 | 0.010 | 0.271 | Intervention |  |
|  |  | Alcohol | AUDIT-C latent variable |  | 0.040 | -0.091 | 0.171 | Intervention |  |
| 23 | Post intervention | Victimisation | All CTS2 | NR. Not powered. | N/A | N/A | N/A | NR | No |
|  |  | Depression | BDI-II |  | 0.038 | -0.346 | 0.422 | Intervention |  |
|  |  | Alcohol | DIS alcohol module | NR. Not powered. | N/A | N/A | N/A | NR |  |
|  |  | Drug | DIS drug module | NR. Not powered. | N/A | N/A | N/A | NR |  |
|  | Follow-up | Victimisation | All CTS2 | NR. Not powered. | N/A | N/A | N/A | NR | No |
|  |  | Depression | BDI-II |  | -0.319 | -0.705 | 0.068 | Control |  |
|  |  | Alcohol | DIS alcohol module | NR. Not powered. | N/A | N/A | N/A | NR |  |
|  |  | Drugs | DIS drug module | NR. Not powered. | N/A | N/A | N/A | NR |  |
| 26 | Post intervention | Perpetration | Psychological, verbal and physical TLFB-SV |  | -0.128 | -0.627 | 0.371 | Control | No |
|  |  | General MH | BSI (Global severity) |  | -0.048 | -0.547 | 0.451 | Control |  |
|  |  | Substance use | TLFB-SU |  | 0.320 | -0.184 | 0.824 | Intervention |  |
|  | Follow-up | Perpetration | Psychological, verbal and physical TLFB-SV |  | -0.192 | -0.692 | 0.303 | Control | No |
|  |  |  | Psychological TLFB-SV |  | 0.170 | -0.330 | 0.670 | Intervention |  |
|  |  |  | Physical TLFB-SV |  | -0.620 | -0.671 | -0.109 | Control |  |
|  |  | Victimisation | Psychological TLFB-SV |  | 0.160 | -0.340 | 0.660 | Intervention |  |
|  |  |  | Physical TLFB-SV |  | -0.810 | -1.329 | -0.291 | Control |  |
|  |  | General MH | BSI (Global severity) |  | -0.072 | -0.571 | 0.427 | Control |  |
|  |  | Substance use | TLFB-SU |  | 0.480 | -0.026 | 0.986 | Intervention |  |
| 32 | Post intervention | Victimisation | Physical, emotional, harassment CAS |  | -0.781 | -2.078 | 0.516 | Control | No |
|  |  | Depression | EPDS |  | 0.495 | -0.068 | 1.059 | Intervention |  |
|  |  |  | PHQ-9 |  | 0.285 | -0.366 | 0.936 | Intervention |  |
|  |  | Anxiety | GAD-7 |  | 0.435 | -0.231 | 1.101 | Intervention |  |
|  |  | Alcohol | AUDIT-C |  | -0.107 | -0.749 | 0.535 | Control |  |
|  | Follow-up | Victimisation | Physical, emotional, harassment CAS | NR. Too few numbers. | N/A | N/A | N/A | N/A | No |
|  |  | Depression | EPDS |  | 0.462 | -0.105 | 1.030 | Intervention |  |
|  |  |  | PHQ-9 |  | -0.027 | -0.862 | 0.808 | Control |  |
|  |  | Anxiety | GAD-7 |  | 0.662 | -0.035 | 1.359 | Intervention |  |
|  |  | Alcohol | AUDIT-C |  | -0.172 | -0.834 | 0.490 | Control |  |
| ^+^ Where results reported as NS, and no other information provided, SMD imputed as 0 and CIs calculated using imputed SMD and sample size (Borenstein et al., 2009). ^1^RCT includes three arms; effect sizes calculated based on comparison between intervention arm and standard control arm. ^2^RCT includes four arms; effect sizes calculated based on comparison between intervention arm and standard control arm that were followed up at postpartum. ^3^RCT includes three arms; effect sizes calculated based on comparison between intervention arm and active control arm. ASI = Addiction Severity Index; ASSIST = Alcohol Smoking and Substance Involvement Screening Test; AUDIT-C = Alcohol use disorders identification test for consumption; BAI = Beck Anxiety Inventory; BDI = Beck Depression Inventory; BSI = Brief Symptom Inventory; CAS = Composite Abuse Scale; CES-D = Center for Epidemiologic Studies Depression Scale; CIDI = Composite International Diagnostic Interview; CTS = Conflict Tactics Scale; DAST = Drug Abuse Screening Test; DIS = Diagnostic Inventory Schedule; DVA = Domestic violence and abuse; EPDS = Edinburgh Postnatal Depression Scale; GAD-7 = Generalised Anxiety Disorder-7; GHQ = General Health Questionnaire; GSI = Global Severity Index; MH = Mental ill-health; MHI-5 = Mental Health Index-5; NR = Not reported; NS = No sig difference between intervention and control; OCD = Obsessive Compulsive Disorder; PHQ-9 = Patient Health Questionnaire-9; PTSD = Post traumatic stress disorder; SF- = Short Form-; SU = Substance misuse; TLFB = Timeline Follow Back Interview; TLFB-SV = Timeline Follow Back Interview-Spousal Violence; UT = Urine toxicology; WEB = Women’s Experience of Battering Scale. | | | | | | | | | |

**Harvest plots illustrating direction of effects for DVA, MH, and SU outcomes at post-intervention and follow-up.**

**Fig 1. Direction of effects for DVA, MH, and SU outcomes at post-intervention**

**Bars** represent studies; **Height of the bar** represents whether any of the SMDs 95% confidence intervals are positive, cross 0, or are negative; **Numbers above bars** represent number of outcome measures the categorisation is based on; **Number in bars** represent the study number; **Colour** represents the combination of outcomes the study measures (see key).

**NB.** + some SMDs = 0.00 favouring neither intervention nor control. **15a** = goal-orientated therapy as ‘intervention’ group; **15b** = emotion-focused therapy as ‘intervention’ group; **37a** = parenting intervention compared with usual care control; **37b** = parenting intervention plus violence curriculum compared with usual care control.

**7** also measured depression, anxiety, and OCD but values were not reported. **10** also measured alcohol and drug use but do not report values. **21** also measure alcohol use but values were not reported for the whole sample. **23** measured victimisation, alcohol and drug use but do not report results as underpowered to detect a difference. **25** also measured substance use; authors reported no sig differences between groups in terms of change over time (baseline to follow-up). **28** authors also measure depression but do not report. **33** measured general MH and cocaine use. Authors report no sig differences between intervention and control in terms of change over time in general MH or urinalysis of cocaine use (baseline to follow-up). Authors report a sig difference between groups in terms of change over time in addiction severity index cocaine use (baseline to follow-up; favouring intervention). **34** measured alcohol and illegal substance use. Authors report no sig differences between groups in terms of change over time (post-intervention to follow-up).

**
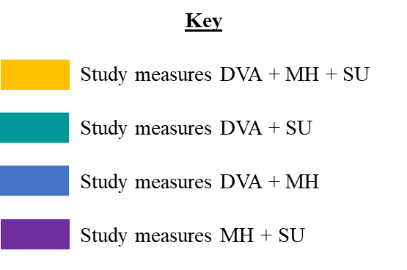
**


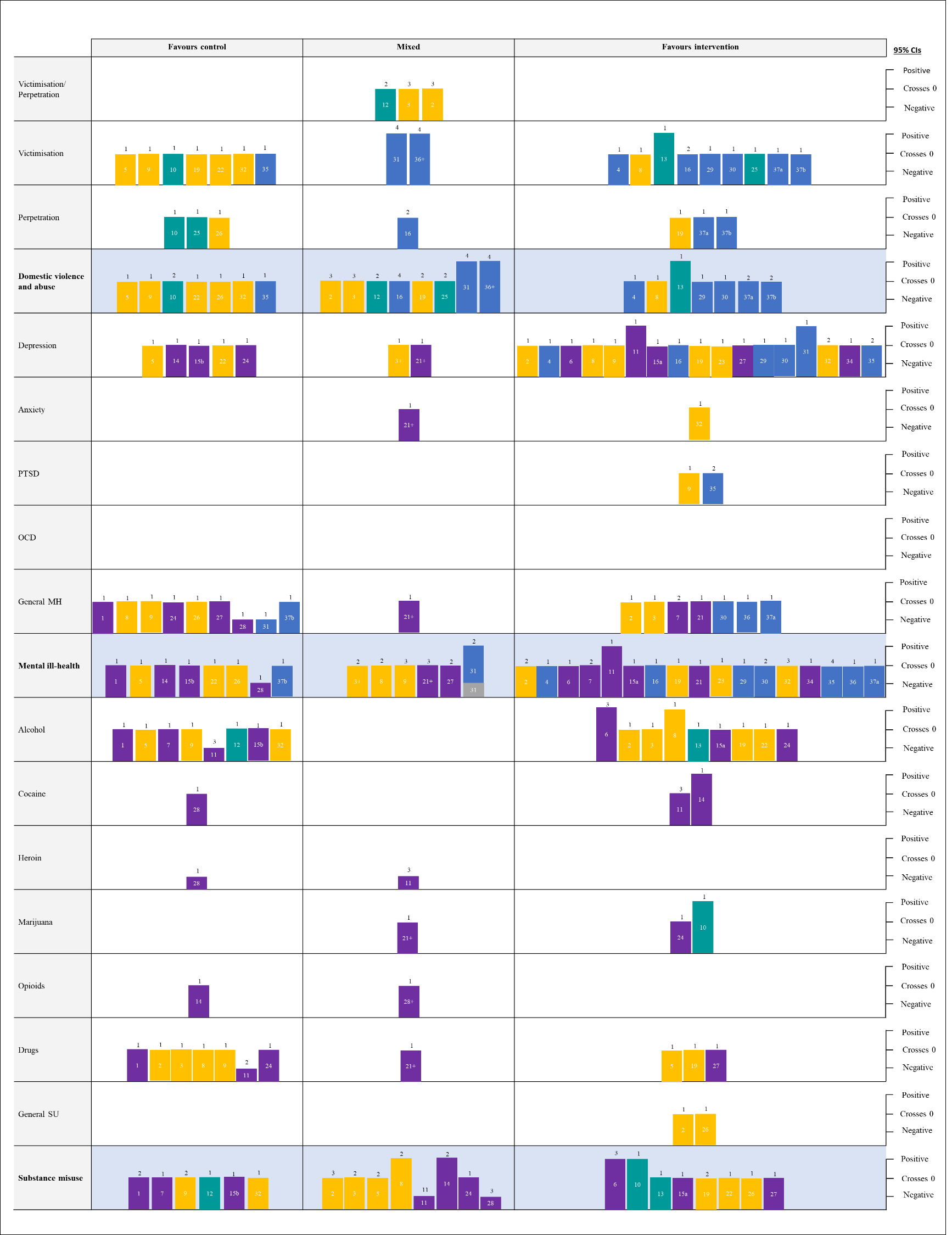


**Fig 2. Direction of effects for DVA, MH, and SU outcomes at follow-up**

**Bars** represent studies; **Height of the bar** represents whether any of the SMDs 95% confidence intervals are positive, cross 0, or are negative; **Numbers above bars** represent number of outcome measures the categorisation is based on; **Number in bars** represent the study number; **Colour** represents the combination of outcomes the study measures (see key).

**NB.** + some SMDs = 0.00 favouring neither intervention nor control. **17a** = paraprofessional delivered home visiting intervention compared with minimal care control; **17b** = nurse delivered home visiting intervention compared with minimal care control.

**21** also measure alcohol use but values were not reported for the whole sample. **23** also measured victimisation, alcohol and drug use but do not report results as underpowered to detect a difference. **28** authors also measure depression but do not report. **32** also measured victimisation but do not report values as too few numbers. **33** measured general MH and cocaine use. Authors report no sig differences between intervention and control in terms of change over time in general MH or urinalysis of cocaine use (baseline to follow-up). Authors report a sig difference between groups in terms of change over time in addiction severity index cocaine use (baseline to follow-up; favouring intervention). **34** measured alcohol and illegal substance use. Authors report no sig differences between groups in terms of change over time (post-intervention to follow-up).

**
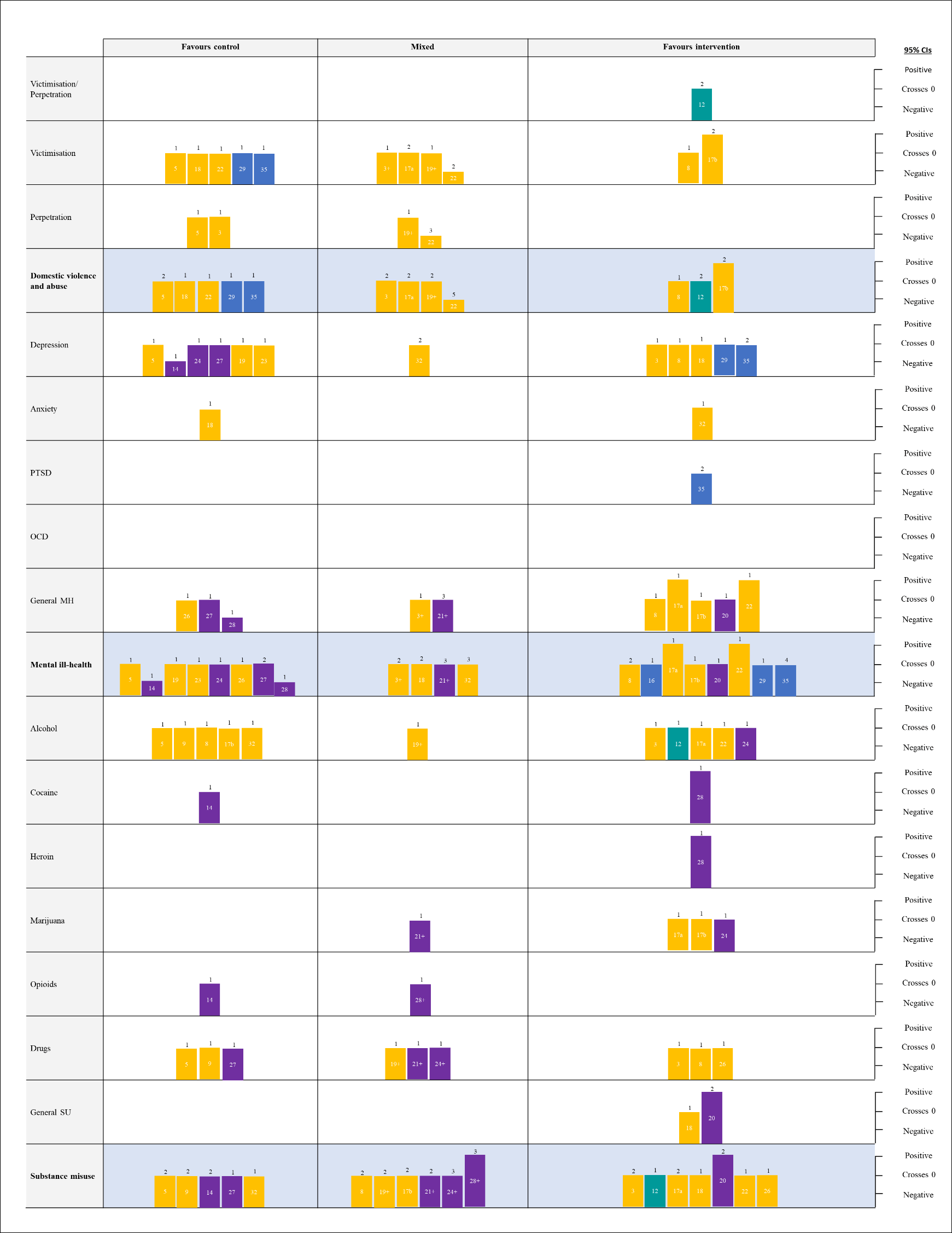

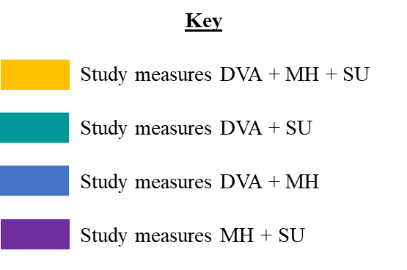
**

**References**

1. Transforming the response to domestic abuse: consultation response and draft bill, (2019).

2. National Institute for Health and Care Excellence. Common mental health problems: identification and pathways to care (CG123). 2011 Contract No.: CG123.

3. Kroll B, Taylor A. Parental substance misuse and child welfare. London: Jessica Kingsley Publishers; 2003.

4. World Health Organisation (WHO). Lexicon of alcohol and drug terms. Barbor T, Campbell R, Room R, Saunders J, editors. Geneva: WHO; 1994.

5. Advisory Council on the Misuse of Drugs (ACMD). Vulnerability and drug use report. Advisory Council on the Misuse of Drugs, 2018.

6. Felitti VJMD, Facp, Anda RFMD, Ms, Nordenberg DMD, Williamson DFMS, et al. Relationship of Childhood Abuse and Household Dysfunction to Many of the Leading Causes of Death in Adults: The Adverse Childhood Experiences (ACE) Study. American Journal of Preventive Medicine. 1998;14(4):245-58. doi: 10.1016/S0749-3797(98)00017-8.

7. Anda RF, Croft JB, Felitti VJ, Nordenberg D, Giles WH, Williamson DF, et al. Adverse Childhood Experiences and Smoking During Adolescence and Adulthood. JAMA. 1999;282(17):1652-8. doi: 10.1001/jama.282.17.1652.

8. Dong M, Anda RF, Felitti VJ, Dube SR, Williamson DF, Thompson TJ, et al. The interrelatedness of multiple forms of childhood abuse, neglect, and household dysfunction. Child Abuse & Neglect. 2004;28(7):771-84. doi: 10.1016/j.chiabu.2004.01.008.

9. Bernard DL, Calhoun CD, Banks DE, Halliday CA, Hughes-Halbert C, Danielson CK. Making the “C-ACE” for a Culturally-Informed Adverse Childhood Experiences Framework to Understand the Pervasive Mental Health Impact of Racism on Black Youth. Journal of Child & Adolescent Trauma. 2021;14(2):233-47. doi: 10.1007/s40653-020-00319-9.

10. Wade R, Shea JA, Rubin D, Wood J. Adverse Childhood Experiences of Low-Income Urban Youth. Pediatrics. 2014;134(1):e13. doi: 10.1542/peds.2013-2475.

11. Bellis MA, Hughes K, Ford K, Hardcastle KA, Sharp CA, Wood S, et al. Adverse childhood experiences and sources of childhood resilience: a retrospective study of their combined relationships with child health and educational attendance. BMC Public Health. 2018;18(1):792. Epub 2018/06/27. doi: 10.1186/s12889-018-5699-8. PubMed PMID: 29940920; PubMed Central PMCID: PMCPMC6020215.

12. Hughes K, Bellis MA, Hardcastle KA, Sethi D, Butchart A, Mikton C, et al. The effect of multiple adverse childhood experiences on health: a systematic review and meta-analysis. The Lancet Public Health. 2017;2(8):e356-e66. doi: 10.1016/S2468-2667(17)30118-4.

13. Merrick MT, Ford DC, Ports KA, Guinn AS, Chen J, Klevens J, et al. Vital Signs: Estimated Proportion of Adult Health Problems Attributable to Adverse Childhood Experiences and Implications for Prevention — 25 States, 2015–2017. Morbidity and Mortality Weekly Report. 2019;68.

14. Bellis MA, Lowey H, Leckenby N, Hughes K, Harrison D. Adverse childhood experiences: retrospective study to determine their impact on adult health behaviours and health outcomes in a UK population. Journal of Public Health (Oxford). 2014;36(1):81-91. Epub 2013/04/17. doi: 10.1093/pubmed/fdt038. PubMed PMID: 23587573.

15. Skar A-MS, Sherr L, Macedo A, Tetzchner Sv, Fostervold KI. Evaluation of parenting interventions to prevent violence against children in Colombia: a randomized controlled trial. Journal of Interpersonal Violence. 2017;36(1-2):NP1098-NP126. doi: 10.1177/0886260517736881.

16. Olds DL, Kitzman H, Hanks C, Cole R, Anson E, Sidora-Arcoleo K, et al. Effects of nurse home visiting on maternal and child functioning: age-9 follow-up of a randomized trial. Pediatrics. 2007;120(4):e832-45.

17. Fergusson DM, Grant H, Horwood LJ, Ridder EM. Randomized trial of the Early Start program of home visitation: parent and family outcomes. Pediatrics. 2006;117(3):781-6.

18. LeCroy CW, Krysik J. Randomized trial of the healthy families Arizona home visiting program. Children and Youth Services Review. 2011;33(10):1761-6. doi: <http://dx.doi.org/10.1016/j.childyouth.2011.04.036>.

19. Cupples ME, Stewart MC, Percy A, Hepper P, Murphy C, Halliday HL. A RCT of peer-mentoring for first-time mothers in socially disadvantaged areas (the MOMENTS Study). Archives of Disease in Childhood. 2011;96(3):252-8. doi: <https://dx.doi.org/10.1136/adc.2009.167387>.

20. Walkup JT, Barlow A, Mullany BC, Pan W, Goklish N, Hasting R, et al. Randomized controlled trial of a paraprofessional-delivered in-home intervention for young reservation-based American Indian mothers. Journal of the American Academy of Child and Adolescent Psychiatry. 2009;48(6):591‐601. doi: 10.1097/CHI.0b013e3181a0ab86.

21. Jacobs F, Easterbrooks M, Goldberg J, Mistry J, Bumgarner E, Raskin M, et al. Improving adolescent parenting: results from a randomized controlled trial of a home visiting program for young families. American Journal of Public Health. 2016;106(2):342-9. doi: <http://dx.doi.org/10.2105/AJPH.2015.302919>.

22. Olds DL, Robinson J, Pettitt L, Luckey DW, Holmberg J, Ng RK, et al. Effects of home visits by paraprofessionals and by nurses: age 4 follow-up results of a randomized trial. Pediatrics. 2004;114(6):1560-8. doi: <http://dx.doi.org/10.1542/peds.2004-0961>.

23. Duggan A, Caldera D, Rodriguez K, Burrell L, Rohde C, Crowne SS. Impact of a statewide home visiting program to prevent child abuse. Child Abuse & Neglect. 2007;31(8):801-27. doi: <http://dx.doi.org/10.1016/j.chiabu.2006.06.011>.

24. Duggan A, Fuddy L, Burrell L, Higman SM, et al. Randomized trial of a statewide home visiting program to prevent child abuse: impact in reducing parental risk factors. Child Abuse & Neglect. 2004;28(6):623-43. doi: <http://dx.doi.org/10.1016/j.chiabu.2003.08.008>.

25. Rotheram-Borus MJ, Tomlinson M, Roux IL, Stein JA. Alcohol use, partner violence, and depression: a cluster randomized controlled trial among urban South African mothers over 3 years. American Journal of Preventive Medicine. 2015;49(5):715-25. doi: <https://dx.doi.org/10.1016/j.amepre.2015.05.004>.

26. Silovsky JF, Bard D, Chaffin M, Hecht D, Burris L, Owora A, et al. Prevention of child maltreatment in high-risk rural families: a randomized clinical trial with child welfare outcomes. Children and Youth Services Review. 2011;33(8):1435-44. doi: <http://dx.doi.org/10.1016/j.childyouth.2011.04.023>.

27. Jack SM, Boyle M, McKee C, Ford-Gilboe M, Wathen C, Scribano P, et al. Effect of addition of an intimate partner violence intervention to a nurse home visitation program on maternal quality of life: a randomized clinical trial. JAMA: Journal of the American Medical Association. 2019;321(16):1576-85. doi: <http://dx.doi.org/10.1001/jama.2019.3211>.

28. Ondersma SJ, Martin J, Fortson B, Whitaker DJ, Self-Brown S, Beatty J, et al. Technology to augment early home visitation for child maltreatment prevention: a pragmatic randomized trial. Child Maltreatment. 2017;22(4):334-43. doi: <http://dx.doi.org/10.1177/1077559517729890>.

29. Nagle GA. Maternal participation, depression and partner violence in a state run child abuse prevention program: Louisiana nurse home visitation, 1999–2002. Ann Arbor2002.

30. Dinmohammadi S, Dadashi M, Ahmadnia E, Janani L, Kharaghani R. The effect of solution-focused counseling on violence rate and quality of life of pregnant women at risk of domestic violence: a randomized controlled trial. BMC Pregnancy and Childbirth. 2021;21(1):221. doi: 10.1186/s12884-021-03674-z.

31. Grigg DN. An ecological assessment of the efficacy of individual and couples treatment formats of experiential systemic therapy for alcohol dependency. Ann Arbor1994.

32. Luthar SS, Suchman NE, Altomare M. Relational psychotherapy mothers' group: a randomized clinical trial for substance abusing mothers. Development and Psychopathology. 2007;19(1):243-61. doi: <http://dx.doi.org/10.1017/S0954579407070137>.

33. McWhirter PT. Differential therapeutic outcomes of community-based group interventions for women and children exposed to intimate partner violence. Journal of Interpersonal Violence. 2011;26(12):2457-82. doi: <https://dx.doi.org/10.1177/0886260510383026>.

34. Wu Q, Slesnick N. Interruption of dysfunctional mother-child reciprocal influences associated with family therapy. Journal of Family Psychology. 2019;33(7):753-63. doi: <https://dx.doi.org/10.1037/fam0000536>.

35. Stover CS. Fathers for Change for substance use and intimate partner violence: initial community pilot. Family process. 2015;54(4):600-9. doi: <http://dx.doi.org/10.1111/famp.12136>.

36. Stover CS, McMahon TJ, Moore K. A randomized pilot trial of two parenting interventions for fathers in residential substance use disorder treatment. Journal of substance abuse treatment. 2019;104:116‐27. doi: 10.1016/j.jsat.2019.07.003.

37. Suchman NE, DeCoste CL, McMahon TJ, Dalton R, Mayes LC, Borelli J. Mothering From the Inside Out: results of a second randomized clinical trial testing a mentalization-based intervention for mothers in addiction treatment. Development & Psychopathology. 2017;29(2):617-36. doi: <https://dx.doi.org/10.1017/S0954579417000220>.

38. Suchman NE, DeCoste C, Castiglioni N, McMahon TJ, Rounsaville B, Mayes L. The Mothers and Toddlers Program, an attachment-based parenting intervention for substance using women: post-treatment results from a randomized clinical pilot. Attachment & Human Development. 2010;12(5):483-504. doi: <https://dx.doi.org/10.1080/14616734.2010.501983>.

39. Zlotnick C, Capezza NM, Parker D. An interpersonally based intervention for low-income pregnant women with intimate partner violence: a pilot study. Archives of Women's Mental Health. 2011;14(1):55-65. doi: <https://dx.doi.org/10.1007/s00737-010-0195-x>.

40. Trevillion K, Ryan E, Pickles A, Heslin M, Byford S, Nath S, et al. An exploratory parallel-group randomised controlled trial of antenatal Guided Self-Help (plus usual care) versus usual care alone for pregnant women with depression: DAWN trial. Journal of Affective Disorders. 2020;261:187-97. doi: <http://dx.doi.org/10.1016/j.jad.2019.10.013>.

41. El-Mohandes AA, Kiely M, Joseph JG, Subramanian S, Johnson AA, Blake SM, et al. An intervention to improve postpartum outcomes in African-American mothers: a randomized controlled trial. Obstetrics & Gynecology. 2008;112(3):611-20. doi: <https://dx.doi.org/10.1097/AOG.0b013e3181834b10>.

42. Volpicelli J, Markman I, Monterosso J, Filing J, O'Brien C. Psychosocially enhanced treatment for cocaine-dependent mothers: evidence of efficacy. Journal of Substance Abuse Treatment. 2000;18(1):41-9. doi: <http://dx.doi.org/10.1016/S0740-5472%2899%2900024-0>.

43. Jones HE, Tuten M, O'Grady KE. Treating the partners of opioid-dependent pregnant patients: feasibility and efficacy. The American Journal of Drug and Alcohol Abuse. 2011;37(3):170-8. doi: <http://dx.doi.org/10.3109/00952990.2011.563336>.

44. Lam WK, Fals-Stewart W, Kelley ML. Parent training with behavioral couples therapy for fathers' alcohol abuse: effects on substance use, parental relationship, parenting, and CPS involvement. Child Maltreatment. 2009;14(3):243-54. doi: <https://dx.doi.org/10.1177/1077559509334091>.

45. Slesnick N, Erdem G. Efficacy of ecologically-based treatment with substance-abusing homeless mothers: substance use and housing outcomes. Journal of Substance Abuse Treatment. 2013;45(5):416-25. doi: <http://dx.doi.org/10.1016/j.jsat.2013.05.008>.

46. Rotheram-Borus MJ, Lee M, Leonard N, Lin Y, Franzke L, Turner E, et al. Four-year behavioral outcomes of an intervention for parents living with HIV and their adolescent children. Aids. 2003;17(8):1217-25. doi: <http://dx.doi.org/10.1097/00002030-200305230-00014>.

47. Rotheram-Borus MJ, Rice E, Comulada W, Best K, Elia C, Peters K, et al. Intervention outcomes among HIV-affected families over 18 months. AIDS and Behavior. 2012;16(5):1265-75. doi: <http://dx.doi.org/10.1007/s10461-011-0075-z>.

48. Tiwari A, Leung WC, Leung TW, Humphreys J, Parker B, Ho PC. A randomised controlled trial of empowerment training for Chinese abused pregnant women in Hong Kong. BJOG : an international journal of obstetrics and gynaecology. 2005;112(9):1249-56.

49. Taft AJ, Small R, Hegarty KL, Watson LF, Gold L, Lumley JA. Mothers' AdvocateS In the Community (MOSAIC)--non-professional mentor support to reduce intimate partner violence and depression in mothers: a cluster randomised trial in primary care. BMC Public Health. 2011;11:178. doi: <https://dx.doi.org/10.1186/1471-2458-11-178>.

50. Sullivan CM, Bybee DI, Allen NE. Findings from a community-based program for battered women and their children. Journal of Interpersonal Violence. 2002;17(9):915-36. doi: <http://dx.doi.org/10.1177/0886260502017009001>.

51. Fleming MF, Lund MR, Wilton G, Landry M, Scheets D. The Healthy Moms Study: the efficacy of brief alcohol intervention in postpartum women. Alcoholism: Clinical & Experimental Research. 2008;32(9):1600-6. doi: <https://dx.doi.org/10.1111/j.1530-0277.2008.00738.x>.
